# Development and validation of a CAF-related signature for prognosis and therapy response in colorectal cancer: new insights on HSPB1

**DOI:** 10.1038/s41698-025-01217-9

**Published:** 2025-12-17

**Authors:** Chaozhao Chen, Yanfei Shao, Xiaodong Fan, Huang Zheng, Tingyan Lu, Ruitian Gao, Qianru Yu, Shunan Li, Qichen Huang, Xiao Yang, Xuan Zhao, Junjun Ma, Batuer Aikemu, Minhua Zheng, Jing Sun

**Affiliations:** 1 Department of General Surgery, Ruijin Hospital, Shanghai Jiao Tong University School of Medicine, Shanghai, China; 2 Shanghai Minimally Invasive Surgery Center, Ruijin Hospital, Shanghai Jiao Tong University School of Medicine, Shanghai, China; 3 Shanghai Jiao Tong University School of Medicine, Shanghai, China; 4 Shanghai Institute of Digestive Surgery, Ruijin Hospital, Shanghai Jiao Tong University School of Medicine, Shanghai, China; 5 Fujian Medical University, FuZhou, Fujian China

**Keywords:** Gastrointestinal cancer, Tumour biomarkers

## Abstract

Colorectal cancer (CRC) is a globally prevalent malignancy with high mortality rates. Cancer-associated fibroblasts (CAFs) are crucial in CRC progression and therapeutic response. This study systematically screened 22 CAF-related prognostic genes using single-cell and spatial transcriptomics analysis. By integrating 101 combinations of 10 machine learning algorithms, we developed and validated a comprehensive predictive model (CRPS) based on large-scale public and in-house datasets (1,541 patients in total), which exhibited superior prognostic predictability compared to 58 existing CRC prognostic models. CRPS score not only effectively evaluates biological functions, immune infiltration, and gene mutation levels, but also serves as a valuable tool for predicting immunotherapy efficacy in various cohorts (478 patients in total). In-house single-cell and spatial transcriptomics data, microarray cohort analysis, and experimental validation revealed that model key gene *HSPB1* is closely associated with malignant transformation and subtype conversion of CAFs. In vitro and in vivo experiments further demonstrated that *HSPB1*-overexpressing CAFs enhance tumor cell malignancy, underscoring the therapeutic promise of targeting the *HSPB1*–CAF axis in CRC.

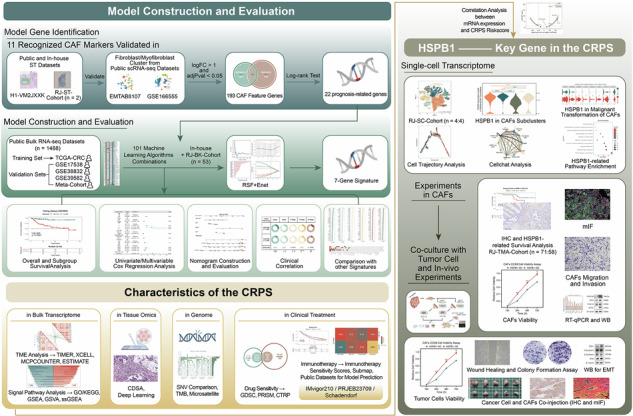

## Introduction

Colorectal cancer (CRC) is a severe malignancy with high mortality rates^1^. It currently ranks as the third most frequently diagnosed cancer worldwide and is the second leading cause of cancer-related deaths globally^2,3^, posing a significant threat to public health. Early-stage CRC often presents with non-specific symptoms^4^, which complicates timely detection. Unfortunately, as the time for diagnosis is delayed, the prognosis for CRC gradually worsens^5^. Additionally, the invasive nature and biological heterogeneity of CRC pose challenges to its treatment and management^6,7^. Therefore, the identification of personalized biological markers for risk stratification based on molecular features is urgently needed to enable effective early diagnosis and treatment, which are crucial for improving patient outcomes in CRC.

The tumor microenvironment (TME) plays a crucial role in the occurrence, development, and metastasis of CRC^8,9^, proven to be a key determinant of CRC prognosis^10^. Generally, the components of the TME include extracellular matrix (ECM) and various cell populations such as cancer-associated fibroblasts (CAFs), immune cells, and vascular endothelial cells. Among these, CAFs are one of the major components of the tumor stroma^11^. CAFs interact with tumor cells and the TME, influencing ECM remodeling, drug delivery interference, cytokine secretion, angiogenesis, and immune cell recruitment^12–15^, thereby impacting CRC initiation, progression, metastasis, and drug resistance^16,17^. Therefore, exploring the diverse functions of CAFs and its impact on CRC, along with developing reliable predictive markers linked to CAFs for identifying new therapeutic targets, are critical issues that require immediate attention. In recent years, significant advancements have been made in single-cell RNA sequencing (scRNA-seq) and spatial transcriptomics (ST) technologies. The scRNA-seq excels at discovering oncogenic cell clusters and analyzing gene expression at single-cell resolution, facilitating the investigation of TME components in cancer progression^18^. The advantage of ST lies in its ability to integrate spatial structure and transcriptomic data, analyzing the interactions and connections between TME and tumor cells^19^. The strategic use of both technologies provides a more effective approach to identifying personalized therapeutic targets and helps us better understand the various components and functions of the TME, offering new opportunities for personalized CRC treatment.

This research involves a wide range of public databases and a diverse collection of in-house datasets (RJ-CRC-Omni-Cohort). By utilizing public single-cell data along with both public and in-house spatial transcriptomics data (RJ-ST-Cohort), we identified a series of CAF-related genes and developed a CAF-related stratification risk signature using a combination of 101 machine learning algorithms. We validated the predictive efficacy of this signature in multiple public datasets as well as in our own RNA-Seq dataset (RJ-BK-Cohort). We assessed the correlation of the signature with patient prognosis, molecular biological functions, immune infiltration, single nucleotide variations (SNVs), and drug efficacy. Furthermore, through in-house single-cell RNA sequencing (RJ-ST-Cohort), tissue microarray analysis (RJ-TMA-Cohort), and in vitro experiments, we explored how *HSPB1*, a key gene in the signature, is related to the activation and malignant transformation of CAFs. These findings deepen our understanding of CAF functions within the TME and lay the groundwork for developing targeted treatment strategies for CRC patients.

## Results

### Identification and validation of CAF feature genes at single-cell and spatial transcriptomics levels

To acquire an ample pool of CAF feature genes, we conducted analyses on both spatial and single-cell transcriptomic datasets of CRC.

**Fig. 1:**
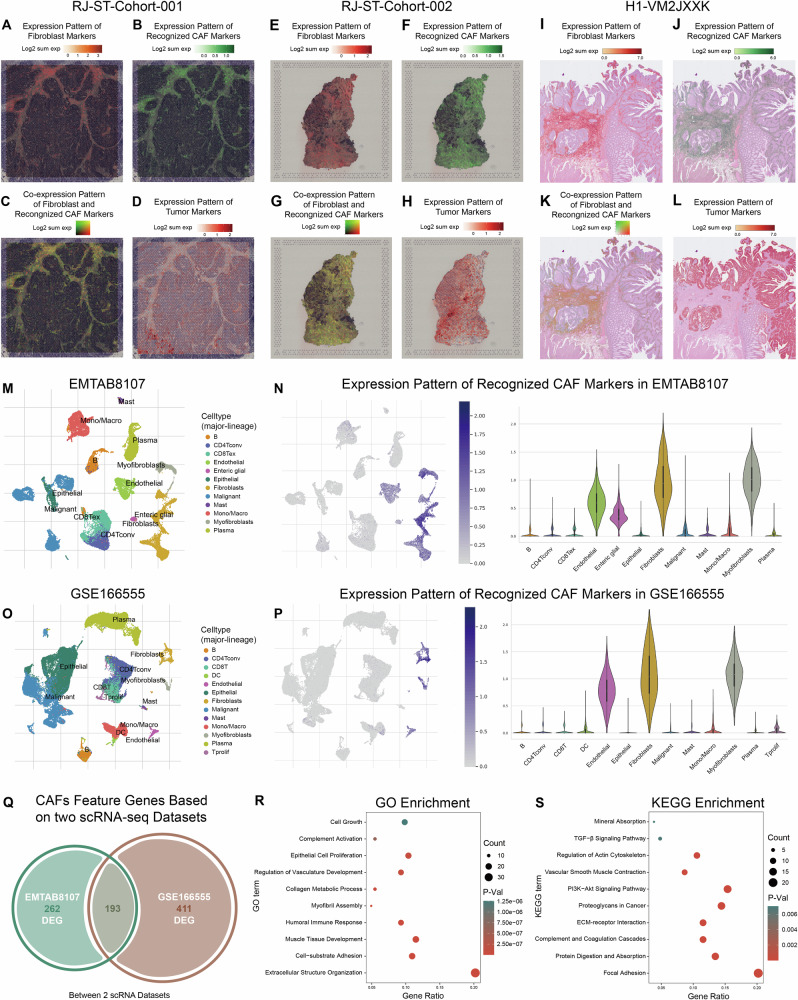
Identification and validation of CAF feature genes at single-cell and spatial transcriptomics levels. **A** Spatial distribution pattern of two fibroblast markers in RJ-ST-Cohort-001 dataset. **B** Spatial distribution pattern of eleven recognized CAF markers in RJ-ST-Cohort-001 dataset. **C** Co-expression pattern of fibroblast markers and CAF markers in RJ-ST-Cohort-001 dataset. **D** Spatial distribution pattern of four tumor markers in RJ-ST-Cohort-001 dataset. **E** Spatial distribution pattern of two fibroblast markers in RJ-ST-Cohort-002 dataset. **F** Spatial distribution pattern of eleven recognized CAF markers in RJ-CRC-002 dataset. **G** Co-expression pattern of fibroblast markers and CAF markers in RJ-ST-Cohort-002. **H** Spatial distribution pattern of four tumor markers in RJ-ST-Cohort-002 dataset. **I** Spatial distribution pattern of two fibroblast markers in H1-VM2JXXK dataset. **J** Spatial distribution pattern of eleven recognized CAF markers in H1-VM2JXXK dataset. **K** Co-expression pattern of fibroblast markers and CAF markers in H1-VM2JXXK dataset. **L** Spatial distribution pattern of four tumor markers in H1-VM2JXXK dataset. **M** Cell clusters identified with markers for each cell type generated by TISCH2 in EMTAB8107 dataset. **N** Collective expression patterns of eleven recognized CAF markers in EMTAB8107 dataset. **O** Cell clusters identified with markers for each cell type generated by TISCH2 in GSE166555 dataset. **P** Collective expression patterns of eleven recognized CAF markers in GSE166555 dataset. **Q** Venn diagram depicting the shared CAF feature genes between the EMTAB8107 and GSE166555 datasets. (**R-S**) GO (**R**) and KEGG (**S**) pathway enrichment analysis conducted with CAF feature genes.

Firstly, we obtained 11 widely recognized CAF markers *(ACTA2, FAP, PDGFRB, CAV1, PDPN, PDGFRA, ZEB1, FOXF1, SPARC, MMP2, and FN1)* based on a comprehensive literature review (Table S1). To validate the authenticity of these CAF markers, we performed spatial transcriptomics analyses on two samples from our center (RJ-ST-Cohort) along with a publicly available spatial transcriptomic dataset, H1-VM2JXXK^20^. We observed the spatial patterns of two fibroblast marker genes provided by H1-VM2JXXK (Table S2) and eleven CAF marker genes in the tissues (Fig. 1A, B, E, F and I, J). CAF markers exhibited significant overlap with fibroblast markers in all three datasets (Fig. 1C, G, K), whereas their overlap with four tumor marker genes provided by H1-VM2JXXK (Table S2) was comparatively low (Fig. 1D, H, L), providing strong evidence for the credibility of CAF markers.

Subsequently, two scRNA-seq datasets of CRC, EMTAB8107 and GSE166555, were analyzed using the TISCH2 platform. Twelve cell clusters were annotated in EMTAB8107 (Fig. 1M), while a total of thirteen cell clusters were identified in GSE166555 (Fig. 1O). In these datasets, the fibroblast and myofibroblast clusters were manually verified using the eleven CAF markers referenced earlier. Notably, the expression density of CAF markers was significantly elevated in these two cell clusters (Figs. 1N, 1P and S1), confirming the identity of these clusters as CAFs in CRC. Following this, significantly expressed feature genes of the myofibroblast and fibroblast clusters were identified in both scRNA-seq datasets, using a threshold of logFC > 1 and adjPval < 0.05.

Ultimately, 193 candidate genes (Table S3) were screened for subsequent analysis after intersecting the CAF feature genes obtained from EMTAB8107 (262 in total) and GSE166555 (411 in total) (Fig. 1Q). Subsequent GO and KEGG annotation analysis revealed that these feature genes are primarily enriched in pathways related to desmoplastic reaction and immune modulation, including focal adhesion, cell-substrate adhesion, extracellular structure organization, humoral immune response, complement activation, TGF-β signaling pathway. These enrichment patterns align with the typical functions of CAFs (Fig. 1R, S), confirming the appropriateness of these CAF feature genes for further genomic signature screening.

### Construction of the CRPS model via the machine learning-based integrative procedure

**Fig. 2:**
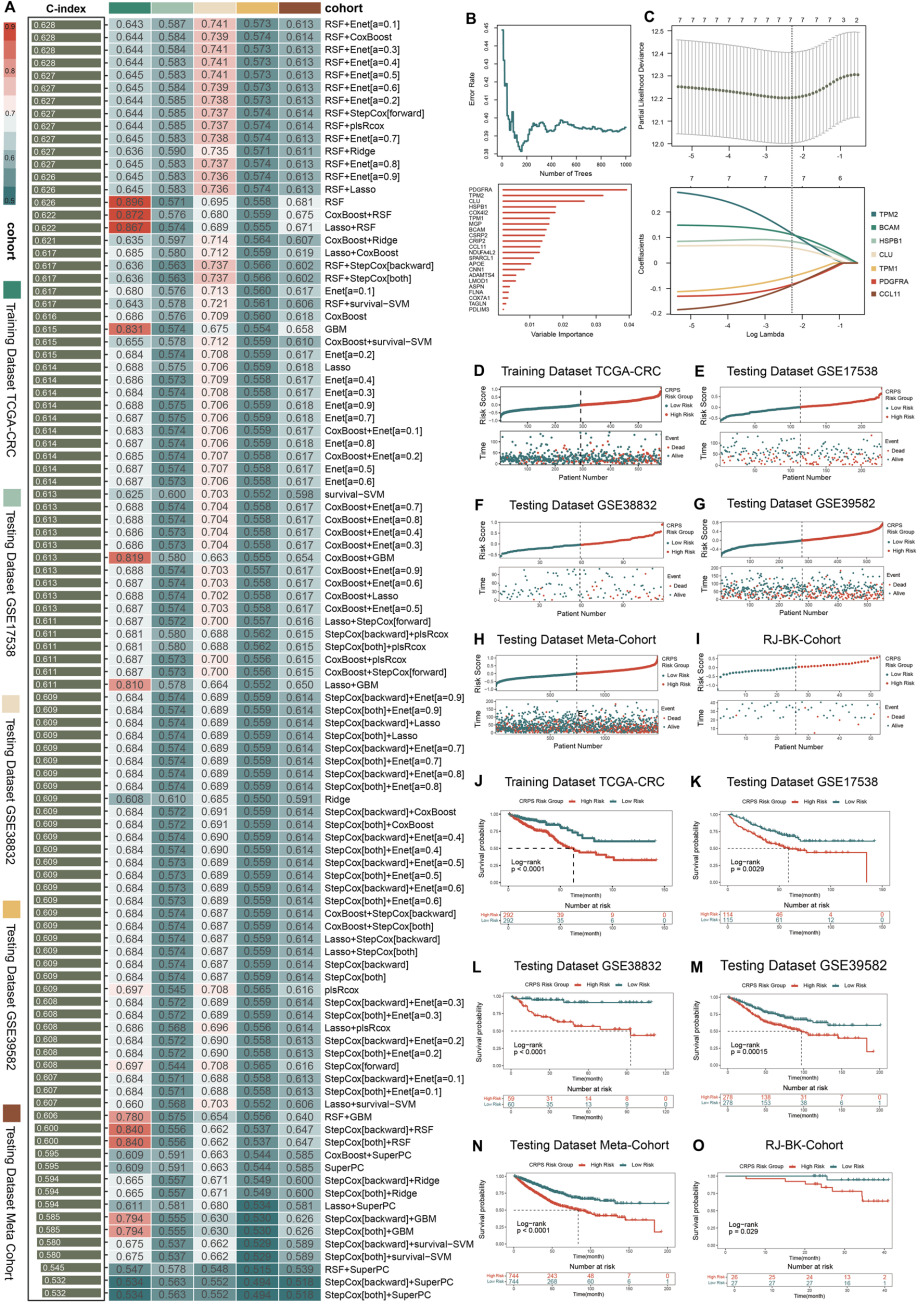
Construction of the CRPS model via the machine learning-based integrative procedure. **A** 101 kinds of prediction models evaluated via the LOOCV framework, and the C-index calculated for each model across all validation datasets. (**B**) Error rate and Variable Importance in random survival forest analysis. (**C**) Optimal λ value in TCGA-CRC cohort was determined by identifying the point where the partial likelihood deviance reached its minimum, then derived Enet coefficients for the most informative prognostic genes. **D**–**I** Distribution of patient risk scores and OS status in TCGA-CRC cohort (**D**), GSE17538 cohort (**E**), GSE38832 cohort (**F**), GSE39582 cohort (**G**), the meta-cohort (**H)**, and RJ-BK-Cohort (**I**). **J**–**O** Kaplan-Meier survival curves of CRPS risk groups regarding OS in TCGA-CRC cohort (**J**), GSE17538 cohort (**K**), GSE38832 cohort (**L**), GSE39582 cohort (**M**), the meta-cohort (**N**) and RJ-BK-Cohort (**O**).

Based on the expression profiles of 193 candidate genes, we applied a log-rank test to identify 22 genes associated with prognosis (Table S3). These 22 genes were further analyzed using a machine learning-based integrative approach to develop a CAF-related prognostic signature (CRPS). Within the TCGA-CRC dataset, we evaluated 101 prediction models using the LOOCV framework and calculated the C-index for each model across all datasets (Fig. 2A). The combination of RSF and Enet (α = 0.1) emerged as the optimal model, achieving the highest average C-index (0.628) across all public validation datasets.

Through RSF analysis, 7 genes (*PDGFRA, HSPB1, TPM2, BCAM, TPM1, CLU, CCL11*) were identified as top variables through the tree minimal depth methodology (Fig. 2B). As expected, all of these genes were shown to have significant prognostic relevance in the TCGA-CRC cohort (Fig. S2). These genes were subsequently subjected to Enet regression, where the optimal λ was determined as the partial likelihood deviance reached the minimum value (Fig. 2C). Using the regression coefficients from Enet, we calculated a risk score for each patient, enabling stratification into high-risk and low-risk groups based on the median risk score. It appears that mortality rates demonstrated a consistent upward trend corresponding to elevated risk scores in TCGA-CRC training cohort, all-four validation cohorts and our in-house RJ-BK-Cohort (Fig. 2D–I). Survival analysis showed that patients in the high-risk group exhibited significantly poorer overall survival (OS) compared to those in the low-risk group across all six cohorts (Fig. 2J-O).

### CRPS demonstrates excellent predictive performance in model evaluation and peer comparison

**Fig. 3:**
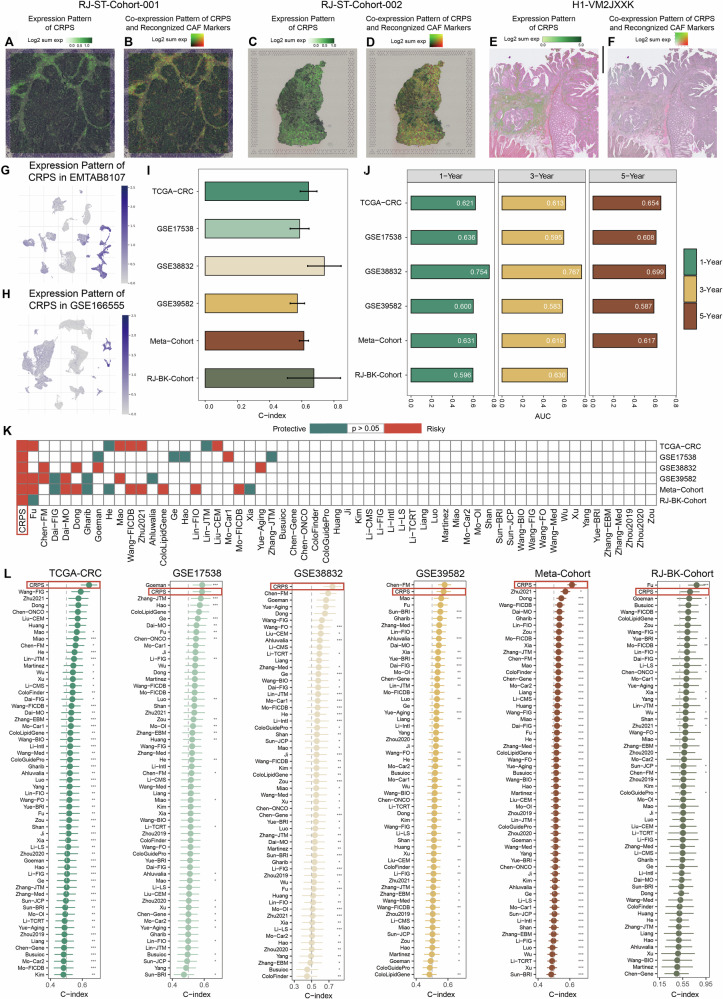
The CRPS demonstrates excellent predictive performance in model evaluation and peer comparison. **A** Spatial distribution pattern of CRPS in RJ-ST-Cohort-001 dataset. **B** Co-expression pattern of eleven established CAF markers and CRPS in RJ-ST-Cohort-001 dataset. **C** Spatial distribution pattern of CRPS in RJ-ST-Cohort-002 dataset. **D** Co-expression pattern of eleven established CAF markers and CRPS in RJ-ST-Cohort-002 dataset. **E** Spatial distribution pattern of CRPS in H1-VM2JXXK dataset. **F** Co-expression pattern of eleven established CAF markers and CRPS in H1-VM2JXXK dataset. **G**–**H** Collective expression patterns of CRPS in EMTAB8107 (**G**) and GSE166555 (**H**). **I** C-index of CRPS across all datasets. **J** Time-dependent ROC analysis for predicting OS at 1, 3, and 5 years. **K** Univariate Cox regression analysis of CRPS and 58 published signatures in TCGA-CRC, GSE17538, GSE38832, GSE39582, and meta-cohort. **L** The C-index of CRPS and 58 published models in TCGA-CRC cohort, GSE17538 cohort, GSE38832 cohort, GSE39582 cohort, the meta-cohort and RJ-BK-Cohort. For (**I**) and (**L**), data are presented as C-index ± 1.96SE. Statistical significance was calculated using two-sided z-score test. *P < 0.05; **P < 0.01; ***P < 0.001.

To assess the correlation between CRPS and CAFs, we analyzed their distribution in both in-house and publicly available spatial transcriptome datasets (Fig. 3A, C, E). Across all three datasets, CRPS exhibited considerable overlap with 11 recognized CAF markers (Fig. 3B, D, F). Subsequently, we visualized the expression patterns of CRPS across different clusters in single-cell RNA sequencing profiles (Fig. 3G, H and S3). As anticipated, CRPS were upregulated in clusters of myofibroblasts and fibroblasts within both the EMTAB8107 and GSE166555 datasets, affirming their credibility as indicators of CAFs.

The C-index [95% confidence interval (CI)] were 0.643 [0.590–0.695], 0.587 [0.528–0.645], 0.741 [0.637–0.845], 0.573 [0.528–0.618], 0.613 [0.585–0.642] and 0.676 [0.511-0.841] across the 6 cohorts (Fig. 3I), indicating a stable performance of CRPS in both public and in-house cohorts. Time-dependent Receiver Operating Characteristic (ROC) analysis was utilized to evaluate the diagnostic accuracy of CRPS over various time points, yielding 1-, 3-, and 5-year areas under the ROC curves (AUC) values of 0.621, 0.613, and 0.654 in TCGA-CRC; 0.636, 0.595, and 0.608 in GSE17538; 0.754, 0.767, and 0.699 in GSE38832; 0.600, 0.583, and 0.587 in GSE39582; 0.631, 0.610, and 0.617 in the meta-cohort and 0.596 and 0.630 in RJ-BK-Cohort (only 1- and 3- year AUC values available) (Fig. 3J). These ROC analyses indicate CRPS exhibits robust predictive efficacy in the public dataset at 1, 3, and 5 years, while also demonstrating strong predictive performance in the in-house cohort at 1 and 3 years.

To compare the performance of CRPS with other prognostic signatures developed by machine learning, we obtained 58 previously published CRC signatures associated with diverse biological processes (Table S4). Univariate Cox regression indicated that only CRPS exhibited significant association with prognosis in all 6 cohorts (Fig. 3K). Furthermore, the C-index comparison revealed that CRPS outperformed nearly all other models in every cohort, including our own RJ-BK-Cohort, underscoring its reliability and superior robustness (Fig. 3L).

### CRPS serves as an independent prognostic factor in constructing a nomogram with high predictive performance

To evaluate the effectiveness of the CRPS risk score in predicting survival across various clinical characteristics, we conducted stratified analyses based on age ( < 65 vs. ≥65), gender (male vs. female), tumor site (left-side vs. right-side), and AJCC stage (I-II vs. III-IV). The results demonstrated that individuals classified in the low-risk group exhibited significantly superior OS compared to those in the high-risk group within each subgroup (Fig. S4A). These results underscore a reliable predictive capability of the CRPS risk score across diverse patient profiles.

Univariate and multivariable Cox regression analyses were then performed to determine the prognostic value of the CRPS risk score, along with other clinical variables. Univariate Cox regression analysis identified that the risk score (P < 0.001, HR = 4.72, 95%CI = 2.56–7.47), as well as key clinical parameters including age (P = 0.003, HR = 1.84, 95%CI = 1.23–2.75), AJCC stage (P = 0.003, HR = 4.13, 95%CI = 1.63–10.50; P < 0.001, HR = 10.67, 95%CI = 4.20–27.13), and tumor site (P = 0.035, HR = 0.67, 95%CI = 0.47-0.97) were significantly associated with OS in TCGA-CRC cohort (Fig. S4B). Multivariable Cox regression further confirmed that the risk score (P < 0.001, HR = 2.64, 95%CI = 1.49-4.68), along with age (P = 0.002, HR = 2.01, 95%CI = 1.30–3.09), AJCC stage (P = 0.011, HR = 3.47, 95%CI = 1.33–9.00; P < 0.001, HR = 10.60, 95%CI = 4.09–27.44), and tumor site (P = 0.003, HR = 0.56, 95%CI = 0.38–0.82), independently influenced OS (Fig. S4C).

Subsequently, by integrating age, AJCC stage, tumor site, and the risk score, we developed a nomogram prediction model for TCGA-CRC cohort (Fig. S4D) to forecast survival probabilities at 1-, 3-, and 5-years. Calibration curves demonstrated the accurate predictive performance of the model across these time points (Fig. S4E). Moreover, the nomogram model demonstrated superior ability in predicting 1-, 3-, and 5-year survival outcomes, as evidenced by higher AUC scores compared to the risk score alone (Figs. S4F and 3J). In summary, CRPS exhibits an independent survival predictive capability beyond the influence of various clinical factors and holds the potential to be integrated with other clinical characteristics to develop a more effective predictive model.

### CRPS exhibits strong correlations with clinicopathological features through various molecular mechanisms

**Fig. 4:**
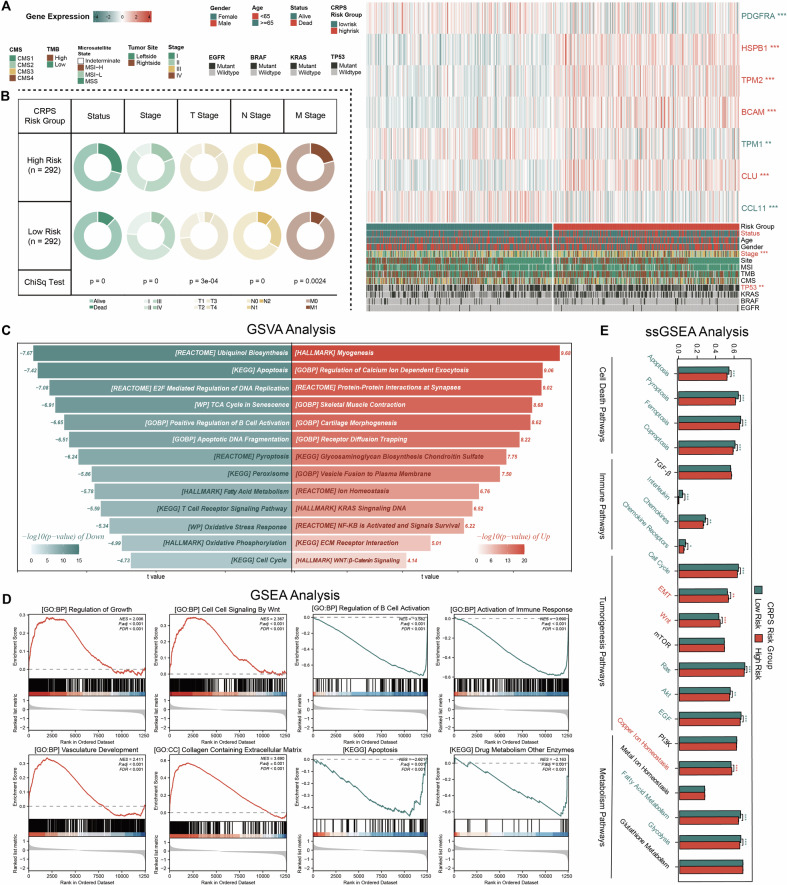
CRPS exhibits strong correlations with clinicopathological features through various molecular mechanisms. **A** Differences in mRNA expression levels of CRPS genes and clinicopathologic features between high (n = 292) and low (n = 292) CRPS risk groups. **B** Circos plot of different survival and pathological factors between high (n = 292) and low (n = 292) risk groups in TCGA-CRC cohort. **C** Differences in MsigDB-based pathway activities between two risk groups scored by GSVA. **D** GO and KEGG terms enriched with DEGs between two risk group by GSEA analysis. **E** Differences in multiple biological pathway activities between high (n = 292) and low (n = 292) risk groups scored by ssGSEA. Statistical significance was calculated using Wilcoxon test (**A**–**E**) and Chi-square test (**A**, **B**). *P < 0.05; **P < 0.01; ***P < 0.001.

To explore the roles of CRPS risk scores in patients with CRC, we examined the expression levels of CRPS genes and their association with clinical characteristics using a heatmap (Fig. 4A). Significant differences were observed in the expression levels of 7 CRPS genes between the two risk groups. Furthermore, the CRPS risk score showed strong correlations with survival status, AJCC stage, and TNM stage in TCGA-CRC cohort (Figs. 4B and S5A-S5D). In GSE17538 and GSE38832 cohorts, the high-risk group exhibited markedly worse prognosis and more advanced stages compared to the low-risk group (Figs. S5E-S5F and S5O-S5P). Similarly, in the GSE39582 cohort, the high-risk group exhibited a significant worsening of prognosis, AJCC stage, and T/M staging compared to the low-risk group (Figs. S5G-S5J and S5Q). Additionally, in our in-house cohort, there were notable differences in AJCC/N staging and survival outcomes between two risk groups (Figs. S5K-S5N and S5R).

To delve deeper into the biological distinctions between the high and low-risk groups, we conducted GO, KEGG, GSVA, and GSEA analyses. The results highlighted notable differences in cell-response, tumorigenesis, immune response, and metabolism-related pathways. Specifically, pathways such as vasculature development, cell cycle checkpoint signaling, oxidative stress response, apoptosis, p53 signaling, Wnt signaling, *KRAS* signaling, B cell activation, complement activation, T cell receptor signaling, ion homeostasis, drug resistance, and fatty acid metabolism were prominently affected (Fig. 4C, D and S6). Additionally, significant differences were observed in ssGSEA enrichment scores between the two risk groups across various pathways, including cell death, immune response, tumorigenesis, and metabolism (Fig. 4E). In conclusion, the CRPS risk score appears to be intricately connected to multiple biological mechanisms.

### Differences in somatic mutations and tumor immune microenvironment are observed between high and low CRPS risk groups

**Fig. 5:**
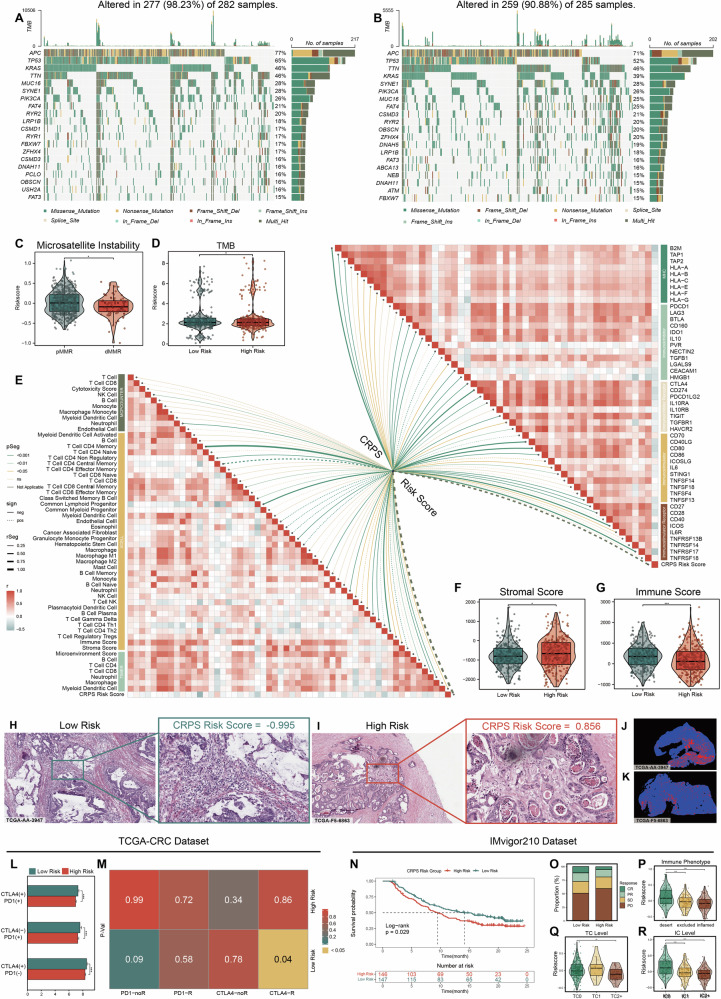
Significant variations in gene somatic mutations, tumor immune microenvironment, and immunotherapy responses exist between high and low CRPS risk groups. **A**, **B** Waterfall plots showing genetic alterations of common mutant genes in high (**A**) and low (**B**) CRPS risk groups in TCGA-CRC cohort. **C** Violin plot showing variations in CRPS risk scores between two microsatellite subtypes (n = 500 in pMMR group; n = 81 in dMMR group). **D** Violin plot showing variations in TMB scores between high (n = 275) and low (n = 264) risk groups. **E** Butterfly plot displaying the correlation between CRPS risk scores and immune scores & between CRPS risk scores and mRNA expression of different kinds of immune checkpoints. **F**, **G** Violin plot showing variations in stromal scores and immune scores between high (n = 292) and low (n = 292) risk groups. **H,** **I** Representative images showing pathological HE staining from low (**H**) and high (**I**) CRPS risk groups in TCGA-CRC cohort. **J**, **K** Representative images displaying tumor-infiltrating lymphocytes density in pathological HE staining from high (**J**) and low (**K**) CRPS risk groups in TCGA-CRC cohort analyzed by the deep learning technology. **L** Histograms showing variations in predicted immunotherapy sensitivity scores between high (n = 292) and low (n = 292) CRPS risk groups in TCGA-CRC cohort. **M** Prediction of responses of two risk groups samples in TCGA-CRC cohort to immunotherapies using submap analysis. **N** Kaplan-Meier survival curves of CRPS risk groups regarding OS in IMvigor210 cohort. **O** Stacked histogram showing variations in the proportion of different anti-PD-L1 responsiveness between two risk groups in IMvigor210 cohort. **P** Violin plot shows variations in CRPS risk score among three immune phenotypes in IMvigor210 cohort (n = 69 in desert group; n = 109 in excluded group; n = 62 in inflamed group). **Q**, **R** Violin plots showing variations in CRPS risk score among samples with different PD-L1 expression levels in TC (**Q**) and IC (**R**) in IMvigor210 cohort (n = 81 in IC0 group; n = 109 in IC1 group; n = 102 in IC2+ group; n = 234 in TC0 group; n = 16 in TC1 group; n = 42 in TC2+ group). Statistical significance was calculated using Wilcoxon test (**C**, **D**, **F,** **G**, **L** and **P**–**R**). *P < 0.05; **P < 0.01; ***P < 0.001.

To determine whether there were differences in gene mutations between high-risk and low-risk groups, we accessed and analyzed SNV data of TCGA-CRC cohort. Summarized gene mutation frequencies were displayed through bar plots (Fig. S7A, B and Fig. 5A, B). In the high-risk group, the most frequently mutated genes were *APC* (77%), *TP53* (65%), *KRAS* (46%), *TTN* (46%), and *MUC16* (28%), while in the low-risk group, *APC* (71%), *TP53* (52%), *TTN* (46%), *KRAS* (39%), and *SYNE1* (28%) showed highest mutation rates. Specifically, while *TP53* showed notable mutation rates in both groups, it also exhibited significantly higher mutation frequencies in the high-risk group compared to the low-risk group, as depicted in lollipop plots (Fig. S7C, D). In addition, we used a forest plot to depict clear differences in gene mutation profiles, revealing other genes with significantly higher mutation rates in high- or low-risk group (Fig. S7E). Additionally, microsatellite instability (MSI) and tumor mutation burden (TMB) were assessed for their association with CRPS risk score. There was a trend indicating lower risk scores in the dMMR group (Fig. 5C and Table S5) and higher TMB in the high-risk group (Fig. 5D), suggesting potential connections between microsatellite status, TMB, and CRPS.

Functional enrichment analyses showed significant differences in immune processes between risk groups. Therefore, TIMER, XCELL, and MCPCOUNTER tools were used to evaluate immune cell infiltration levels based on RNA sequencing data from TCGA-CRC cohort. Moreover, correlations between CRPS risk score and expression levels of immune checkpoint genes were explored and displayed through butterfly plots, heatmaps, and boxplots (Figs. 5E and S8A, B). Results indicated that a lower CRPS risk score correlated with increased infiltration of various immune cell types including B cells, T cells, CD4+ memory T cells, CD8 + T cells, neutrophils, and myeloid dendritic cells. Furthermore, expression of MHC, immune-stimulators, immune-stimulator receptors, immune-inhibitors, and immune-inhibitor receptors markers showed negative associations with CRPS risk score. ESTIMATE analysis revealed that samples in the low-risk group exhibited higher immune scores and lower stromal scores (Fig. 5F, G).

To further validate the relationship between immune infiltration and CRPS, Hematoxylin-eosin (HE) staining data from TCGA-CRC cohort and RJ-BK-Cohort were used to assess histopathological features in samples with different risk scores. Tissue sections with lower risk scores exhibited enhanced lymphocyte infiltration in both public and in-house cohorts (Figs. 5H, I and S9A, B). Using deep learning technology developed by Saltz et al.^21^, lymphocyte infiltration data from HE pathological images were extracted, the presence of lymphocytes was evaluated and similar result was obtained (Fig. 5J, K). These analyses collectively indicated that the low-risk group had higher rates of immune cell infiltration.

### CRPS demonstrates excellent predictive capability for immunotherapy response

Immunotherapies have gradually taken on a more prominent role in clinical practice. Given the molecular and clinical significance of the CRPS risk score in CRC, particularly its correlation with immune cell infiltration, we analyzed multiple immunotherapy-related cohorts to evaluate CRPS as a predictor of treatment response across various cancer types.

In TCGA-CRC cohort, our analysis revealed significant differences in predicted immunotherapy sensitivity scores between high- and low-risk groups across various immunotherapy strategies (Fig. 5L). Furthermore, SubMap analysis suggested that low-CRPS risk samples in the TCGA-CRC cohort exhibited transcriptomic features more similar to those of anti‑CTLA‑4 responders in the reference immunotherapy dataset (Fig. 5M).

In other immunotherapy datasets, such as the IMvigor210 cohort, patients with high risk score exhibited markedly worse OS compared to those with low risk scores (Fig. 5N). On the other hand, those with higher risk scores were more likely to experience poorer clinical responses to anti-PD-L1 immunotherapy (Fig. 5O). Moreover, samples with the immune-desert phenotype had a significantly higher risk score compared to those with immune-excluded and immune-inflamed phenotypes (Fig. 5P). We further analyzed the correlation between the risk score and the immune types of tumor-infiltrating immune cells (IC) and tumor cells (TC) (Fig. 5Q, R). Our findings revealed a negative correlation between the risk score and PD-L1 expression in both immune cells and tumor cells. In the PRJEB23709 cohort, the risk score was also shown to be predictive of progression-free survival (PFS) and correlated with clinical response to anti-PD-1 and anti-CTLA-4 immunotherapies (Fig. S10A, B). Similarly, in the Schadendorf cohort, patients in the high-risk group exhibited shorter PFS and worse treatment outcomes (Fig. S10C, D). Collectively, the CRPS risk score developed in this study represents a potentially robust tool for predicting prognosis and immunotherapy responses across diverse cancer types.

### Drug sensitivity varies significantly between high and low CRPS risk groups

Several studies have underscored the strong link between CAFs and resistance to cancer therapies^22,23^. Therefore, we analyzed the resistance profiles of human cancer cell lines (CCLs) utilizing the CTRP and PRISM datasets. After removing duplicates and incomplete data, we selected 355 compounds from CTRP and 1,286 from PRISM for further analysis (Fig. S11A). Using a ridge regression model, we predicted the area under the dose-response curve (AUC) values for various drugs in TCGA-CRC samples based on AUC values and expression data from CRC cell lines and purified tumor expression profiles from TCGA-CRC cohorts. The top and bottom 20% of samples were then selected based on their risk scores, and their corresponding AUC values were compared. Initially, we identified compounds that had significantly higher predicted AUC values in the top group (logFC > 0.15). Subsequently, we performed Spearman correlation analysis between AUC values and CRPS risk scores to further screen the identified compounds, focusing on those showing a positive correlation coefficient (R > 0.25 for CTRP, or R > 0.3 for PRISM) (Fig. S11B, C). This process yielded six compounds from CTRP (KX2-391, Austocystin D, CR-1-31B, GSK461364, SB-743921, Paclitaxel) and six from PRISM (KX2-391, Verubulin, CA4, HCPT, TPA, Sirolimus) (Fig. S11D). These candidate compounds, particularly KX2-391, may not be suitable for treating high-risk group patients.

To explore the relationship between CRPS and drug sensitivity, we analyzed the expression profiles of all CRC cell lines in the GDSC datasets and stratified them into high- and low-risk groups based on the expression of CRPS-related genes. Expressions of CRPS-related genes in TCGA-CRC cohort were z-score centered and utilized to develop a nearest centroid classifier for predicting gene-classified clusters. This classifier was then applied to CRC cell lines to predict CRPS risk groups. The GDSC database contains a total of 398 compounds, of which only 248 tested on more than 90% of CRC cell lines were used for analysis. Comparing the AUC values of drug responses and IC50 values between two risk groups (Fig. S11E–G), we observed significantly higher AUC values for 5-Fluorouracil in the high-risk group. Additionally, the AUC values and IC50 values of AZ960, APO886, Elesclomol, YM-155 and AS605240 were significantly lower in high-risk CRC cells, whereas those of Nintedanib, WYE-125132 and Phenformin were significantly higher. The results above suggest distinct sensitivities to various therapeutic approaches between high- and low-risk CRC groups.

### Identification of two CRPS-related molecular phenotypes by unsupervised learning

To comprehensively explore the role of CRPS in individuals with CRC, we identified two distinct CRPS-related molecular phenotypes through unsupervised learning analysis (379 cases in Cluster A and 205 cases in Cluster B) (Fig. S12A). The sankey diagram revealed that Cluster A had a higher overlap with the high-risk group, while Cluster B was more aligned with the low-risk group (Fig. S12B). Prognostic analysis revealed that patients in Cluster A had poorer outcomes compared to those in Cluster B (Fig. S12C). Additionally, the TMB levels in Cluster A were significantly higher than in Cluster B (Fig. S12D). We also conducted GSVA analysis to gain a clearer understanding of the biological differences between these two phenotypes, and the results highlighted that the biological discrepancies were primarily associated with interleukin-23 production, plasma cell differentiation, oxidative stress response, drug metabolism, DNA replication, apoptosis, cell cycle regulation and ion homeostasis pathways (Fig. S12E). Furthermore, significant variations were observed in immune checkpoint expression, immune cell infiltration, ESTIMATE scores (Fig. S12F, G) and immunotherapy sensitivity scores (Fig. S12H–J) between the two phenotypes, reflecting patterns similar to those of high- and low-risk groups. These results indicate that the CRPS-related molecular phenotypes serve as an effective tool for distinguishing the various characteristics of CRC patients.

### Single-cell profiling reveals proportional shifts in cell subtypes between high and low CRPS risk groups

**Fig. 6:**
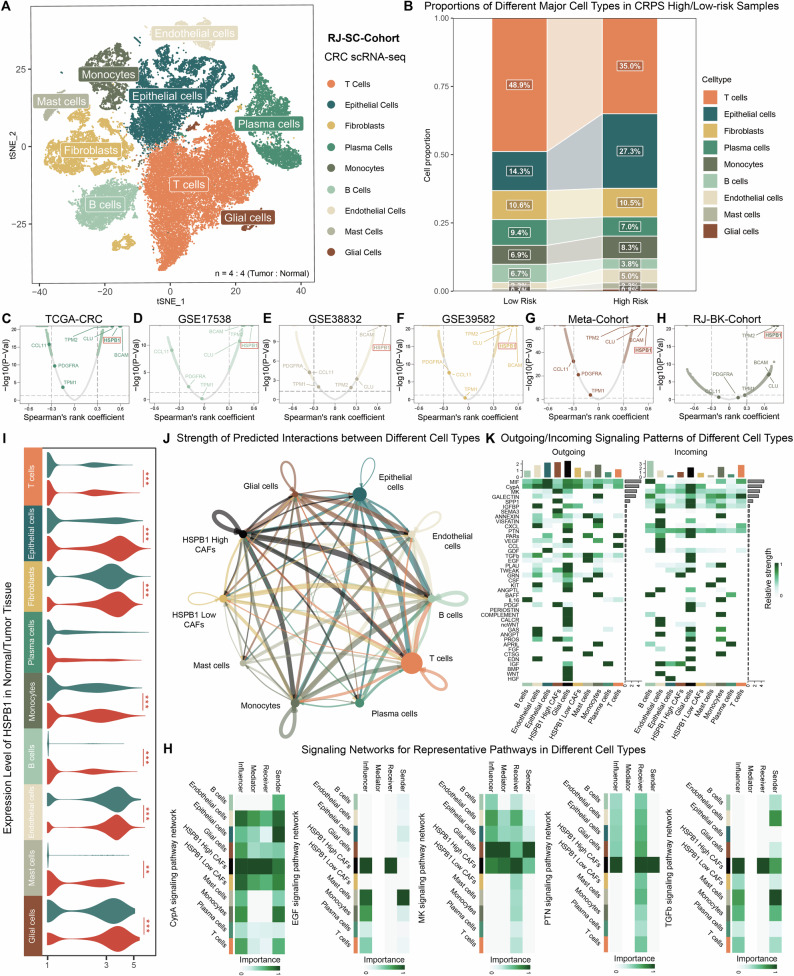
Single-cell analysis provides evidence for the involvement of CRPS and the key gene HSPB1 in shaping the TME of CRC. **A** t-SNE plot showing characterization of 50,831 cells profiled in CRC and normal tissues. **B** Stacked histogram showing the relative abundances of different major cell types in samples with high (n = 2) and low (n = 2) CRPS risk score. **C**–**H** Volcano plot showing correlations between CRPS risk scores and mRNA expression in TCGA-CRC cohort (**C**), GSE17538 cohort (**D**), GSE38832 cohort (**E**), GSE39582 cohort (**F**), meta-cohort (**G**) and RJ-BK-Cohort (**H**). **I** Violin plot showing *HSPB1* expression in tumor and normal tissues across different cell clusters (n = 12,088, 7417, 3149, 2383, 2358, 1572, 1185, 495, 157 in tumor group; n = 5828, 3020, 3302, 2636, 845, 2897, 600, 275, 624 in normal group). **J** Prediction of cell–cell communication among different cell clusters. The thickness of each line indicates the weight of predicted interactions. **K** Predicted afferent and efferent signaling pathways across different cell types. **H** Signaling networks for representative pathways including CypA, EGF, MK, PTN, and TGFβ, showing sender, receiver, mediator, and influencer cell types. Statistical significance was calculated using Spearman correlation analysis (**C**–**H**) and Wilcoxon test (**I**). *P < 0.05; **P < 0.01; ***P < 0.001.

To validate the findings from the bulk-seq analyses and further assess the role of CRPS in CRC TME, we conducted droplet-based scRNA-seq on four treatment-naïve CRC tissue samples and their corresponding normal control colon tissues from patients in our patient group (RJ-SC-Cohort), including two from CRPS high-risk group and two from CRPS low-risk group. After stringent quality control, 50831 high-quality single cells were selected for further analysis. Using graph-based clustering of merged and normalized cells, we identified distinct clusters characterized by specific canonical markers, including T cells (*CD3D* + , *IL7R* + ), epithelial cells (*EPCAM* + , *KRT18* + ), fibroblasts (*COL1A1* + , *DCN* + ), plasma cells (*IGHA1* + , *IGLC2* + ), monocytes (*LYZ* + , *CD14* + , *S100A8* + ), B cells (*CD79A* + , *MS4A1* + ), endothelial cells (*PECAM1* + , *VWF* + ), mast cells (*CPA3* + , *KIT* + ), and glial cells (*CDH19* + , *SOX10* + , *PLP1* + )^24,25^ (Fig. 6A and S13A–C). Notably, this in-house scRNA-seq dataset further confirmed that both the eleven established CAF markers and CRPS were predominantly expressed in fibroblasts across all cell types (Fig. S14).

We conducted further clustering and subgroup analysis of a total of 30,804 cells within the CRC tissue samples (Fig. S15A and S15C, D). Given the predominant fibroblast-specific expression of CRPS, we next investigated whether tumors with different CRPS levels displayed distinct cellular compositions within the TME (Figs. 6B and S15B). Overall, the proportion of CAFs was similar between CRPS high-risk and low-risk samples, whereas epithelial and endothelial cells were markedly enriched in CRPS high-risk tumors. In contrast, immune infiltration was substantially reduced in CRPS high-risk tumors—most notably within T cells and B cells (including plasma cells)—a pattern consistent with the bulk-seq findings. These results suggest that the CRPS score is largely determined by the presence and activity of CAFs, which may play a pivotal role in shaping the TME in CRC.

### Centering on HSPB1 as the key component of CRPS

To explore the genes involved in forming CRPS through a more targeted manner, correlation analyses were conducted between mRNA expression levels of all genes and CRPS risk scores across all cohorts (Fig. 6C–H). Genes with their correlation coefficients exceeding 0.30 (P < 0.05) were deemed significant. In all 6 cohorts, *HSPB1, BCAM*, and *CLU* emerged as significant genes. Notably, *HSPB1* exhibited the highest average ranking, with correlation coefficients exceeding 0.59 across all cohorts, suggesting its pivotal role in the CRPS model. Examination at the public single-cell (Fig. S16A, B) and spatial transcriptomic levels (Fig. S16C–E) revealed that *HSPB1* is widely expressed in both tumor cells and stroma of CRC.

We next observed that *HSPB1* expression was significantly elevated in epithelial cells, fibroblasts, endothelial cells and glial cells, while it was low in immune cells such as T cells, B cells, and monocytes (Figs. 6I and S13D). The expression of *HSPB1* in different tumor cell clusters also followed the aforementioned trend (Fig. S15E). Comparison of *HSPB1* expression between tumor and normal epithelial tissues indicated that *HSPB1* was upregulated in malignant epithelial cells. Furthermore, *HSPB1* levels in CAFs were significantly higher than in normal fibroblasts (NFs) (Fig. 6I), suggesting its potential association with fibroblast malignant transformation.

Given the distinct expression pattern of *HSPB1* in fibroblasts, we next sought to explore the potential functional consequences of *HSPB1* overexpression in CAFs. CellChat analysis was performed to characterize intercellular communication networks among various cell types within TME. Using the Secreted Signaling ligand–receptor interaction database, we systematically mapped signaling interactions across all major cell populations. This analysis uncovered widespread communication among different cell types (Figs. 6J and S17A), with particularly strong and frequent interactions involving *HSPB1*^high^ CAFs (Fig. 6K). Compared with *HSPB1*^low^ CAFs, the *HSPB1*^high^ CAFs displayed markedly increased interaction intensity with epithelial cells and diverse immune cell subsets (Fig. S17G, H), indicating enhanced signaling crosstalk within the TME. Pathway-level analysis further identified several representative signaling pathways—such as CypA, EGF, MK, PTN, and TGFβ—that were notably enriched in *HSPB1*^high^ CAF–TME interactions (Figs. 6H and S17B–F). These results suggest that *HSPB1* overexpression may endow CAFs with a more active signaling role, potentially facilitating tumor-promoting communication and remodeling of the TME.

### The crucial role of HSPB1 in the malignant transformation and subtype conversion of CAFs supported by single-cell analysis

**Fig. 7:**
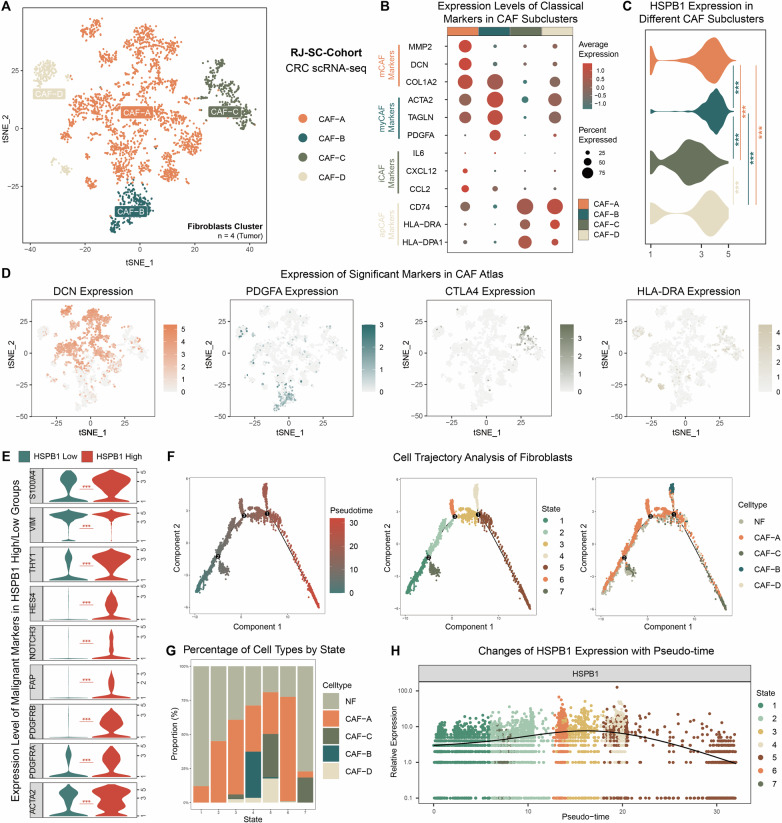
The role of HSPB1 in the malignant transformation and subtype conversion of CAFs supported by single-cell analysis. **A** t-SNE plot of fibroblasts cluster with cells classified into CAF-A, CAF-B, CAF-C and CAF-D subtypes. **B** Dot plot showing expression of recognized marker genes in different CAF subtypes. **C** Violin plot of showing expression of *HSPB1* across all CAF subtypes (n = 2276 in CAF-A group; n = 305 in CAF-B group; n = 412 in CAF-C group; n = 255 in CAF-D group). **D** t-SNE plot showing variable expression of significant markers in different CAF subtypes. **E** Violin plot showing expression of recognized malignant fibroblast markers in *HSPB1*-high and *HSPB1*-low CAFs (n = 1,651 in *HSPB1*-High group; n = 1597 in *HSPB1*-Low group). **F** The cell trajectory analysis of fibroblasts. **G** Stacked histogram showing percentage of different cell types by state. **H** The changes of *HSPB1* expression with pseudo-time in fibroblasts. Statistical significance was calculated using Wilcoxon test (**C** and **E**). *P < 0.05; **P < 0.01; ***P < 0.001.

Through the analysis of our in-house single-cell data, we further explored the relationship between *HSPB1* and CAFs. Upon re-clustering and analyzing 3,248 high-quality fibroblasts, we classified CAFs into four subtypes: CAF-A, CAF-B, CAF-C and CAF-D (Fig. 7A). CAF-A cells exhibited elevated expression of *MMP2, DCN* and *COL1A2*, markers of matrix CAFs (mCAFs) known for their role in ECM remodeling^26^ (Fig. 7B, D and S18A, B). CAF-B cells highly expressed *ACTA2, TAGLN* and *PDGFA*, typical markers of myofibroblast-like CAFs (myCAFs)^26^, as well as pericyte markers such as *CSPG4, RGS5* and *PDGFRB*, suggesting characteristics of vascular CAFs (vCAFs)^27^ (Figs. 7B, D and S18A, B). CAF-C showed close association with immune functions, expressing immune checkpoint markers like *CTLA4* and *TIGIT*, as well as various other immune-related markers (Fig. 7D and S18A, B). Finally, CAF-D exhibited high expression of antigen-presenting markers including *CD74, HLA-DRA* and *HLA-DPA1*^28^ (Fig. 7B, D and S18A, B).

Studies indicate that activated CAFs express elevated levels of *HSPB1*, which is crucial for fibroblast adhesion, contractility, and motility^29,30^. In RJ-BK-Cohort, we observed that the expression levels of *HSPB1*, genes that are highly expressed in CAFs compared to NFs (such as *ACTA2, PDGFRA, PDGFRB, FAP, NOTCH3, HES4, THY1, VIM* and *S100A4*)^31,32^, as well as CAF-A and CAF-B markers, were markedly elevated in CRPS high-risk group compared to the CRPS low-risk group (Fig. S18C). We further investigated expression level of *HSPB1* across CAF subtypes, finding a significantly lower expression level in CAF-C (Fig. 7C). Additionally, malignant markers in fibroblasts were notably upregulated in CAFs with high *HSPB1* expression (Fig. 7E). To further explore the biological differences between *HSPB1*^high^ CAFs and *HSPB1*^low^ CAFs, GO and KEGG analyses on the differentially expressed genes (DEGs) identified between these two clusters were performed. The findings revealed that in the *HSPB1*^high^ cluster there are an upregulation of pathways linked to fibroblast activation, fibroblast proliferation, vascular smooth muscle cell proliferation, response to oxidative stress, regulation of ion transport and ECM organization as well as a downregulation of pathways related to immune response and immune checkpoints (Fig. S18D). GO and KEGG enrichment analyses of DEGs were also performed between *HSPB1*^high^ and *HSPB1*^low^ epithelial cells. The results revealed that *HSPB1*^high^ epithelial cells were enriched in pathways related to oxidative phosphorylation, protein refolding, and regulation of protein ubiquitination, while *HSPB1*^low^ epithelial cells were enriched in epithelial structure maintenance, apoptosis, and immune-related pathways (Fig. S19A, B).

Cell trajectory analysis was also carried out to explore the involvement of *HSPB1* in the development and differentiation of CAFs (Fig. 7F). NFs and CAFs subpopulations were pooled and classified into seven states, with significant differences in the proportions of CAF subtypes within each state. Specifically, NFs had the highest proportion in State 1, CAF-A had the highest proportion in State 6. In State 4, CAF-B had relatively higher proportions compared to other states, similar to the situation of CAF-C and CAF-D in State 5 (Fig. 7G). As pseudo-time progressed, fibroblasts gradually completed the transition from NFs to various CAF subtypes, while the expression level of *HSPB1* exhibited a trend of initially increasing followed by a subsequent decrease (Fig. 7H). The results above revealed the important role of *HSPB1* in the activation and differentiation of CAFs.

### HSPB1 overexpression promotes worse prognosis in CRC and enhances malignant characteristics in CAFs

**Fig. 8:**
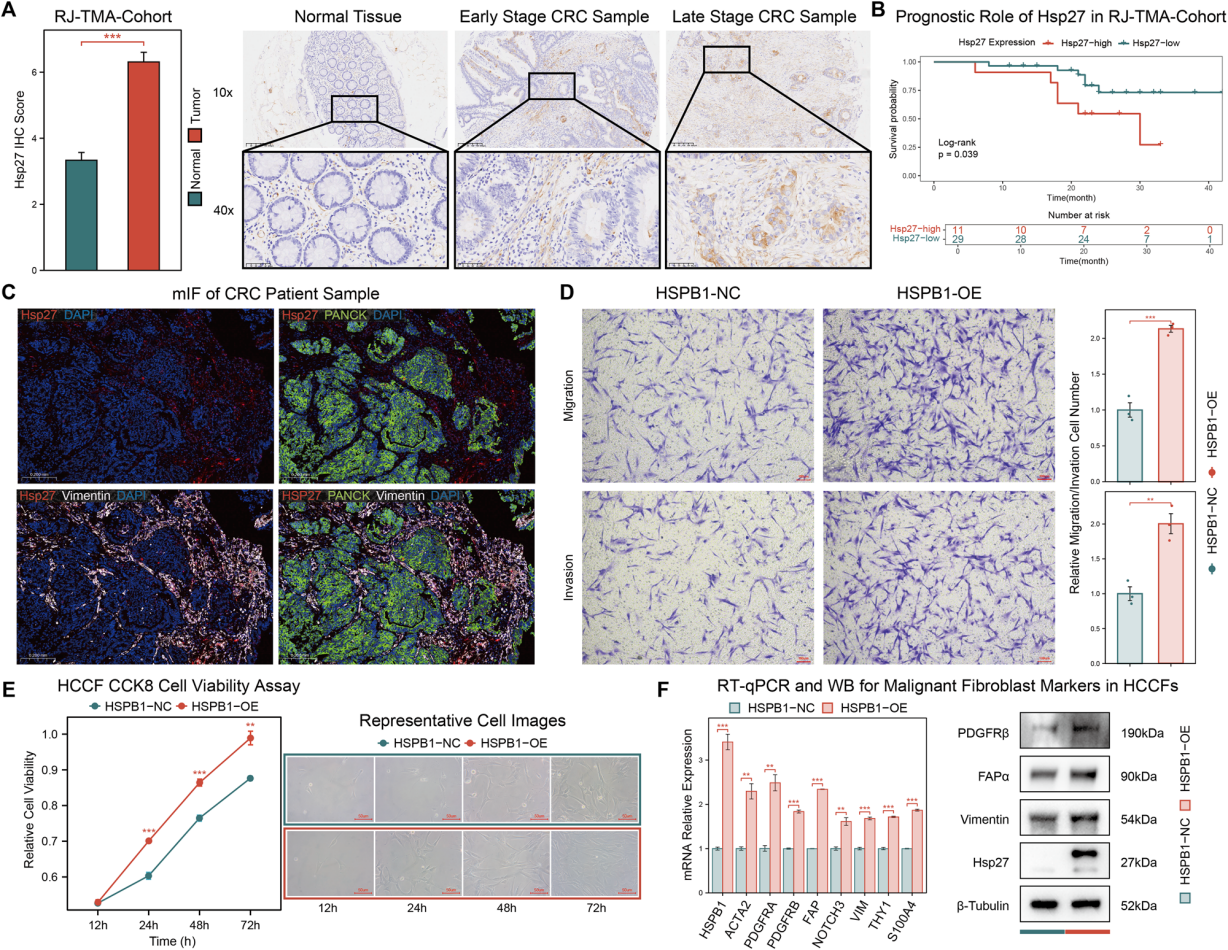
HSPB1 overexpression promotes worse prognosis in CRC and malignant characteristics in CAFs. **A** Quantification and representative images of *HSPB1* protein (Hsp27) immunohistochemistry staining in CRC tumor and paired normal samples (n = 76 in tumor group; n = 51 in normal group; Scale bar indicates 200 and 50 μm). **B** Kaplan-Meier survival curves of Hsp27 regarding OS in RJ-TMA-Cohort. **C** Representative mIF images of CRC patient samples. DAPI (blue), Hsp27 (red), PANCK (green), and Vimentin (white) (Scale bar indicates 200μm). **D** Representative images and quantification of transwell migration and invasion assay in HCCFs with *HSPB1* overexpression (n = 3 in *HSPB1*-OE group; n = 3 in *HSPB1*-NC group, Scale bar indicates 100μm). **E** Cell proliferation assays using CCK-8 in HCCFs with *HSPB1* overexpression (n = 4 in *HSPB1*-OE group; n = 4 in *HSPB1*-NC group), accompanied by representative images of cells at indicated time points (Scale bar indicates 50μm). **F** RT-qPCR analysis were performed to evaluate mRNA expression of 9 gene in *HSPB1*-NC and *HSPB1*-OE HCCFs, including *HSPB1, FAP, ACAT2, NOTCH3, PDGFRA, PDGFRB, VIM, THY1*, and *S100A4* (n = 3 in *HSPB1*-OE group; n = 3 in *HSPB1*-NC group). Western Blot analyses were performed to evaluate differential protein expression of HSP27 and recognized malignant fibroblast markers in HCCFs. Statistical significance was calculated using Wilcoxon test (**A** and **D**–**F**). *P < 0.05; **P < 0.01; ***P < 0.001.

To ensure the scientific rigor and reliability of the findings above, we performed a series of in vitro validation experiments and prognostic analyses. First, we assessed *HSPB1* protein expression in CRC samples and their paired normal tissues obtained from our center (RJ-TMA-Cohort). *HSPB1* protein (Hsp27) levels were significantly elevated in CRC tissues compared to normal tissues. Moreover, its expression showed a progressive increase as the tumor stage advanced, including in both tumor and stromal components (Fig. 8A). We further conducted prognostic analyses in RJ-TMA-Cohort, and discovered that samples with high Hsp27 expression had significantly poorer OS compared to those with low Hsp27 expression (Fig. 8B). To verify the localization of Hsp27 expression, we conducted multiplex immunofluorescence (mIF) experiments using Vimentin and PANCK as markers for stromal and epithelial cells, respectively (Figs. 8C and S20A, C). The results demonstrated that Hsp27 was expressed both in stromal cells and in epithelial cells. Subsequently, to investigate the functional effects of *HSPB1*, we overexpressed *HSPB1* in CAFs (HCCF), which significantly enhanced the migration and invasion abilities of CAFs (Fig. 8D). Additionally, *HSPB1* overexpression was found to promote cell proliferation (Fig. 8E). To further confirm the role of *HSPB1* in promoting the malignant transformation and of CAFs, we performed RT-qPCR and Western blot (WB) analysis to analyze changes of related markers at mRNA and protein levels. The overexpression of *HSPB1* significantly increased the mRNA expression of various malignant fibroblast markers in CAFs. Consistently, key proteins associated with malignant transformation in CAFs such as FAPα (*FAP*), Vimentin (*VIM*) and PDGFRβ (*PDGFRB*) were increased in HSPB1‑overexpressing CAFs (Fig. 8F).

### Co-culture with HSPB1-overexpressing CAFs promotes malignant phenotypes of tumor cells in vitro and in vivo

**Fig. 9:**
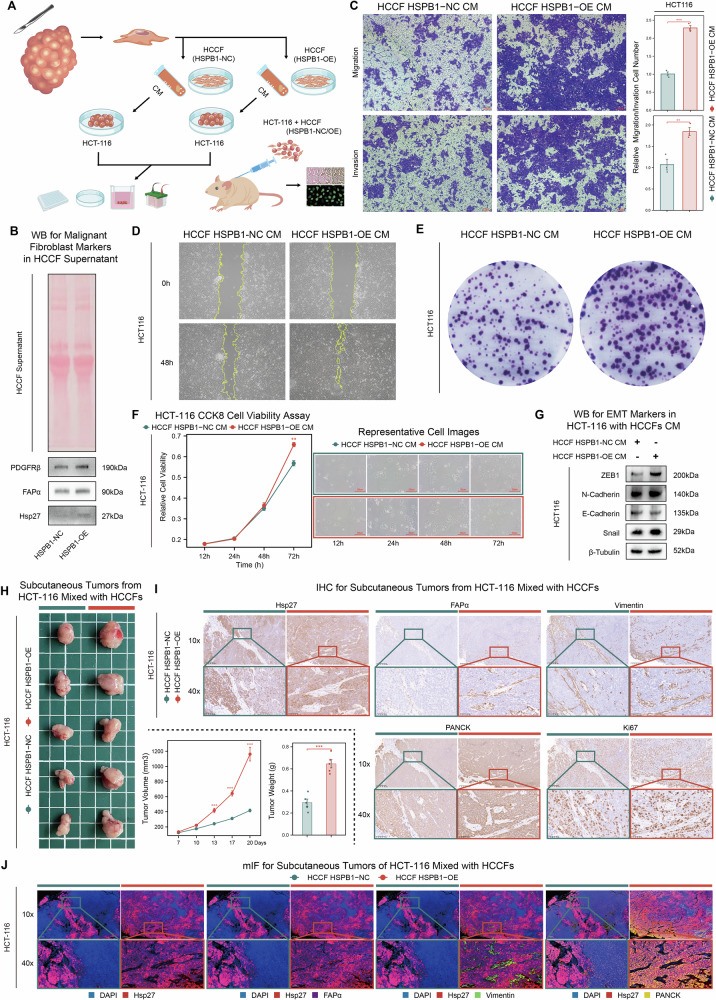
Co-culture with HSPB1-overexpressing CAFs promotes malignant phenotypes of tumor cells in vitro and in vivo. **A** Schematic diagram illustrating the co-culture experimental design. CM derived from HCCFs with *HSPB1* overexpression (HCCF *HSPB1*‑OE CM) or negative control (HCCF *HSPB1*‑NC CM) were collected and applied to HCT‑116 cells for functional assays in vitro and subcutaneous xenograft experiments in vivo. **B** WB analysis of Hsp27, FAPα and PDGFRβ in the supernatants of *HSPB1*‑NC and *HSPB1*‑OE HCCFs. **C** Representative images and quantification of transwell migration and invasion assays in HCT‑116 cells treated with HCCF HSPB1‑NC CM compared with HCCF *HSPB1*‑OE CM (n = 3 in HCT‑116 with HCCF *HSPB1*-NC CM group; n = 3 in HCT‑116 with HCCF *HSPB1*-OE CM group). **D** Representative wound‑healing images of HCT‑116 cells after treatment with HCCF *HSPB1*-NC/OE CMs for 48 h. **E** Representative images of colony formation assays of HCT‑116 cells treated with HCCF *HSPB1*-NC/OE CMs. **F** Cell proliferation assays using CCK-8 in HCT‑116 cells treated with HCCF *HSPB1*-NC/OE CMs (n = 4 in in HCT‑116 with HCCF *HSPB1*-NC CM group; n = 4 in HCT‑116 with HCCF *HSPB1*-OE CM group), accompanied by representative images of cells at indicated time points (Scale bar indicates 50μm). **G** WB analysis of EMT‑related markers (ZEB1, N‑cadherin, E‑cadherin, and Snail) in HCT‑116 cells treated with HCCF *HSPB1*-NC/OE CMs. **H** Images of subcutaneous xenograft tumors derived from HCT‑116 cells treated with HCCF HSPB1‑NC/OE CMs, with corresponding tumor growth curves and tumor weights (n = 5 in in HCT‑116 with HCCF *HSPB1*-NC CM group; n = 5 in HCT‑116 with HCCF *HSPB1*-OE CM group). **I** Representative immunohistochemistry staining of Hsp27, FAPα, Vimentin, PANCK, and Ki‑67 in mice subcutaneous xenograft tumors from HCT‑116 cells mixed with HCCF HSPB1‑NC/OE (Scale bar indicates 200 and 50μm). **J** Representative mIF images of mice subcutaneous xenograft tumors from HCT‑116 cells mixed with HSPB1‑NC/OE HCCF. DAPI (blue), Hsp27 (red), FAPα (purple), Vimentin (green), and PANCK (yellow) (Scale bar indicates 200 and 50 μm). Statistical significance was calculated using Wilcoxon test (**C,** **F** and **H**). *P < 0.05; **P < 0.01; ***P < 0.001.

Given that *HSPB1* overexpression endowed CAFs with more aggressive characteristics, we next investigated whether *HSPB1*-overexpressing CAFs could influence the malignant phenotype of CRC epithelial cells (HCT-116) through in vitro and in vivo experiments (Fig. 9A). Conditioned media (CM) collected from *HSPB1*-overexpressing CAFs (HCCF *HSPB1*-OE CM) or negative control cells (HCCF *HSPB1*-NC CM) were applied to HCT-116 cells for functional assays. WB analysis of the cell supernatant revealed that *HSPB1*‑overexpressing CAFs secreted higher levels of Hsp27 and malignant fibroblast markers, including PDGFRβ and FAPα (Fig. 9B), indicating enhanced fibroblast activation and paracrine potential. Compared with HCCF *HSPB1*-NC CM, HCCF *HSPB1*-OE CM markedly enhanced the migratory, invasive, and proliferative capacities of HCT-116 cells, as evidenced by transwell, wound-healing, colony formation, and CCK-8 assays (Fig. 9C–F). WB analyses further revealed that treatment with HCCF *HSPB1*-OE CM led to elevated expression of mesenchymal markers (ZEB1, N-cadherin, and Snail) and decreased expression of E-cadherin in HCT-116 cells, indicating that *HSPB1*-overexpressing CAFs induce epithelial-mesenchymal transition (EMT) in tumor cells (Fig. 9G).

To further corroborate these findings in vivo, HCT-116 cells were co-injected subcutaneously with *HSPB1*-OE or *HSPB1*-NC HCCFs into nude mice. Co-injection with *HSPB1*-OE HCCFs resulted in significantly accelerated tumor growth and increased tumor weight compared to control groups (Fig. 9H). Immunohistochemistry (IHC) analyses of xenograft tumors showed markedly increased Hsp27 expression within the stromal regions of tumors co-injected with HSPB1‑OE HCCFs compared to the control group. In parallel, the expression of FAPα and Vimentin was also elevated, indicating enhanced CAF activation. Notably, epithelial regions of these tumors exhibited stronger PANCK and Ki‑67 staining, suggesting increased epithelial proliferation and tumor growth (Fig. 9I). Consistent with the IHC results, mIF further confirmed the elevated stromal expression of Hsp27, FAPα, and Vimentin together with enhanced epithelial PANCK signals in tumors co-injected with HSPB1‑OE HCCFs, highlighting the potential for dynamic interactions between stromal HSPB1-overexpressing CAFs and tumor epithelial cells (Figs. 9J and S21A, E).

## Discussion

The AJCC staging system is widely utilized in cancer management and provides a standardized framework for assessing cancer progression, enhancing comparability in tumor assessment across diverse healthcare settings and facilitating consistent treatment approaches globally^33^. Furthermore, innovations in single-cell and spatial transcriptome technologies allow us to further explore the intra-tumor molecular biological differences at the individual level^34,35^, thus enabling a better scientific stratification of patients. Recent developments in machine learning algorithms have opened new avenues to fully utilize these advantages^36^. Multiple studies have developed various prognostic signatures related to CRC, but few have been thoroughly validated^37^. In light of this, our study presents a notable advancement in this field by incorporating a robust validation framework that utilizes multiple datasets, including in-house bulk RNA-seq, single-cell RNA-seq, and spatial transcriptomics data, along with analyses supported by experimental validation results. This comprehensive approach not only enhances the reliability of our model but also enables a more in-depth exploration of key CAF-related genes, setting the stage for more personalized therapeutic interventions in CRC patients.

During the process of model construction, to reduce the risk of overfitting^38^, we excluded the training set when calculating the average c-index, enhancing the reliability of our evaluation. Ultimately, we constructed a prognostic signature (CRPS) consisting of seven genes (*BCAM, CCL11, CLU, HSPB1, PDGFRA, TPM1* and *TPM2*) using the RSF and Enet (α = 0.1) machine learning algorithms on a dataset of 1,488 public CRC samples. This signature was successfully validated for its prognostic effectiveness in our internal validation cohort. Additionally, univariate Cox analysis and c-index comparisons revealed that CRPS outperformed 58 other prognostic signatures for CRC. It also demonstrated significant predictive capability across various clinical subgroups, including age, sex, tumor site, and AJCC stage. Furthermore, CRPS serves as an independent prognostic factor, suitable for constructing a comprehensive nomogram that integrates other clinical characteristics to predict survival outcomes in CRC patients.

Regarding the molecular characteristics, the CRPS low-risk group exhibits significant upregulation of pathways related to immunity, programmed cell death, ion metabolism, oxidative stress, and epithelial-to-mesenchymal transition, all of which are linked to ferroptosis^39–45^. Ferroptosis is a novel cell death mechanism characterized by iron accumulation and lipid peroxidation, which results in reduced antioxidant capacity and subsequent cell death^46^. Evidence suggests that exosomes derived from CAFs containing *METTL3* can inhibit ferroptosis in tumor cells in CRC^47^. In addition to the pathway alterations, the mutation rate of the *TP53* gene is markedly diminished in the CRPS low-risk group. *TP53* encodes the transcription factor p53, crucial for maintaining genomic integrity; however, mutations in this gene can lead to tumorigenesis^48^. Inhibiting p53 activity in fibroblasts can facilitate the development of the CAF phenotype^49^, while its overexpression in CAFs can reduce tumor growth and enhance apoptosis in adjacent tumor cells^50^. Furthermore, p53 is linked to ferroptosis in a bidirectional manner. It promotes ferroptosis by inhibiting *SLC7A11* or enhancing the expression of *SAT1* and *GLS2*, while also suppressing ferroptosis by reducing *DPP4* activity or inducing *CDKN1A*/p21 expression^51–55^. The potential mechanisms linking CAFs, TP53 and ferroptosis warrant further investigation.

Furthermore, it is widely recognized that a significant portion of the TME is composed of immune cells^56^. To better understand the relationship between CRPS and immune cells, we compared immune infiltration between the high and low CRPS risk groups. Our findings indicate that immune cells such as T cells, B cells, NK cells, and dendritic cells are significantly decreased in the high-risk group. Pathological HE staining, along with deep learning analysis of lymphocyte infiltration in the high and low CRPS risk groups, showed a similar trend. We also observed that multiple immune checkpoint markers, including *CTLA4* and *PDCD1LG2*, tend to be highly expressed in the low-risk group. Tumor cells often exploit immune checkpoint proteins to evade immune surveillance, and higher expression of these markers correlates with better outcomes for immunotherapy^57,58^. In TCGA-CRC cohort, differences in predicted immunotherapy sensitivity scores between the high and low-risk groups coincide with the expression trends of immune checkpoint markers. Given that there is currently no available immunotherapy data specifically for CRC, we utilized data from other immunotherapy datasets. SubMap analysis indicated that the gene expression patterns of TCGA-CRC low-risk group samples align with those in samples from Riaz cohort^59^ showing enhanced response to anti-CTLA-4 treatment. The CRPS risk score also demonstrated robust predictive powers in the IMvigor210, PRJEB23709, and Schadendorf cohorts. The immune phenotypes of solid tumors can be categorized into three types: immune-inflamed, immune-excluded, and immune-desert^60^. Based on our findings—where we observed significant differences in overall CRPS risk scores among samples with different immune types, along with pathological HE staining images from TCGA-CRC and our in-house cohort—we suggest that the high-risk group may predominantly exhibit an immune desert phenotype, while the low-risk group is more likely to represent an immune-inflamed phenotype.

Due to the poor response of CRPS high-risk patients to immunotherapy, we aimed to identify effective therapeutic agents for this population. By analyzing the public datasets of cancer cell lines from PRISM and CTRP, we further clarified that KX2-391 is not suitable for treating high-risk patients. KX2-391, also known as Tirbanibulin, is a highly selective Src kinase inhibitor that has been approved for the treatment of actinic keratosis and psoriasis^61^. It has demonstrated extensive antitumor activity across various cancer types^62^, and several early clinical trials have investigated its application in multiple tumors, including acute myeloid leukemia^63^, prostate cancer^64^, and other solid tumors^65^. Previous research has demonstrated that the combination of specific *FGFR* inhibitor BLU-554 with KX2-391 can significantly inhibit the metastasis of *ELF4*-overexpressing CRC compared to the single-agent therapy of KX2-391^66^, providing a potential treatment option for high-risk patients. Furthermore, the analysis of the GDSC cancer cell database revealed that the efficacy of certain drugs is significantly distinct between high-risk and low-risk groups, such as traditional chemotherapeutics like 5-Fluorouracil, which show poor effectiveness in high-risk groups. A prior study has shown that CAF can promote malignant phenotypes in CRC through circRNAs that are highly expressed in secreted exosomes, leading to resistance to traditional chemotherapeutics, including 5-Fluorouracil^67^. We also found that AZ960, APO866, Elesclomol, YM-155, and AS605240 exhibit better therapeutic effects for patients in the high-risk group. In summary, the CRPS model established in this study provides a solid foundation for more effective drug screening and personalized treatment, thereby improving the therapeutic outlook for high-risk groups.

Beyond the predictive and therapeutic implications of the model, an additional observation emerged from our analyses concerning the distribution pattern of CAF-associated genes. Specifically, the overall expression pattern of 11 well-recognized CAF markers was not strictly confined to fibroblast populations. Interestingly, the collective expression of these markers showed mild elevation in endothelial and glial cells. A similar trend was also evident for our identified CRPS, which was predominantly expressed in fibroblasts but displayed slight upregulation in endothelial compartments. Such expression overlap is unlikely to result from annotation inaccuracies or from the limited specificity of individual markers, as both single-cell and spatial transcriptomic analyses confirmed that the collective expression pattern of CRPS genes aligns closely with fibroblast distributions. Rather, this phenomenon might instead reflect the underlying functional diversity that exists among CAF subpopulations. Given the well-recognized heterogeneity in the origins and functions of CAFs—including their established roles in angiogenesis^68^, extracellular-matrix remodeling^69^, and neuro-stromal interactions^70^—the partial activation of CAF-related genes in endothelial or glial cells could represent shared functional programs within the TME. The endothelial-associated expression observed for the CRPS may in part result from its component genes such as *BCAM* and *HSPB1*, both of which are known to be expressed in endothelial cells^71,72^. Moreover, previous studies have reported *BCAM⁺* CAF subtypes with distinct transcriptional and functional profiles^28^, suggesting that the elevated expression of *BCAM* within the CRPS signature could reflect increased proportion of a CAF subset in the TME, instead of a higher prevalence of non‑CAF cells that inherently express *BCAM*. Collectively, these observations support that the CRPS retains fibroblast specificity while encompassing markers corresponding to multiple CAF subtypes, reflecting the heterogeneity and functional complexity of the tumor stroma.

To further analyze the differences in CRPS gene expression patterns among patients, we classified TCGA samples using consensus clustering, dividing them into Cluster A and Cluster B. We observed a high overlap between the clustering groups and CRPS risk groups. Significant distinctions were found in prognosis, TMB, immune infiltration, pathway enrichment differences, and response to immunotherapy, all of which aligned with the trends in the CRPS high and low groups. This indicates a close association between the CRPS risk score and the expression levels of CRPS-related genes, with *HSPB1* exhibiting the strongest correlation. *HSPB1*, also known as *Heat Shock Protein Family B Member 1*, encodes a member of the small heat shock protein family. Its expression has been linked to cancer progression and therapy resistance in several malignancies^73^. Notably, *HSPB1* plays a critical role in the iron metabolism of fibroblasts^74^ and has been identified as a negative regulator of ferroptosis in tumors^75^. The *HSPB1* protein is highly expressed both intracellularly and in the ECM^76^. Given the close relationship between *HSPB1* and CRC as well as CAFs, we believe it is a key component of CRPS that warrants further exploration. According to its distribution pattern in our in-house single-cell and spatial transcriptomic data, we found that *HSPB1* is widely expressed in various cell types within the TME. We discovered that in mIF, the expression level of *HSPB1* in the stroma is higher than that in tumor epithelial cells, which conflicts with the results from single-cell analysis. We speculate that several factors may contribute to this discrepancy: 1) There may be unique regulatory mechanisms at the protein level for *HSPB1* in different cell types that affect its expression and stability; 2) Cytokines and signaling interactions within the immune microenvironment may regulate *HSPB1* protein expression; 3) There may be individual differences among patients. This highlights the need for a more in-depth investigation in the future.

To facilitate a more profound investigation into the relationship between CAFs and *HSPB1*, we conducted a clustering analysis of CAFs based on our sc-RNA seq data. Considering the diverse types of CAFs and the lack of a unified standard for sub-classification, we validated CAF subtypes in our data using some well-recognized CAF subtype markers in CRC^26–28^. Notably, iCAF markers, such as *IL6* and *CXCL12*, exhibited low expression levels in every cell population. We also observed that CAF-A cells demonstrated high expression of *ADH1B*. Previous studies have indicated that *ADH1B* expression in CRC CAFs can inhibit *IL6* expression through its involvement in retinol metabolism^77^, leading us to suspect this might explain why iCAF subtype was failed to be identified in our CAF samples. Nevertheless, we found that CAF-C cells exhibited high expression of immune checkpoint markers such as *TIGIT* and *CTLA4*. This type of CAF may emerge as a potential target for immunotherapy in the future. Subsequently, we assessed *HSPB1* expression levels across various CAF subtypes and found that they were lowest in CAF-C, while significantly elevated in CAF-A and CAF-B. Our bulk RNA-seq analysis further revealed notable differences in the expression of various CAF markers between CRPS high- and low-risk groups, suggesting a distinct variation in CAF subtypes in each group. Additionally, cell trajectory analysis indicated that as fibroblasts transitioned from NFs to distinct CAF subtypes, the expression levels of *HSPB1* underwent significant changes. These findings lead us to hypothesize that alterations in *HSPB1* expression may facilitate the transition between CAF subtypes.

Prior research has shown that activated fibroblasts highly express *HSPB1*, which is crucial for the adhesion, contraction, and motility functions of CAFs^29,30^. The expression of *HSPB1* is significantly positively correlated with the expression of *ATCA2* in the CRC stroma, a typical marker of fibroblast activation^78^. Additionally, studies have indicated that the malignant transformation of fibroblasts is linked to increased expression of angiogenesis-related factors^79,80^, while CAF-B exhibits elevated expression of vCAF-related markers, along with the highest levels of *HSPB1* expression. Based on these knowledges, we selected several key markers that are significantly expressed in CAFs compared to NFs. We found substantial differences in the mRNA expression levels of these markers between *HSPB1-*high and *HSPB1-*low CAFs, which were then confirmed at protein levels. Pathway enrichment analysis revealed that *HSPB1-*high CAFs showed enhanced functions related to ECM secretion, intercellular adhesion, angiogenesis and regulation of iron metabolism, while exhibiting reduced immune-related functions. Notably, these *HSPB1*-expression associated pathways in CAFs differed from those observed in epithelial cells, including oxidative phosphorylation, protein refolding, apoptosis, and epithelial structure maintenance, suggesting that *HSPB1* plays distinct functional roles in CAFs from tumor epithelial cells. Experimental results indicate that overexpression of *HSPB1* enables CAFs to exhibit enhanced invasion and migration abilities, as well as increased cell proliferation. This suggests *HSPB1* plays a vital role in the malignant transformation of CAFs, indicating that *HSPB1* may actively modulates the functional changes in fibroblasts themselves. Thus, it is essential to further elucidate the mechanisms by which *HSPB1* promotes the fibroblast malignant transformation and drives CAFs subtype conversion. In addition, WB analysis of the CAF‑conditioned medium revealed markedly elevated levels of secreted *HSPB1* (Hsp27) together with malignant fibroblast markers such as FAPα and PDGFRβ. These observations led us to speculate that *HSPB1* might activate CAFs within the TME through an autocrine‑like mechanism, while the released Hsp27 could further participate in crosstalk with other stromal or epithelial cell populations, thereby potentially influencing the broader TME.

Another line of evidence supporting the role of *HSPB1* in remodeling tumor‑stroma interactions comes from cell communnication analysis, which revealed markedly strengthened intercellular communication between *HSPB1*^high^ CAFs and multiple cellular compartments within the TME. This finding suggests that *HSPB1* overexpression not only accompanies CAF malignant transformation but also enhances their ability to influence surrounding cells through active signaling exchange. The enriched signaling pathways, including CypA, EGF, MK, PTN, and TGFβ, have been implicated in diverse oncogenic processes in TME—such as modulation of immune suppression, stimulation of angiogenesis, promotion of EMT, and development of therapeutic resistance^81–84^. For instance, a PTCH‑1‑interacting peptide could inhibit CAF‑derived TGF‑β production, thereby alleviating fibrosis and enhancing immune cell infiltration^85^. Moreover, the PTN–SDC1 signaling interaction between CAFs and cancer cells plays a key role in driving bladder cancer progression^86^. Together, these findings strongly suggest that *HSPB1*-overexpressing CAFs actively reshape the TME by reinforcing cell-cell commmunication, thereby sustaining an environment conducive to tumor progression. Hence, disrupting *HSPB1*-mediated signaling programs may represent an effective strategy to mitigate the pro-tumorigenic functions of CAFs in CRC.

Consistent with the strengthened intercellular communication revealed by our single‑cell analysis, the co‑culture experiments further confirmed that *HSPB1*‑overexpressing CAFs exert pro‑tumorigenic effects on CRC epithelial cells. These findings suggest that *HSPB1*‑mediated fibroblast malignant transformation not only enhances the intrinsic aggressiveness of CAFs but also enables them to promote epithelial plasticity and malignant transformation through paracrine signaling. In vivo, tumor cells co‑injected with *HSPB1*‑OE CAFs exhibited augmented stromal activation and epithelial proliferation, suggesting that *HSPB1*-driven CAFs and cancer cells engage in dynamic interactions that contribute to a pro-tumorigenic microenvironment. Mechanistically, *HSPB1* may facilitate this process by stabilizing stress-response proteins or regulating the secretion of signaling molecules such as TGFβ, PTN, and EGF. Targeting this *HSPB1*‑dependent stromal–epithelial axis could thus be a promising therapeutic approach to counteract CAF-induced tumor progression in CRC.

From a translational perspective, the CRPS model exhibits promising clinical potential, as it was validated mainly using large-scale bulk RNA‑seq data and could be adapted for conventional assays such as RT‑qPCR or immunohistochemistry to facilitate clinical application. Nevertheless, our research does have some limitations. For instance, the CRPS model was developed based on retrospective cohorts from multiple databases, which means that additional prospective studies are necessary to confirm its clinical significance. Second, further exploration using single-cell and spatial transcriptomic techniques is needed to address the potential influences of non-CAF cell types on CRPS expression levels. Moreover, the downstream molecular mechanisms through which *HSPB1* mediates CAF malignant activation and subtype conversion remain to be fully elucidated. Also, the interactions and communication between *HSPB1*‑overexpressing CAFs and other cellular components within the TME warrant further experimental investigation.

In summary, through the integration of multiple machine learning algorithms and bioinformatics tools, our study developed a comprehensive CAF‑related signature that effectively predicts prognosis, tumor progression, and therapeutic responses in CRC. We further identified *HSPB1* as a key CRPS gene involved in the subtype conversion and malignant transformation of CAFs, and demonstrated that *HSPB1*‑overexpressing CAFs promote malignant phenotypes of tumor cells both in vitro and in vivo, offering a valuable target for future clinical interventions of CRC patients.

## Methods

### Collection and pre-processing of CRC patient samples

A total of 129 formalin-fixed and paraffin-embedded (FFPE) CRC patient specimens were collected from Ruijin Hospital, Shanghai Jiao Tong University School of Medicine from Jan. 2015 to Dec. 2023. Tumor and para-tumoral samples were obtained during surgery, embedded in optimal cutting temperature compound, and stored frozen at −80 °C until further processing. All procedures involving human subjects were conducted in accordance with the Declaration of Helsinki, and written informed consent was obtained from all patients whose tissue samples were used. This study was approved by the Ethics Committee of Ruijin Hospital, Shanghai Jiao Tong University School of Medicine (Ethics Approval number: NCT04714814).

These specimens constitute the RJ-CRC-Omni-Cohort. Among them, 53 specimens were collected for bulk RNA-sequence (RJ-BK-Cohort). 4 patient paired-samples were utilized as single-cell RNA sequence (scRNA-seq) specimens (RJ-SC-Cohort). 2 patient tumor samples were utilized as spatial transcriptomic (ST) specimens (RJ-ST-Cohort). Tissue samples collected from 76 patients were designated for tissue microarrays as previously mentioned (RJ-TMA-Cohort)^87^. To evaluate the prognostic value of CRPS, clinicopathological information from patients in RJ-BK-Cohort was collected to construct a comprehensive clinical database. The survival information and clinical data from RJ-TMA-Cohort were collected for a comprehensive prognostic analysis of *HSPB1*.

scRNA-seq was conducted using the 10X Genomics Single Cell 5’ Platform. Tumor samples underwent enzymatic dissociation (Miltenyi), followed by filtration through a 70-micron cell strainer. After centrifugation at 300 xg, the cells were pelleted and resuspended in DAPI-FACS buffer (PBS with 0.04% BSA). Viable singlets were sorted based on scatter properties and DAPI exclusion. Approximately 3000 cells were then pelleted and resuspended in PBS for single-cell droplet-based capture using 10X Chromium instruments following the manufacturer’s protocol. Transcriptome libraries were prepared with steps including fragmentation, end-repair, A-tailing, and double-sided size selection, followed by adaptor ligation. Library sequencing was performed on Illumina NextSeq 550, and data were mapped (GRCh38) counted and furthered processed using Cell Ranger-v7.2 to generate raw gene expression matrices per sample.

For ST, 10× FFPE gene expression slides (PN-1000185, 10X Genomics) were utilized. 5-μm FFPE section slides were initially dewaxed using xylene and stained with hematoxylin and eosin. Following visualization and scanning of the entire slide, RNA was released through decrosslinking using tris-ethylenediaminetetraacetic acid (TE) buffer. Hybridization was conducted overnight using forward and reverse human transcriptome probes (PN-1000364, 10X Genomics). Subsequently, cDNA libraries were prepared according to the 10X Genomics protocol (CG000407_VisiumSpatialGeneExpression forFFPE_UserGuide_RevA). Sequencing was performed on NovaSeq 6000 (Illumina), and raw reads were processed using Space Ranger-v3.1, aligned to the human genome (GRCh38, ENSEMBL).

### Data collection and pre-processing from public databases

The spatial transcriptomics public dataset for a human colon cancer sample (H1-VM2JXXK)^88^ was downloaded from a spatial transcriptomics research website (10X Genomics^89^, https://www.10xgenomics.com/). Detailed scRNA-seq cell-type annotation files for two human CRC datasets, EMTAB8107^90^ (23,176 cells) and GSE166555^91^ (66,050 cells), were obtained from the Tumor Immune Single-cell Hub 2 (TISCH2) online platform^92^.

RNA-seq data and corresponding clinicopathological information for COAD and READ samples were sourced from The Cancer Genome Atlas (TCGA)^93^. Additionally, genome-wide expression data and clinicopathological information for three other CRC cohorts (GSE17538, GSE38832, GSE39582, all based on the Affymetrix® GPL570 platform) were retrieved from the Gene Expression Omnibus (GEO) database using the R package ‘GEOquery’^94^. Moreover, complete RNA-seq data and corresponding clinical characteristics for IMvigor210 cohort^95^ (anti-PD-L1 immunotherapy in bladder cancer), PRJEB23709 cohort^96^ (anti-PD-1 immunotherapy in melanoma), Schadendorf cohort^97^ (anti-PD-1 and anti-CTLA-4 immunotherapy in melanoma) and the Riaz cohort^59^ (anti-PD-1 and anti-CTLA-4 in melanoma) were obtained from their respective sources. RNA-seq fragments per kilobase per million transcripts (FPKM) from the TCGA database were converted to transcripts per kilobase million (TPM) and subsequently log2 transformed. RNA-seq read counts from the IMvigor201 cohort underwent conversion to TPM and log2 transformation. TCGA-CRC cohort was combined from TCGA-COAD and TCGA-READ datasets, and a meta-cohort was created by combining TCGA-CRC cohort with all GEO datasets after the removal batch effects using the ComBat algorithm.

### Selection of CAF markers

To identify reliable CAF markers, we conducted a comprehensive literature review of studies published since 2010. To ensure scientific rigor, only publications from Q1-ranked journals (based on JCR classification) were considered. A gene was included as a CAF marker only if (1) it was reported in at least two independent review articles as a CAF marker and (2) at least one original research article provided supporting protein-level or spatial localization evidence (e.g., IHC, IF, ISH, or spatial transcriptomics). A detailed list of these markers and their corresponding references is provided in Table S1.

### Identification of CAF feature genes in CRC

Re-analysis and differential analysis of scRNA-seq data between CAFs and other cell types were carried out using the TISCH2 platform. Analysis and validation of the corresponding gene sets in the spatial transcriptome datasets were conducted using Loupe Browser (8.0.0). Genes displaying significant differential expression in fibroblasts and myofibroblasts were identified as CAF feature genes (adjusted p-value < 0.05, logFC > 1). Molecular pathways related to these CAF feature genes were assessed with the ‘clusterProfiler’ package in R for Gene Ontology (GO) and Kyoto Encyclopedia of Genes and Genomes (KEGG) analysis.

### Signature derived through integrated machine learning approaches

For the development of the CRPS with high accuracy and stability, integration of ten machine learning algorithms and exploration of 101 algorithm combinations were undertaken. The machine learning techniques encompassed various methodologies, including Elastic network (Enet), Lasso, Ridge regression, stepwise Cox regression, CoxBoost, random survival forest (RSF), supervised principal components (SuperPC), partial least squares regression for Cox (plsRcox), generalized boosted regression modeling (GBM), and survival support vector machine (survival-SVM).

The procedure for generating the signature proceeded as follows: (a) Identification of prognostic-related CAF feature genes in TCGA-CRC cohort was conducted using the log-rank test; (b) Subsequently, 101 algorithm combinations were applied to these prognostic CAF feature genes to construct prediction models based on the leave-one-out cross-validation (LOOCV) framework within TCGA-CRC cohort; (c) Evaluation of all models was performed across the training dataset and four validation datasets (GSE17538, GSE38832, GSE39582, and meta-cohort); (d) Calculation of Harrell’s concordance index (C-index) across all validation datasets enabled selection of the model with the highest average C-index as optimal. The risk score of the optimal model (RSF and Enet (α = 0.1)) was determined using regression coefficients (coef), the formula is:

$$\begin{array}{lll}{\rm{CRPS}}\,{\rm{Risk}}\, {\rm{Score}}=&&\left(-0.09176987\times {\rm{PDGFRA}}\,{\rm{mRNA}}\,{\rm{expression}}\right)+\left(0.08774070\times {\rm{HSPB}}1\,{\rm{mRNA}}\,{\rm{expression}}\right)\\&&+\left(0.13331131\times {\rm{TPM}}2\,{\rm{mRNA}}\,{\rm{expression}}\right)+\left(0.11778132\,\times {\rm{BCAM}}\,{\rm{mRNA}}\,{\rm{expression}}\right)\\ &&+\left(-0.05975121\times {\rm{TPM}}1\,{\rm{mRNA}}\,{\rm{expression}}\right)+\left(0.06276078\,\times {\rm{CLU}}\,{\rm{mRNA}}\,{\rm{expression}}\right)\\&&+\left(-0.09880238\times {\rm{CCL}}11\,{\rm{mRNA}}\,{\rm{expression}}\right)\end{array}$$

Furthermore, survival modeling and Kaplan-Meier analysis for CRPS across all datasets were performed with the ‘survival’ and ‘survminer’ packages in R.

### Predictive performance analysis and peer comparison for CRPS model

The predictive performance of the CRPS was assessed using time-dependent ROC analysis and their AUC values, utilizing the ‘survivalROC’ package in R.

We collected 58 publicly available CRC signatures, each comprising genes and their corresponding coefficients (Table S4). For any coefficients that were not reported in the original studies, we estimated them using a multivariate Cox proportional hazards model by employing the calcoef function from the ‘survival’ package in R, which allowed us to derive coefficients based on the available mRNA expression data. Subsequently, univariate Cox regression analysis was applied to evaluate the predictive power of various publicly available CRC signatures across all datasets. The concordance index (C-index) for each signature was calculated to assess predictive accuracy, where the C-index is given by $C=\,\frac{{Number\; of\; concordant\; pairs}}{{Total\; number\; of\; comparable\; pairs}}$, The standard error (SE) of the C-index was estimated as ${SE}=\,\sqrt{\frac{C(1-C)}{n\times (1-\bar{C})}}$ *(Where C is the computed C-index, n is the number of events*, $\bar{C}$*is the expected value of the C-index.)*, and the 95% confidence intervals were computed using *Lower = C-*1.96 × *SE* and *Upper = C +* 1.96 × *SE*. The C-indexes of different signatures were compared using the ‘CompareC’ package in R.

### Nomogram construction

To determine if the CRPS risk score could serve as an independent prognostic factor, Kaplan-Meier analysis of CRPS within sub-groups of TCGA-CRC cohort was performed using the previously described methods. Both univariate and multivariable prognostic analyses were conducted in TCGA-CRC cohort. A nomogram was developed for TCGA-CRC cohort to predict the survival probabilities of CRC patients at 1, 3, and 5 years, and the corresponding calibration curves were plotted based on multivariable Cox regression analysis using the ‘rms’ package in R. Time-dependent ROC analysis and the corresponding AUC values for the nomogram were generated as previously described.

### Clinical and molecular significance of the CRPS

Based on the median CRPS risk score, CRC patients in TCGA-CRC cohort were classified into high and low risk groups. Differences in clinicopathological features between two risk groups were compared across both testing and validation datasets and visualized using the ‘ggplot2’ package in R. Consensus Molecular Subtypes (CMS) for TCGA-CRC cohort were determined using the R package ‘CMScaller’^98^.

DEGs between the two risk groups (adjusted p-value < 0.05, logFC > 1, screened using ‘limma’ package in R) were used for GO and KEGG analysis, with methods previously detailed. Additionally, the GSEA software^99^ was employed to analyze significantly enriched pathways of these DEGs. The biological functions of every tumor sample were also quantified using the ‘GSVA’ package in R^100^, the differential analysis of GSVA pathway scores between high- and low-risk groups was performed using the ‘limma’ package in R. Specific pathway signatures including Hallmark, C2 and C5 gene sets were downloaded from in the MSigDB database^101^. Furthermore, ssGSEA pathway scores for other gene features related to cellular activities were calculated using the GSVA package through the ssGSEA algorithm^102^.

### Evaluation of gene somatic mutations

Somatic mutation and copy number variation data for TCGA-CRC cohort were retrieved from the Genomic Data Commons (GDC)^103^. To analyze and visualize the MAF files of somatic mutation data for the two risk groups, and to calculate the TMB score for individuals in TCGA-CRC cohort, the ‘maftools’ R package^104^ was employed. MSI data for TCGA-CRC cohort were accessed via Firebrowse database^105^.

### TME immunological characteristics analysis

The immune cell infiltration for CRC individuals in TCGA-CRC cohort was estimated using methods including MCPCOUNTER^106^, XCELL^107^, and TIMER^108^. The immune, stromal, and ESTIMATE scores, as well as tumor purity scores, were calculated with the ‘estimate’ package in R^109^. Immuno-modulators, including major histocompatibility complex (MHC) molecules, immune-stimulators, immune-stimulatory receptors, immune-inhibitors, and immune-inhibitory receptor markers, were collected from a previous study^110^. All findings were visualized using stacked graphs, heat maps, violin plots, and box plots with the ‘ggplot2’ package in R.

HE staining immunophenotype pathology images (FFPE) of TCGA-CRC cohort were obtained from the Cancer Digital Slide Archive (CDSA)^111^. Data on the application of deep learning to identify tumor-infiltrating lymphocytes from HE pathological images of TCGA-CRC cohort were derived from the study by Joel et al.^21^.

### Prediction of immuno-therapeutic response

Immunotherapy sensitivity scores for predicting responses to CTLA-4 and PD-1 inhibitors in TCGA-CRC cohort were obtained from the Cancer Immunome Database (TCIA)^110^. The SubMap algorithm^112^ was utilized to estimate the potential response of TCGA-CRC samples to immunotherapy. Based on the subtype annotations and correlated bulk RNA-seq data^59^, predictions and comparisons of responses to anti-PD-1 and anti-CTLA-4 immunotherapy in high- and low-risk samples were made.

Additionally, to explore the predictive value of CRPS for anti-PD-L1, anti-PD1, and anti-CTLA4 therapies, the CRPS risk model was applied to the IMvigor210, PRJEB23709, and Schadendorf cohorts. Patients exhibiting stable disease (SD) or progressive disease (PD) were categorized as non-responders, whereas those showing complete response (CR) or partial response (PR) were categorized as responders.

### Construction of CRPS phenotypes

To identify CRPS phenotypes in CRC, unsupervised clustering analysis was performed based on the expression levels of 7 CRPS-related genes. The optimal clustering numbers and phenotypes of CRC individuals in TCGA-CRC cohort were determined using the consensus clustering algorithm, via the ‘ConsensusClusterPlus’ package in R^113^.

The statistical differences in overall survival between different phenotypes were calculated and visualized using methods outlined earlier. The relationships between CRPS phenotypes and two risk groups, as well as comparisons of TMB, immune infiltration levels, and immunotherapy outcomes between the two phenotypes, were visualized with sankey diagram, heatmap, violin plot, box plots, and bar plots using the ‘ggplot2’ package in R. GSVA analysis was performed and differences between the molecular functions of two phenotypes were compared using the previously described methods.

### Prediction of potential drugs and selection of key gene

Expression profile data of CCLs were downloaded from the Broad Institute Cancer Cell Line Encyclopedia (CCLE) project^114^. Drug sensitivity data for these CCLs were obtained from the Cancer Therapeutics Response Portal (CTRP)^115^ and the PRISM Repurposing dataset^116^, encompassing sensitivity data for 355 compounds in CTRP and 1286 compounds in PRISM. Drug sensitivity was quantified by the area under the dose–response curve (AUC), with lower AUC values indicating increased drug sensitivity. To enhance the accuracy of subsequent drug response predictions by removing confounding transcriptional signals, the ISOpure algorithm was utilized to eliminate non-tumor components, yielding a purified tumor expression matrix for TCGA-CRC cohort using non-tumor expression profiles as references. The ‘pRRophetic’ package in R^117^, with its built-in ridge regression model, was then employed to predict drug responses for clinical samples based on their purified expression profiles, resulting in estimated AUC values for each compound in each TCGA sample.

For drug sensitivity analysis, the estimated AUC values and estimated drug IC50 values for cancer cell lines were obtained from the Genomics of Drug Sensitivity in Cancer (GDSC)^118^. Using the ‘lolR’ package in R, a nearest centroid classifier was developed to predict the risk group classification of CRC cell lines based on the expression of essential genes from TCGA-CRC. Before comparing drug sensitivity, missing AUC values were addressed using K-nearest neighbor (k-NN) imputation, with compounds having over 10% missing data being excluded prior to the analysis.

The intersections of potential drug candidates, comparisons of drug sensitivity between high-risk and low-risk groups, and the relationship between gene expression levels and CRPS risk scores were illustrated using Venn diagrams, box plots, bar plots, and volcano plots. All visualizations were created with the ‘ggplot2’ package in R.

### scRNA-seq analysis

For scRNA-seq data, all samples from multiple patients were pooled for integrative multimodal analysis using the R package ‘Seurat’^119^. Genes detected in fewer than 3 cells, as well as cells expressing fewer than 200 or more than 6,000 genes, were filtered out. Additionally, cells with mitochondria gene proportions exceeding 25% were excluded. Cell cycle scoring was performed for S phase and G2M phase, and predicted cell cycle phases were calculated. Ultimately, 50,831 cells remained for downstream analysis.

To classify single cells into distinct subsets, we followed these steps: the selection of variable genes using variance stabilizing transformation (VST). We mitigated batch effects among samples using the R package ‘harmony’^120^, reduced dimensionality, and projected cells onto graphs^121^. Principal component analysis (PCA) was conducted on scaled data of highly variable genes. The first 30 principal components (PCs) were used for clustering cells and performing subtype analysis via nonlinear dimensionality reduction (t-SNE). Cell clusters were identified at optimal resolution using a shared nearest neighbor (SNN) modularity optimization-based clustering method. We employed the ‘FindClusters’ function of Seurat, which calculates k-NN and constructs the SNN graph. The original Louvain algorithm (algorithm = 1) was applied for modularity optimization.

The ‘fibroblasts’ cell type in CRC tissues underwent re-clustering and re-analysis at a higher resolution. The ‘FindAllMarkers’ function was used to identify marker genes for each cluster, with an absolute logFC > 1 and a minimum cell population fraction of 0.25 in each population (Table S7). Subsequently, fibroblasts were further divided into high/low *HSPB1* expression groups based on the median of *HSPB1* expression. Expression patterns of various marker genes and *HSPB1* across all clusters and subclusters were visualized using the ‘FeaturePlot’, ‘DotPlot’, and ‘VlnPlot’ functions in Seurat. DEGs between high/low *HSPB1* expression groups were used for GO and KEGG analysis, with methods previously detailed.

NFs were merged with the CAFs cell groups for trajectory analysis. By applying the Monocle algorithm^122^, we employed the NewCellDataSet function to create a new object using the transcript count data from fibroblasts. The results obtained from the estimateSizeFactors and estimateDispersions functions facilitated normalization of mRNA recovery differences across cells and supported subsequent differential expression analysis. Signature genes (expressed in at least 10% of the cells in the dataset) were selected based on the top 2,000 q-values calculated using the differentialGeneTest function to delineate the trajectory progress. The ReduceDimension function was then applied to condense the data into two dimensions, followed by the orderCells function, which arranged the cells according to their gene expression profiles. The plot_cell_trajectory function was used to visualize the shift in fibroblasts subtypes, cell states and expression of *HSPB1* with pseudo-time. Cell–cell communication networks were predicted from scRNA-seq data using the ‘CellChat’ package in R^123^. The analysis was based on the Secreted Signaling ligand–receptor interaction database in CellChatDB.human. The netVisual_circle function was used to depict the number and strength of interactions among cell subtypes, netVisual_bubble to display upregulated ligand–receptor pairs and their signaling probabilities, and netAnalysis_signalingRole_heatmap to visualize the relative importance of each cell type as a sender, receiver, mediator, or influencer in the predicted signaling network.

### ST analysis

For ST data, the ‘Seurat’ package in R was employed to process the Space Ranger output files. Data normalization was conducted using SCTransform, followed by scaling of the data using ScaleData. PCA was then applied for dimensionality reduction. Subsequently, the scaled matrix was utilized to compute the mean values of multiple gene signatures, including well-established fibroblast markers, CAF markers, tumor markers, CRPS, and the expression level of *HSPB1* at each spatial spot. Finally, the ‘log1p’ function was applied to transform the mean values of these gene signatures into corresponding scores. The resulting scores were visualized using the function ‘SpatialFeaturePlot’.

### Fibroblasts isolation and HSPB1 overexpression

CAFs were isolated from human CRC tissues (Human colon cancer fibroblasts, HCCF) following a specified protocol^124^, with minor modifications. Briefly, tissue samples were minced and digested with collagenase. The resulting fragments were seeded in culture dishes with DMEM supplemented with 10% FBS. After 7–10 days, fibroblasts began to emerge from the tissue and proliferate in the culture medium. The plasmid vector containing *HSBP1* (PGMLV-CMV-H_HSPB1-3×Flag-PGK-Puro) was created by Jiman Biotechnology Co., Ltd. (Shanghai, China). For transient transfection, cells were plated in a 6-well culture dish 24 hours prior to transfection. The cells were then transfected with the appropriate vector using Lipofectamine 3000 (Invitrogen, Carlsbad, CA), following the manufacturer’s instructions.

### RNA extraction, reverse transcription PCR and quantitative real-time PCR

Total RNA was extracted using TRIzol reagent (Vazyme, China) following the manufacturer’s instructions. First-strand cDNA was synthesized using a Reverse Transcriptase kit (Vazyme, China). Quantitative Real-Time PCR (RT-qPCR) was performed using the SYBR Green method (Applied Biosystems, USA) on the 7900 Real-Time PCR System with the SDS 2.4 software sequence detection system (Applied Biosystems, USA). β-actin was used as an internal control to quantify mRNA levels. The relative expression levels of RNA were calculated using the 2 − ΔΔCT method.

### Western bolt

Western blot analysis was performed according to previously described protocols^125^. Information on the primary antibodies is provided in Table S6.

### CRC cell lines and cell culture

The human CRC cell line HCT116 was obtained from American Type Culture Collection (ATCC, USA). Cells were cultured in RPMI‑1640 medium (Gibco, Carlsbad, CA, USA) supplemented with 10% fetal bovine serum (FBS; Sigma, St. Louis, MO, USA), 100 U/ml penicillin, and 100 μg/ml streptomycin (NCM Biotech, Suzhou, China). Cultures were maintained at 37 °C in a humidified incubator containing 5% CO₂. The culture medium was replaced every 2–3 days, and cells were passaged at approximately 80–90% confluence using 0.25% trypsin‑EDTA (Gibco, USA). Only cells within 10 passages were utilized for experiments to ensure consistency and phenotypic stability.

### Conditioned medium (CM) preparation

For the preparation of HCCF-conditioned medium (HCCF-CM), approximately 1 × 10⁷ HSPB1-NC/OE HCCFs were cultured in complete medium until they reached about 80% confluence. The culture medium was then replaced with serum-free medium, and the cells were incubated for 48 h at 37 °C in a humidified incubator with 5% CO₂. Following incubation, the supernatant was collected and centrifuged at 2000 rpm for 5 min at 4 °C to remove cell debris. The resulting supernatant was then passed through a 0.22 μm filter to ensure sterility. CM was either used immediately for subsequent assays or stored at 4 °C for no longer than 3 days before use.

### Transwell migration assay

Cell migration ability was assessed using Corning transwell insert chambers (8μm pore size; Corning). A chemoattractant (600 μl of medium containing 10% FBS) was added to the lower well of each chamber. Approximately 1.5 × 10^4 cells were seeded into each chamber and incubated for 20–22 h at 37 °C.

### CCK-8 cell proliferation assay

For the CCK-8 assay, cells were seeded in 96-well plates (3 × 10^3 cells per well) and incubated lasting 3 days at 37 °C. The changes of cell proliferation were monitored daily using CCK-8 reagent (CCK8, APExBIO, USA), and the absorbance values were measured at 450 nm using a Hybrid Reader (BioTek, Winooski, USA).

### Colony formation assay

For the colony formation assay, HCT-116 cells were seeded in 6-well plates at a density of 1000 cells per well and cultured in HCCF-CM for approximately 10–14 days at 37 °C with 5% CO₂. After visible colonies formed, the medium was discarded, and the cells were gently washed twice with PBS. Colonies were fixed with 4% paraformaldehyde for 15 min at room temperature and stained with 0.1% crystal violet for 20 min. The stained colonies were washed with distilled water, air-dried, and photographed.

### Wound healing assay

HCT-116 cells were seeded into 6-well plates at a density of 5 × 10⁵ cells per well and cultured until they reached approximately 90% confluence. A straight scratch was created across the cell monolayer using a sterile 200 μL pipette tip. Detached cells were gently removed by washing twice with PBS, after which the cells were incubated with HCCF-CM. The scratch areas were photographed at 0 h and 48 h under an inverted microscope. The degree of wound closure was quantified by measuring the gap area using ImageJ software.

### Subcutaneous Xenograft Model in Nude Mice

BALB/c nude mice (4–6 weeks old, 18–22 g) were purchased from Shanghai Model Organisms Center Inc. (Shanghai, China). For the establishment of subcutaneous xenograft models, HCT116 cells and HCCF cells at a ratio of 3:1 (totaling 5 × 10⁶ cells suspended in 0.1 mL PBS) was injected subcutaneously into the right axillary region of each mouse using a 27‑gauge needle. Tumor growth was monitored every three days by measuring the length and width of each tumor with a vernier caliper. After 20 days of treatment, mice were euthanized, and tumors were excised and weighed. The collected tumor tissues were subsequently fixed in 4% paraformaldehyde for subsequent analyses.

### The immunohistochemistry (IHC)

Paraffin-embedded tissue slides were deparaffinized at 60 °C, followed by treatment with xylene and a graded alcohol series. Endogenous peroxidase activity was blocked by incubation in a 3% hydrogen peroxide solution for 15 minutes. After antigen retrieval, the slides were rinsed and incubated with 5% BSA to block nonspecific staining. The primary antibody of *Hsp27* (abcam, ab5579, 4 µg/ml) was incubated overnight at 4 °C in a humid chamber. The slides were visualized using the standard avidin-biotinylated peroxidase complex method. Finally, hematoxylin was used for counterstaining. The scoring criteria for IHC staining followed our previously established protocols, as detailed in an earlier report^125^.

### Multiplex immunofluorescence (mIF)

Paraffin-embedded tissue slides were deparaffinized at 60 °C, followed by treatment with xylene and a graded alcohol series. The endogenous peroxidase activity was blocked by incubation in a 3% hydrogen peroxide solution for 15 min. After antigen retrieval, the slides were rinsed and incubated with 5% BSA to block nonspecific staining. The primary antibody of *PANCK* (abcam, ab7753, 1 µg/ml), *Hsp27* (CST, #50353, 1:50), *FAPα* (abcam, ab207178, 1:50), and *Vimentin* (CST, #5741, 2 µg/ml) was sequentially incubated overnight at 4 °C in a humid chamber, followed by incubation with a fluorescent secondary antibody. The slides were then mounted and analyzed using microscopy.

### Statistical analysis

All data processing, statistical analyses, and plotting were performed using R 4.1.1 software. Spearman correlation analysis was employed to assess correlations between two continuous variables. The chi-squared test was used to compare categorical variables, while the Wilcoxon rank-sum test was applied to compare continuous variables. All statistical tests were two-sided, and a p-value < 0.05 was considered statistically significant.

## Supplementary information

Supplementary information

## Data Availability

Spatial transcriptome, single-cell sequence, and bulk RNA-seq data derived from this study will be deposited in NCBI SRA after all related projects are completed. However, these data can be made available from the corresponding author upon reasonable request. Key analysis code has been deposited at GitHub: [https://github.com/sj11788/CodeforCRPS/] (https:/github.com/sj11788/CodeforCRPS) and is publicly available. Any additional information or code required to reanalyze the data reported in this paper is available from the corresponding author upon reasonable request, in compliance with the law, due to human patient privacy concerns.
