# Supplementary material for: Development and validation of a CAF-related signature for prognosis and therapy response in colorectal cancer: new insights on HSPB1
Source: NPJ Precis Oncol. 2025 Dec 17;10:20. doi: 10.1038/s41698-025-01217-9 (PMC12800118; doi:10.1038/s41698-025-01217-9)
Supplement: Supplementary file 1 — Supplementary information [file 41698_2025_1217_MOESM1_ESM.pdf]

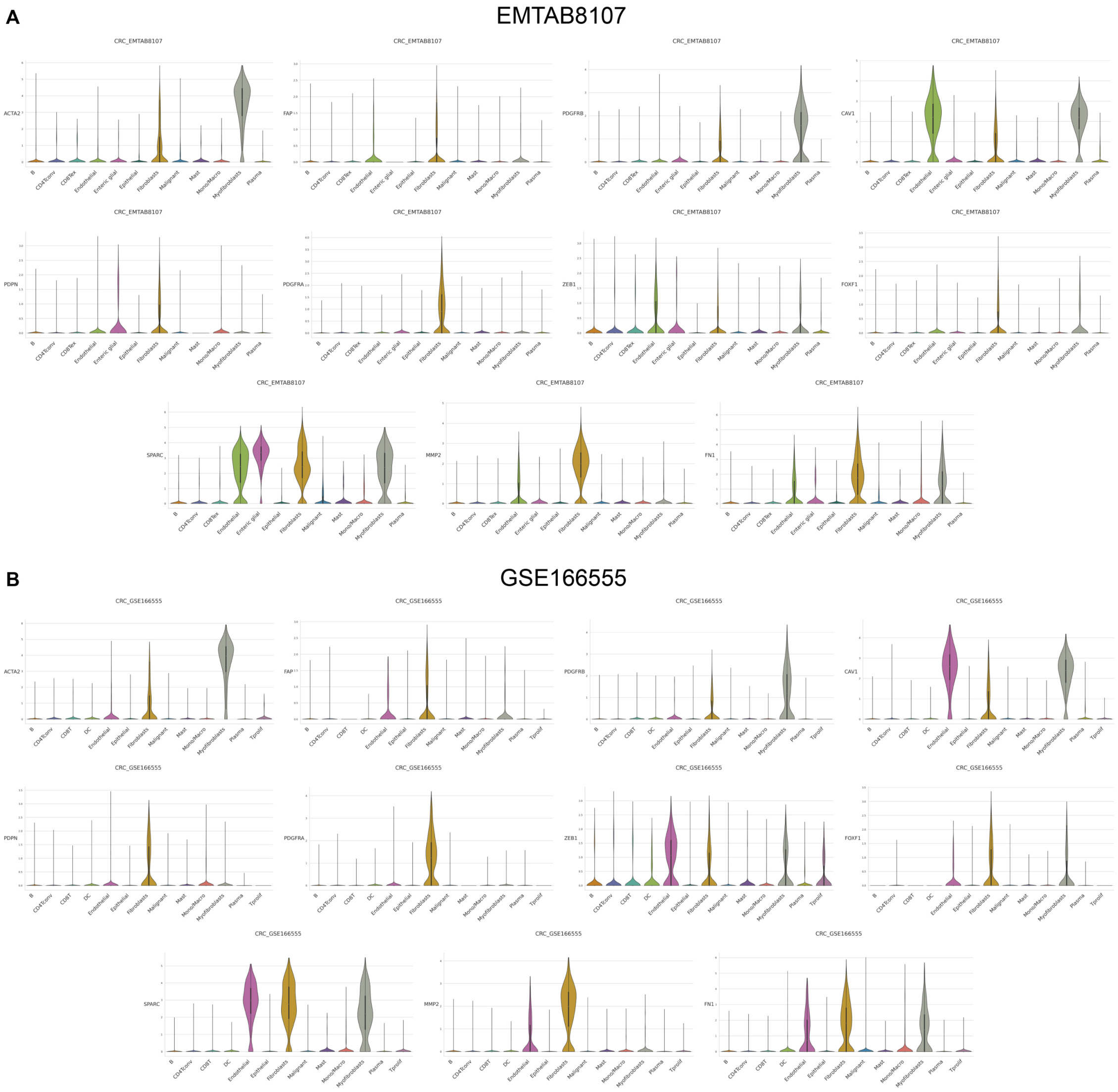


**Supplementary Figure 1. Expression patterns of eleven recognized CAF markers in public scRNA-seq datasets (related to Figure 1)**
(A-B) Violin plot showing expression patterns of eleven recognized CAF markers across different cell clusters in EMTAB8107 (A) and GSE166555 (B) dataset.

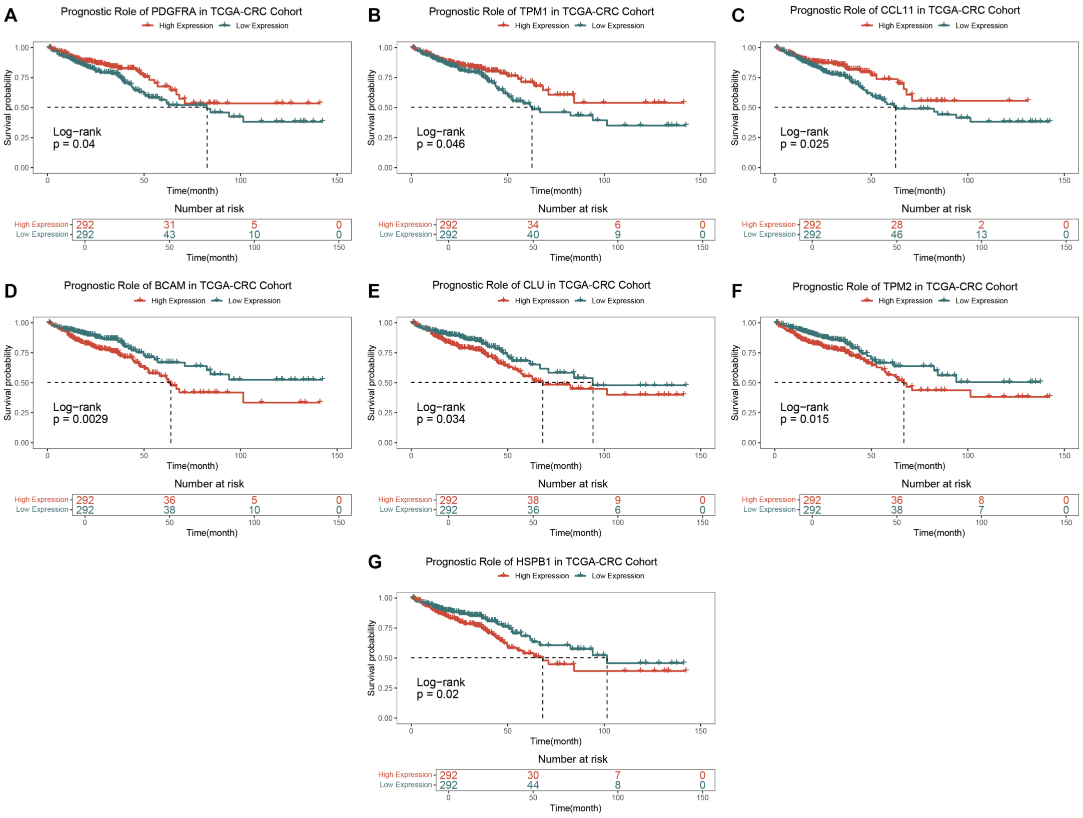


**Supplementary Figure 2. Prognostic roles of selected CRPS genes in the TCGA-CRC cohort (related to Figure 2)**

(A-G) Kaplan-Meier survival curves of CRPS genes regarding OS in TCGA-CRC cohort.

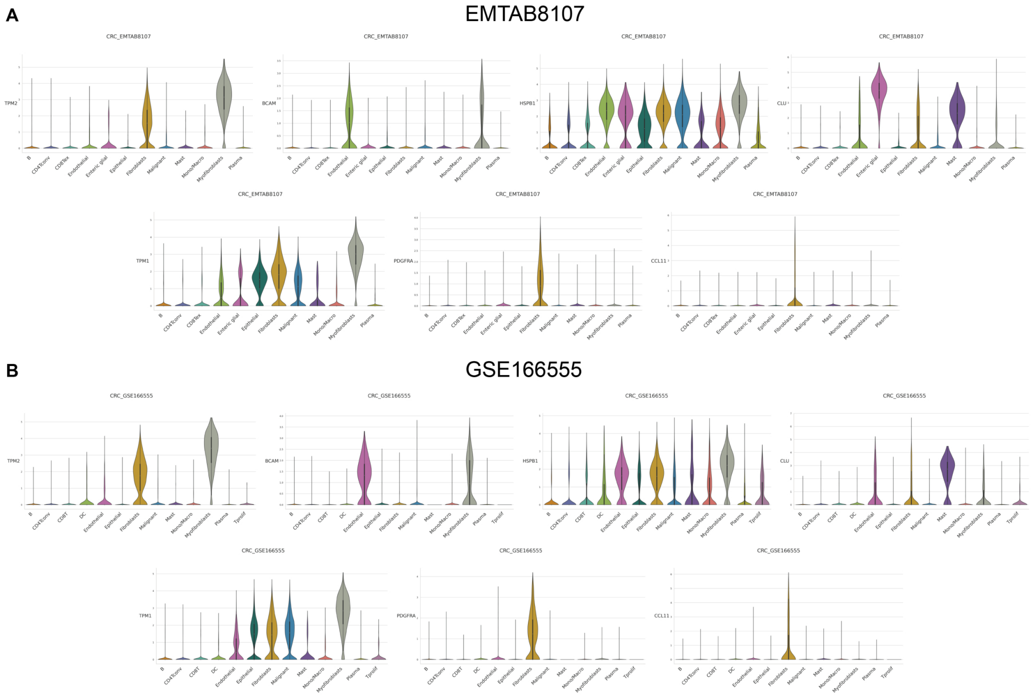


**Supplementary Figure 3. Expression patterns of CRPS genes in public scRNA-seq datasets (related to Figure 3)**

(A-B) Violin plot showing expression patterns of CRPS genes across different cell clusters in EMTAB8107 (A) and GSE166555 (B) dataset.

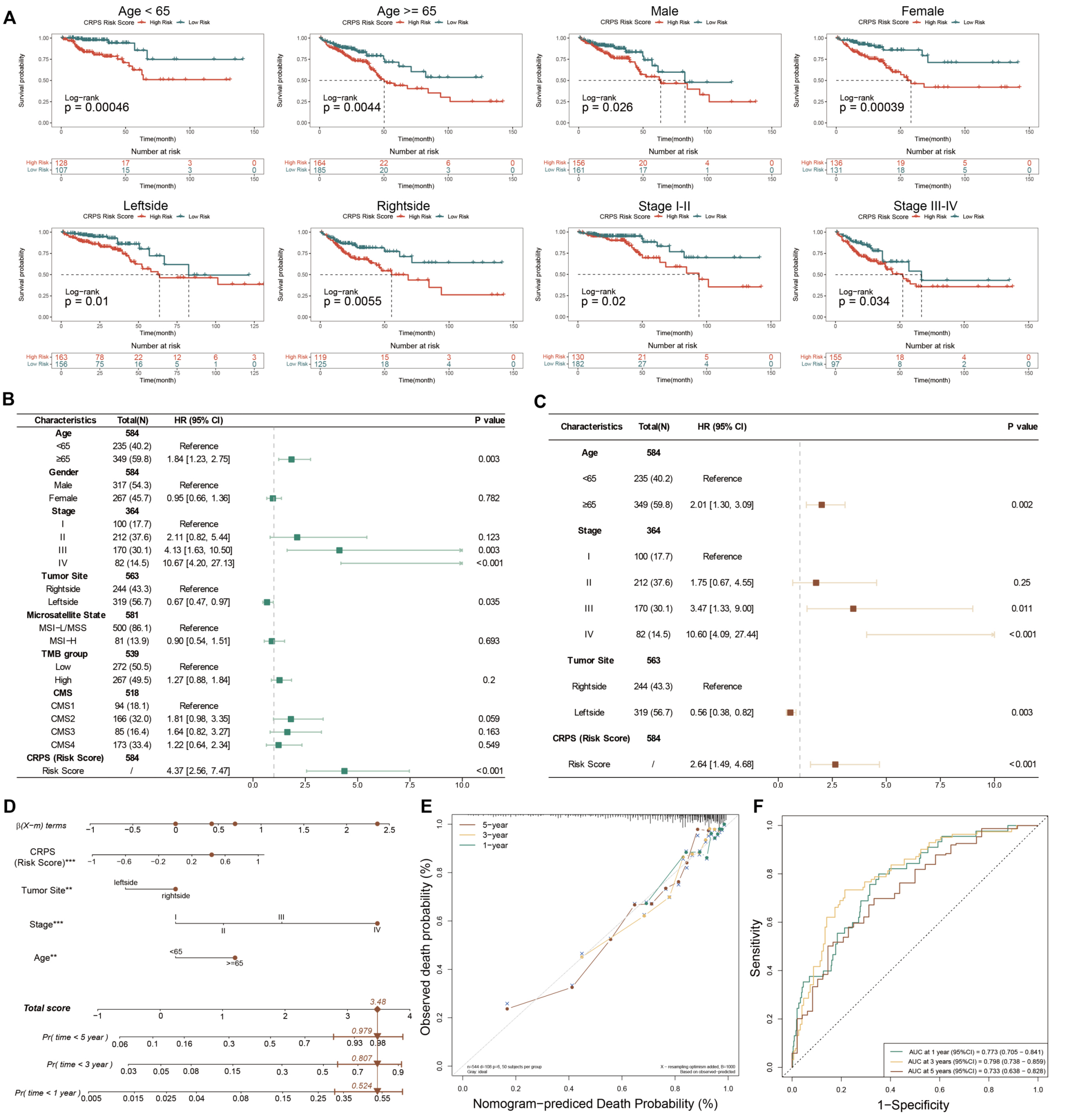


**Supplementary Figure 4. CRPS serves as an independent prognostic factor in constructing a nomogram with high predictive performance**

(A) Kaplan-Meier survival analysis of CRPS risk groups regarding OS within different subgroups of TCGA-CRC cohort.

(B-C) Results of the univariate (B) and multivariable (C) Cox regression analyses regarding OS in TCGA-CRC cohort.

(D) Nomogram for predicting 1, 3 and 5-year survival in TCGA-CRC cohort.

(E-F) Calibration curves (E) and time-dependent ROC analysis (F) for the nomogram predicting 1, 3, and 5-year survival in TCGA-CRC cohort.

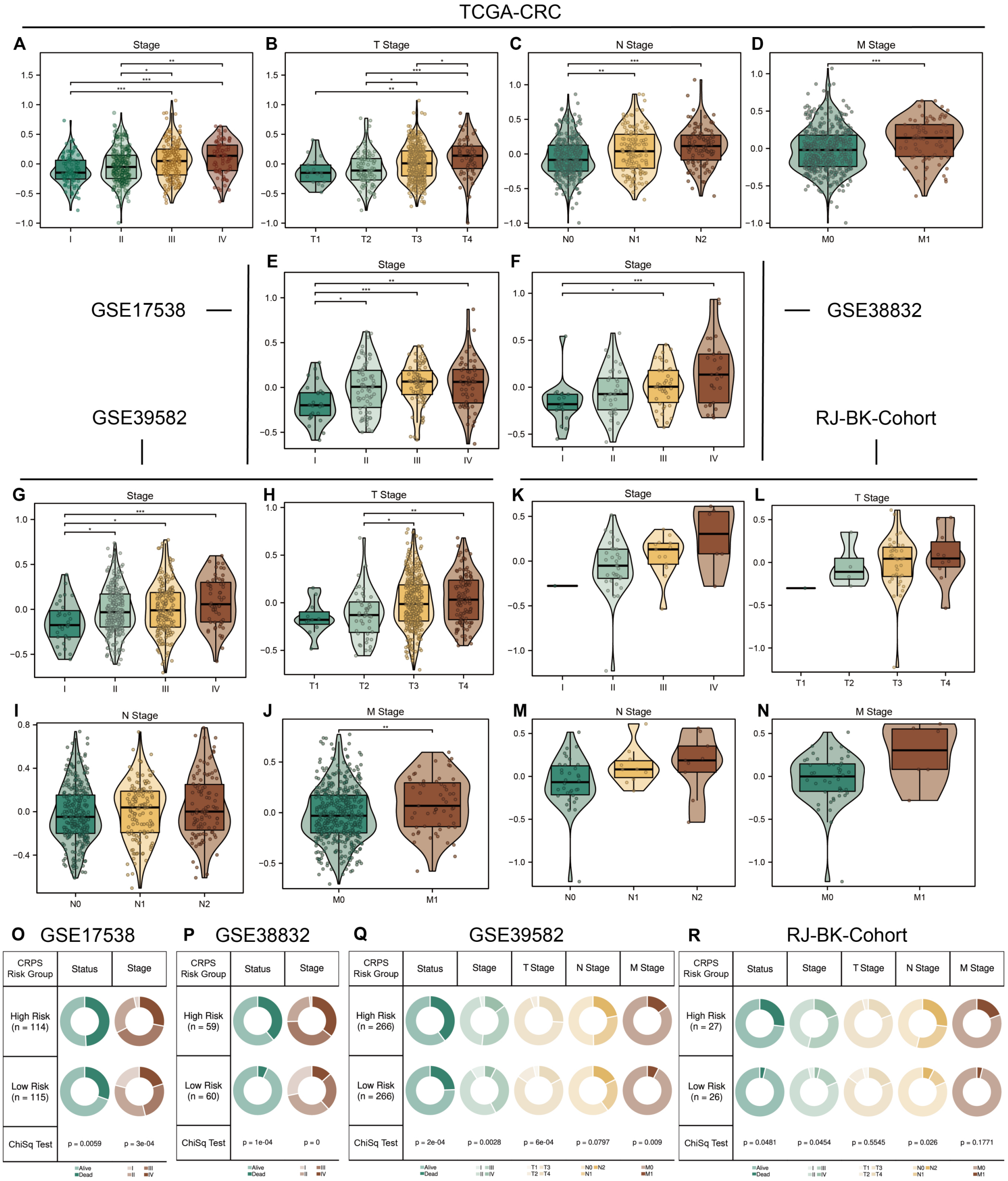


**Supplementary Figure 5. CRPS exhibit strong correlations with clinicopathological features in various cohort (related to Figure 4)**

(A-D) Distribution of CRPS risk score among tumor AJCC stage (A), T stage (B), N stage (C) and M stage (D) in TCGA-CRC cohort.

(E) Distribution of CRPS risk score among tumor AJCC stage in GSE17538 cohort.

(F) Distribution of CRPS risk score among tumor AJCC stage in GSE38832 cohort. Wilcoxon test.

(G-J) Distribution of CRPS risk score among tumor AJCC stage (G), T stage (H), N stage (I) and M stage (J) in GSE39582 cohort.

(K-N) Distribution of CRPS risk score among tumor AJCC stage (G), T stage (H), N stage (I) and M stage (J) in RJ-BK-cohort.

(O-R) Circos plot of different survival and pathological factors between two risk groups in GSE17538 cohort (O), GSE38832 cohort (P), GSE39582 cohort (Q) and RJ-BK-Cohort (R). Statistical significance was calculated using Wilcoxon test (A-N) and Chi-square test (O-R). * $P < 0.05$; ** $P < 0.01$; *** $P < 0.001$.

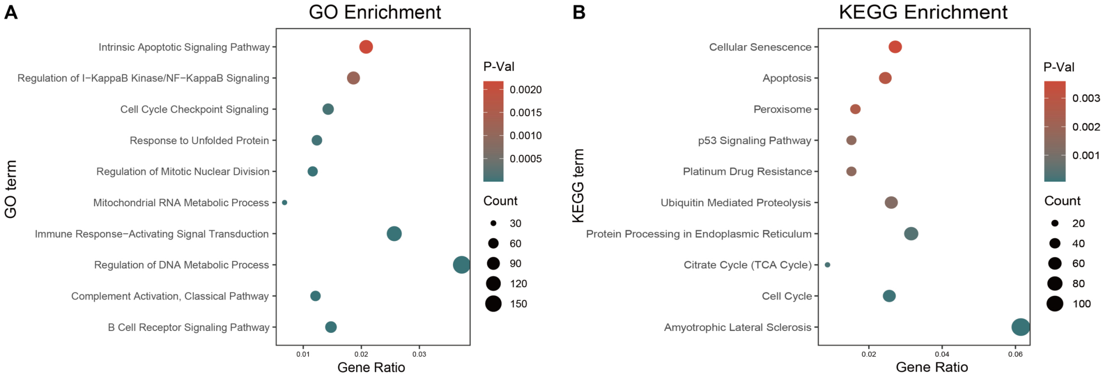


**Supplementary Figure 6. CRPS-related molecular mechanisms in TCGA-CRC cohort (related to Figure 4)**

(A-B) GO (A) and KEGG (B) analysis of DEGs between high and low CRPS risk groups.

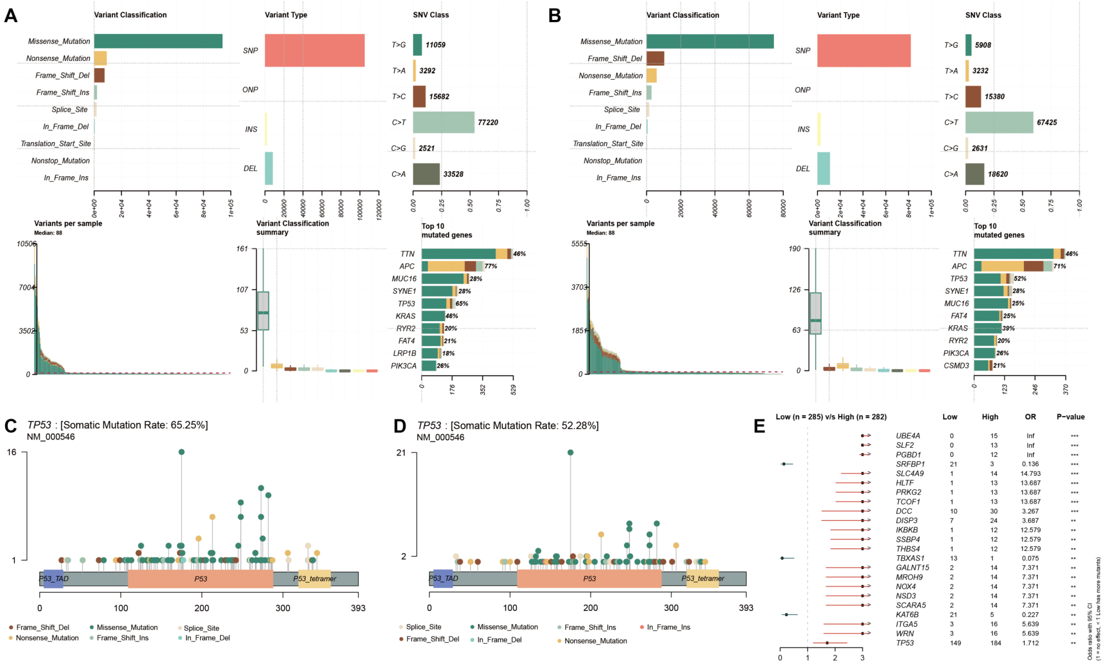


**Supplementary Figure 7. Notable distinctions in somatic mutations between high and low CRPS risk groups are observed in TCGA-CRC cohort (related to Figure 5)**

(A-B) MAF-summary plots of the somatic mutation in high (A) and low (B) CRPS risk groups of TCGA-CRC.

(C-D) Lollipop charts of the mutated TP53 gene in high (C) and low (D) risk groups.

(E) Forest plot showing variations in the somatic mutation frequencies between two risk groups. Statistical significance was calculated using Fisher's exact test (E). * $P < 0.05$; ** $P < 0.01$; *** $P < 0.001$.

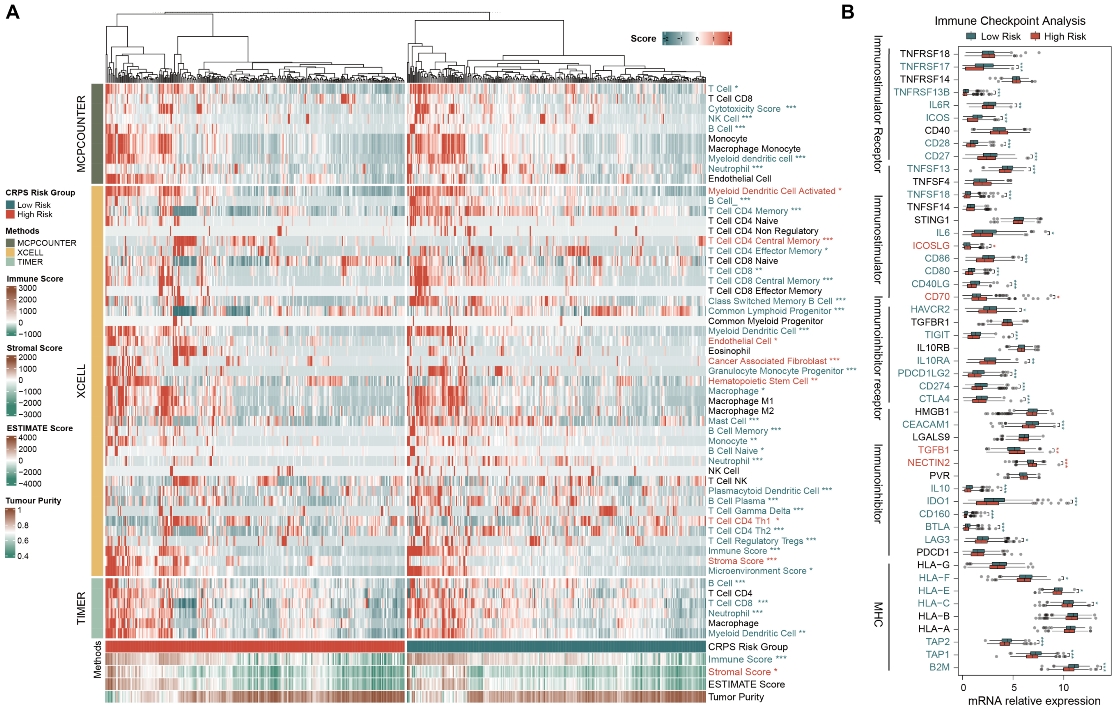


**Supplementary Figure 8. A strong association exists between CRPS and immune cell infiltration in TCGA-CRC cohort (related to Figure 5)**

(A) Thermogram displaying relationships between CRPS risk groups and tumor immune microenvironment using MCPCOUNTER, XCELL, TIMER and ESTIMATE analysis. Wilcoxon test.

(B) Boxplot showing the association between CRPS risk groups and mRNA expression of different kinds of immune checkpoint markers.

Statistical significance was calculated using Wilcoxon test (A-B). * $P < 0.05$; ** $P < 0.01$; *** $P < 0.001$.

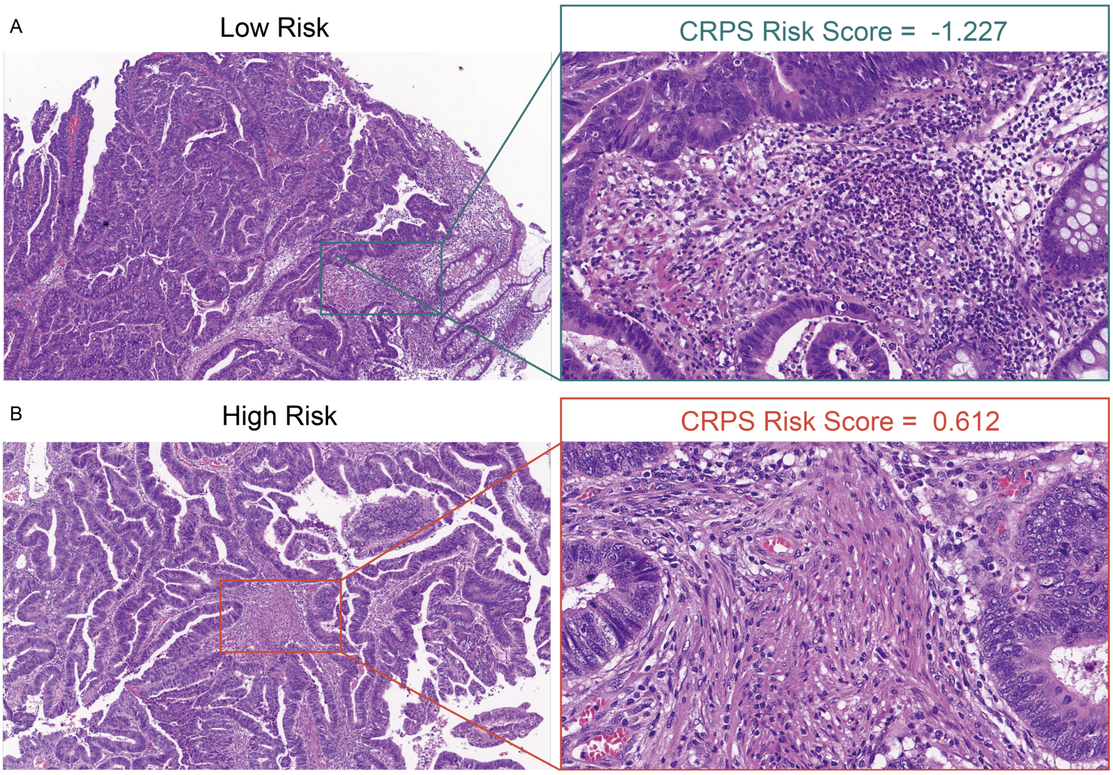


**Supplementary Figure 9. Distinct differences in pathological features are observed between high and low CRPS risk groups in the RJ-BK-Cohort (related to Figure 5)**

(A-B) Representative images showing pathological HE staining from low (A) and high (B) CRPS risk groups in RJ-BK-Cohort.

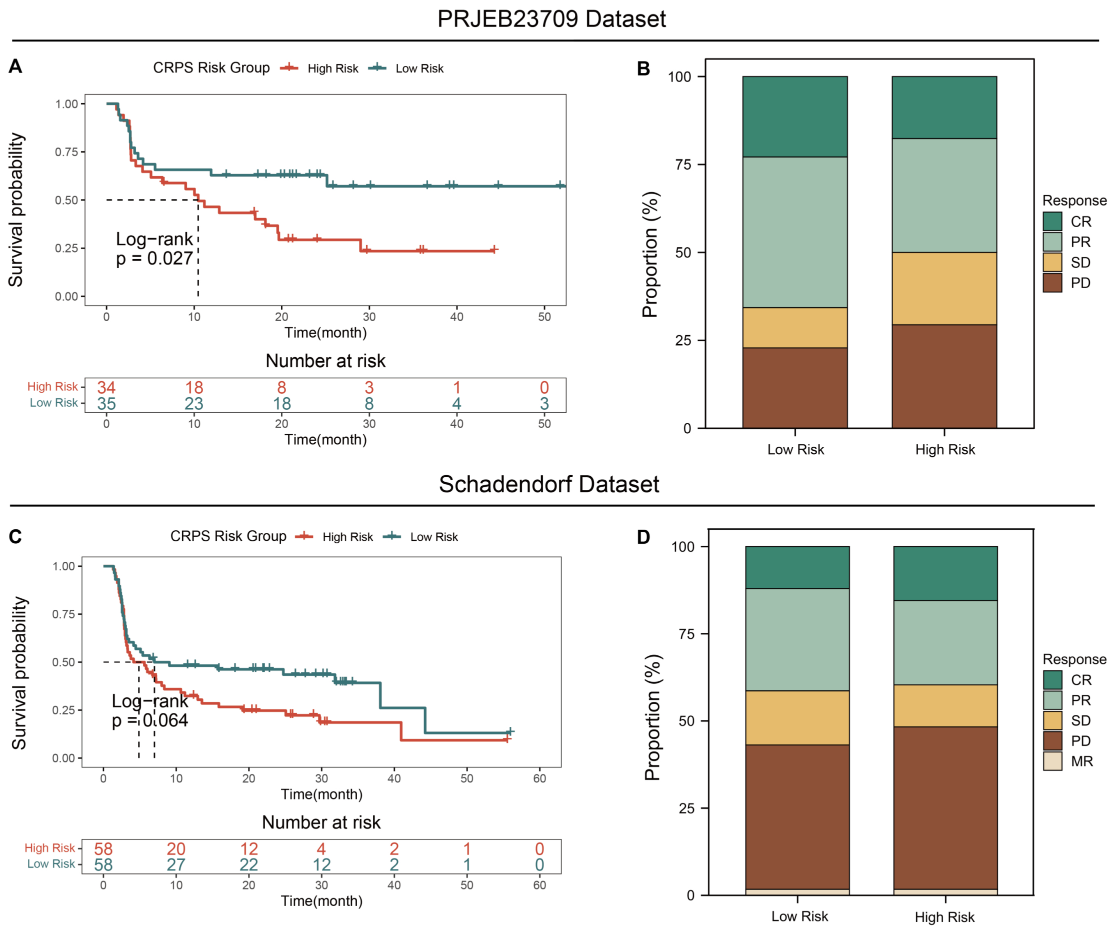


**Supplementary Figure 10. CRPS demonstrates excellent predictive capability for immunotherapy response in multiple cohorts (related to Figure 5)**

(A) Kaplan-Meier survival curves of CRPS risk groups regarding PFS in PRJEB23709 cohort.

(B) Stacked histogram showing variations in the proportion of different anti-PD-1 and anti-CTLA-4 responsiveness between two risk groups in PRJEB23709 cohort.

(C) Kaplan-Meier survival curves of CRPS risk groups regarding PFS in Schadendorf cohort.

(D) Stacked histogram showing variations in the proportion of different anti-PD-1 responsiveness between two risk groups in Schadendorf cohort.

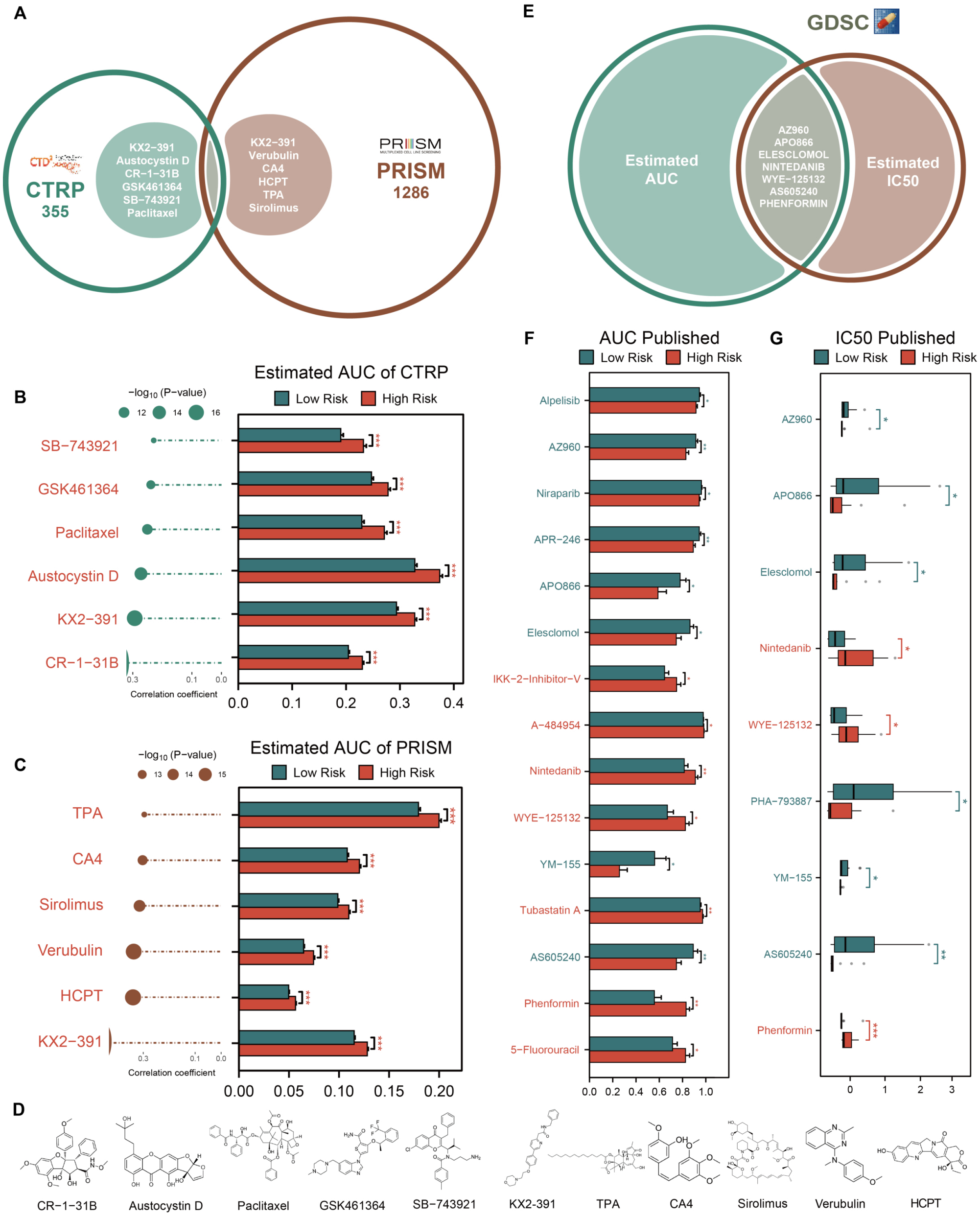


**Supplementary Figure 11. Drug sensitivity varies significantly between high and low CRPS groups**

**(A)** Venn diagram showing the compounds used for screening analysis from CTRP and PRISM.
**(B)** Correlation analysis and histogram displaying associations between CRPS and AUC values of six compounds from CTRP. Spearman correlation analysis.
**(C)** Correlation analysis and histogram displaying associations between CRPS and AUC values of six compounds from PRISM. Spearman correlation analysis.
**(D)** Structural formulas of potential therapeutic agents for low CRPS risk patients based on analyzed data from CTRP and PRISM.
**(E)** Venn diagram illustrating compounds with significant difference in both estimated AUC values and IC50 values from GDSC.
**(F)** Histogram showing variations in AUC values between high and low CRPS risk groups from GDSC.
**(G)** Boxplot showing variations in IC50 values between two risk groups from GDSC.
Statistical significance was calculated using Wilcoxon test (B-C and F-G). * $P < 0.05$; ** $P < 0.01$; *** $P < 0.001$.

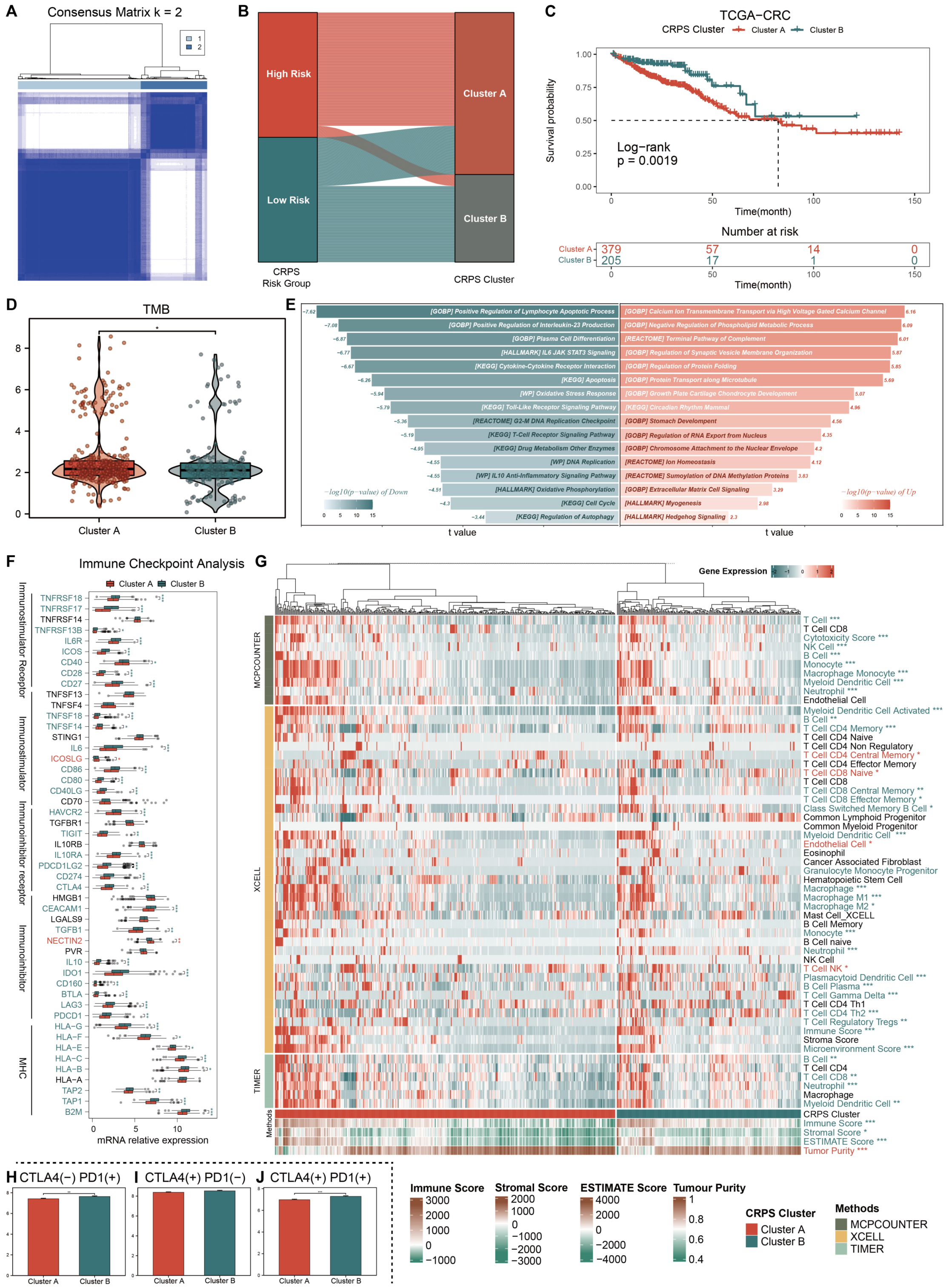


**Supplementary Figure 12. Identification of two CRPS-related molecular phenotypes by unsupervised learning**

(A) Consensus matrix thermogram defining two CRPS phenotypes (k = 2) in TCGA-CRC cohort and showing their correlation area by unsupervised learning.

(B) Sankey diagram displaying interrelationship between CRPS risk group and CRPS phenotypes.

(C) Kaplan-Meier survival curves of CRPS phenotypes regarding OS in TCGA-CRC cohort.

(D) Violin plot showing variations in TMB scores between CRPS phenotypes.

(E) Differences in MsigDB-based pathway activities between CRPS phenotypes scored by GSVA.

(F) Boxplot showing the association between CRPS phenotypes and mRNA expression of different kinds of immune checkpoints.

(G) Thermogram displaying relationships between CRPS phenotypes and tumor immune microenvironment using MCPCOUNTER, XCELL, TIMER and ESTIMATE analysis.
(H-J) Histograms showing variations in immunotherapy sensitivity scores between CRPS phenotypes in TCGA-CRC cohort.
Statistical significance was calculated using Wilcoxon test (D and F-J). * $P < 0.05$; ** $P < 0.01$; *** $P < 0.001$.

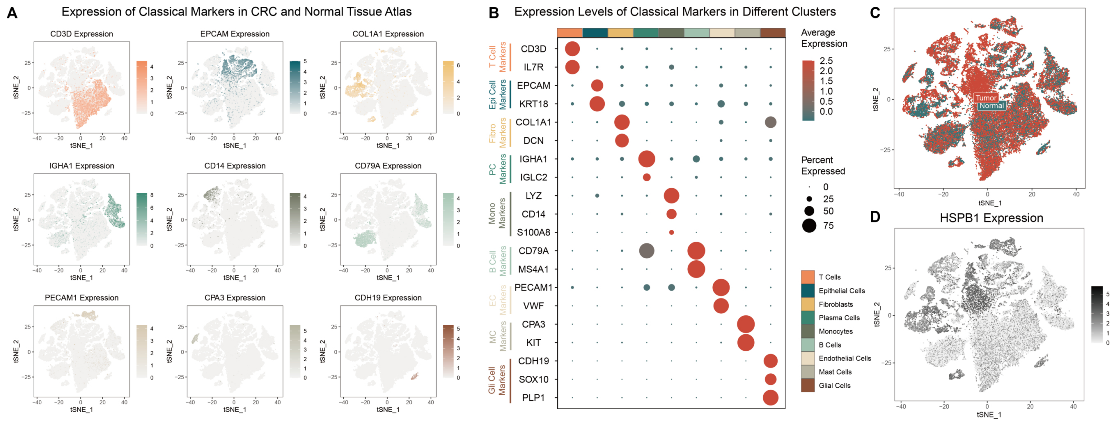


**Supplementary Figure 13. Expression of significant markers in CRC and normal tissue landscape (related to Figure 6)**

(A) t-SNE plot showing variable expression of recognized markers in different clusters.

(B) Dot plot showing expression of recognized markers in different clusters.

(C) t-SNE plot showing distinction of tumor and normal cells.

(D) t-SNE plot showing HSPB1 expression in different cell clusters.

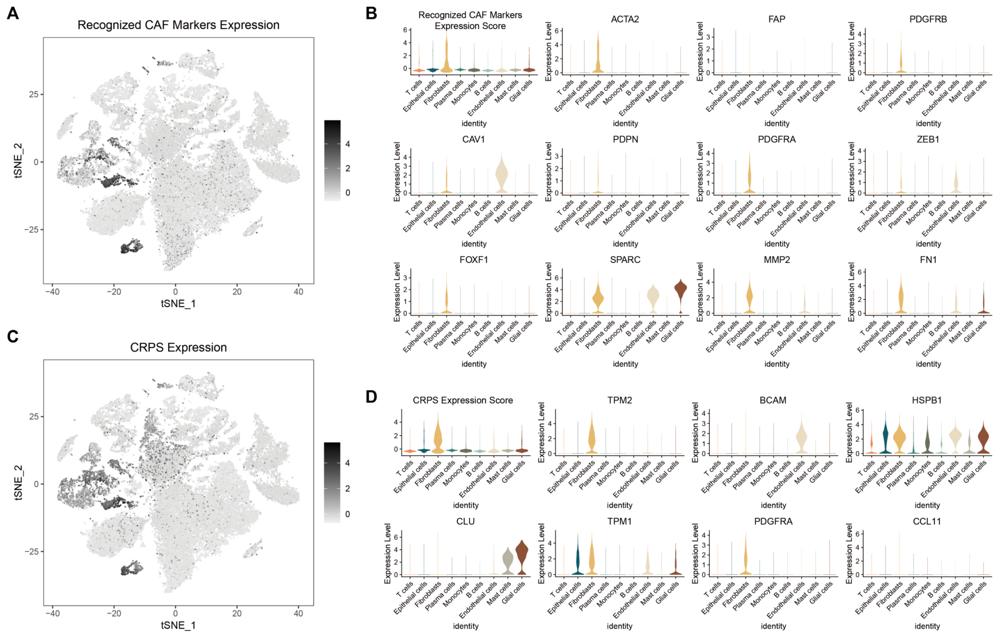


**Supplementary Figure 14. Expression patterns of eleven recognized CAF markers and CRPS in RJ-SC-Cohort (related to Figure 6)**

(A) t-SNE plot showing collective expression patterns of eleven recognized CAF markers across different cell clusters.

(B) Violin plot showing collective and individual expression patterns of eleven recognized CAF markers across different cell clusters.

(C) t-SNE plot showing collective expression patterns of CRPS genes across different cell clusters.

(D) Violin plot showing collective and individual expression patterns of CRPS genes across different cell clusters.

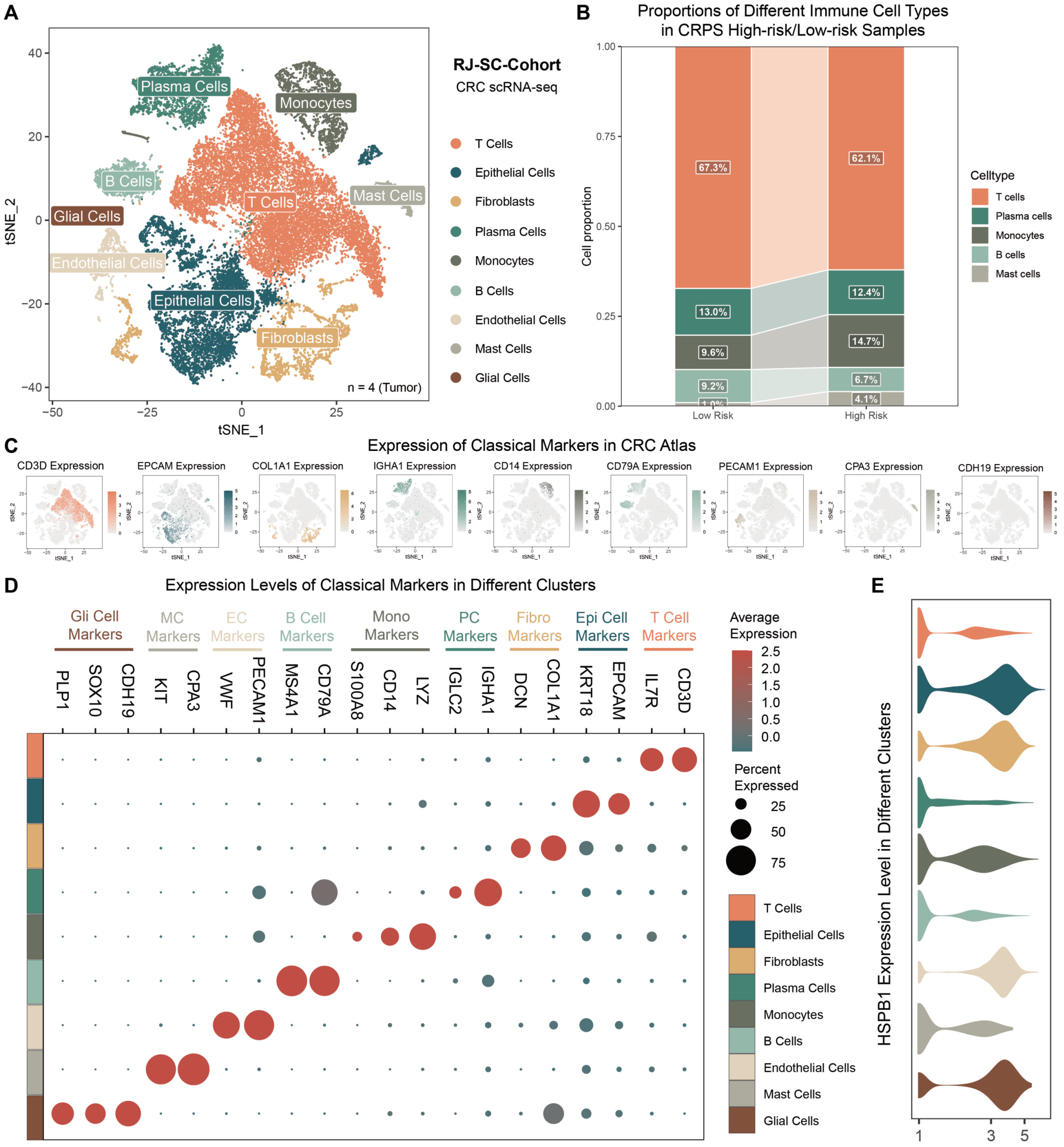


**Supplementary Figure 15. Single-cell analysis of CRC tissues (related to Figure 6)**

(A) t-SNE plot showing characterization of 30,804 cells profiled.

(B) Stacked histogram showing the relative abundances of different immune cell types in samples with high (n = 2) and low (n = 2) CRPS risk score.

(C) t-SNE plot showing variable expression of recognized markers in different clusters.

(D) Dot plot showing expression of recognized markers in different clusters.

(E) Violin plot showing expression of *HSPB1* across all clusters.

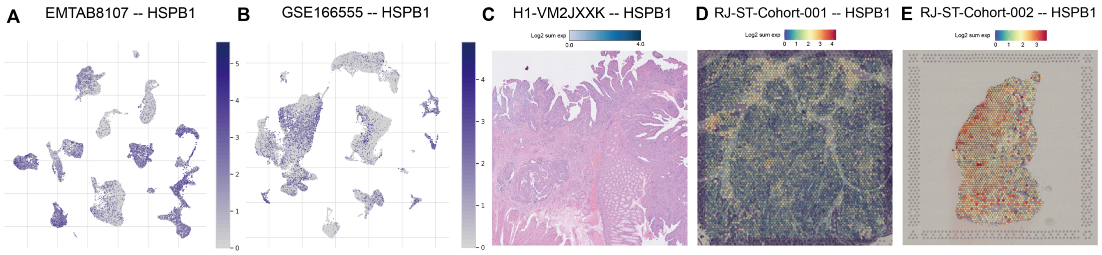


**Supplementary Figure 16. Expression of HSPB1 in single-cell and spatial transcriptomic datasets (related to Figure 6)**

(A-B) Expression patterns of *HSPB1* in EMTAB8107 dataset (A) and GSE166555 dataset (B). (C-E) Spatial distribution pattern of *HSPB1* in H1-VM2JXXK (C), RJ-ST-Cohort-001 (D) and RJ-ST-Cohort-002 (E) datasets.

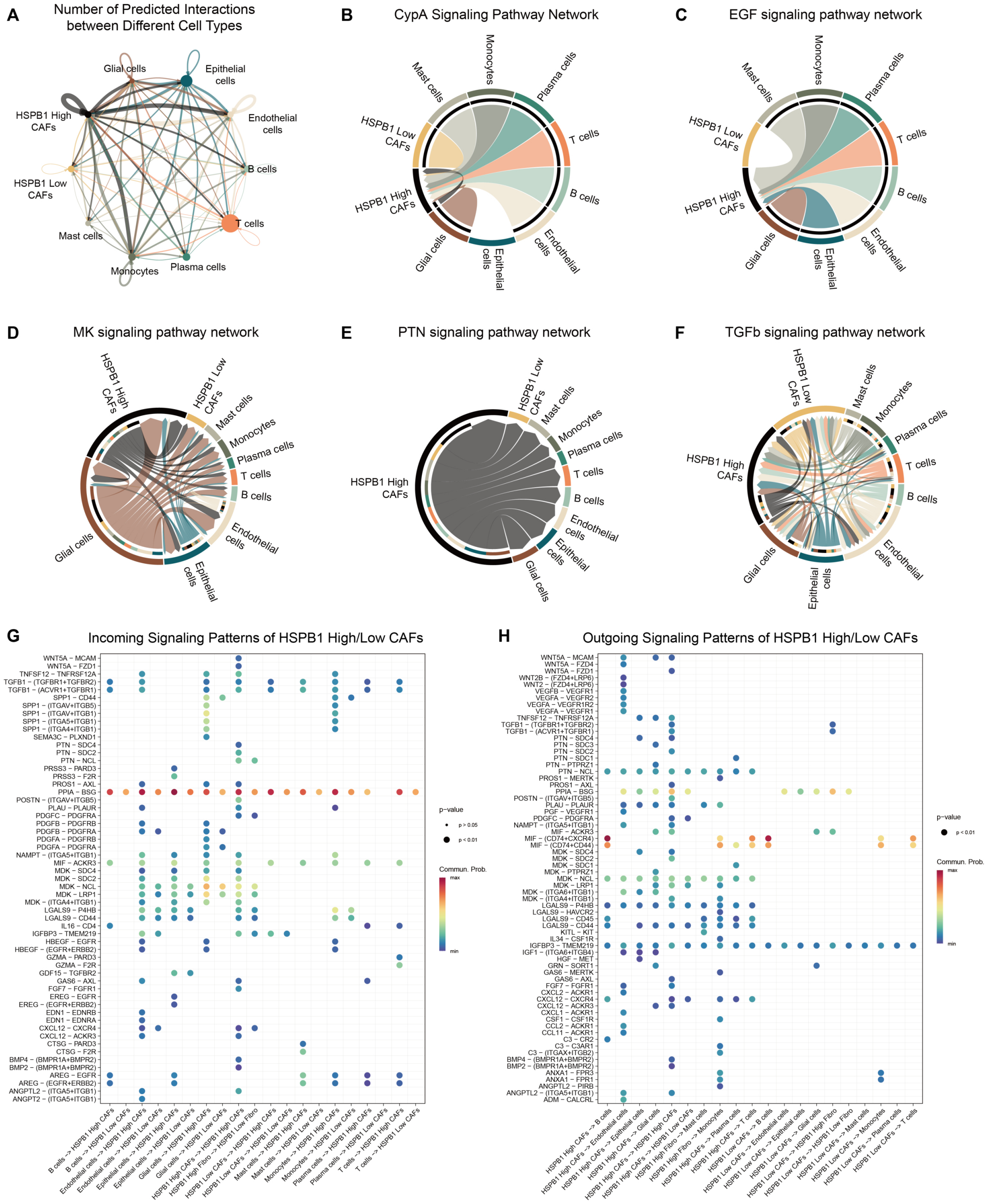


**Supplementary Figure 17. Cell–cell communication analyses of CRC samples (related to Figure 6)**

(A) Prediction of cell–cell communication among different cell clusters. The thickness of each line indicates the number of predicted interactions.

(B-F) Signaling networks for representative pathways including CypA (B), EGF (C), MK (D), PTN (E), and TGFβ (F). The thickness of each chord corresponds to the predicted interaction count between two cell types.

(G-H) Incoming (G) and outgoing (H) signaling patterns of HSPB1 high and low CAFs.

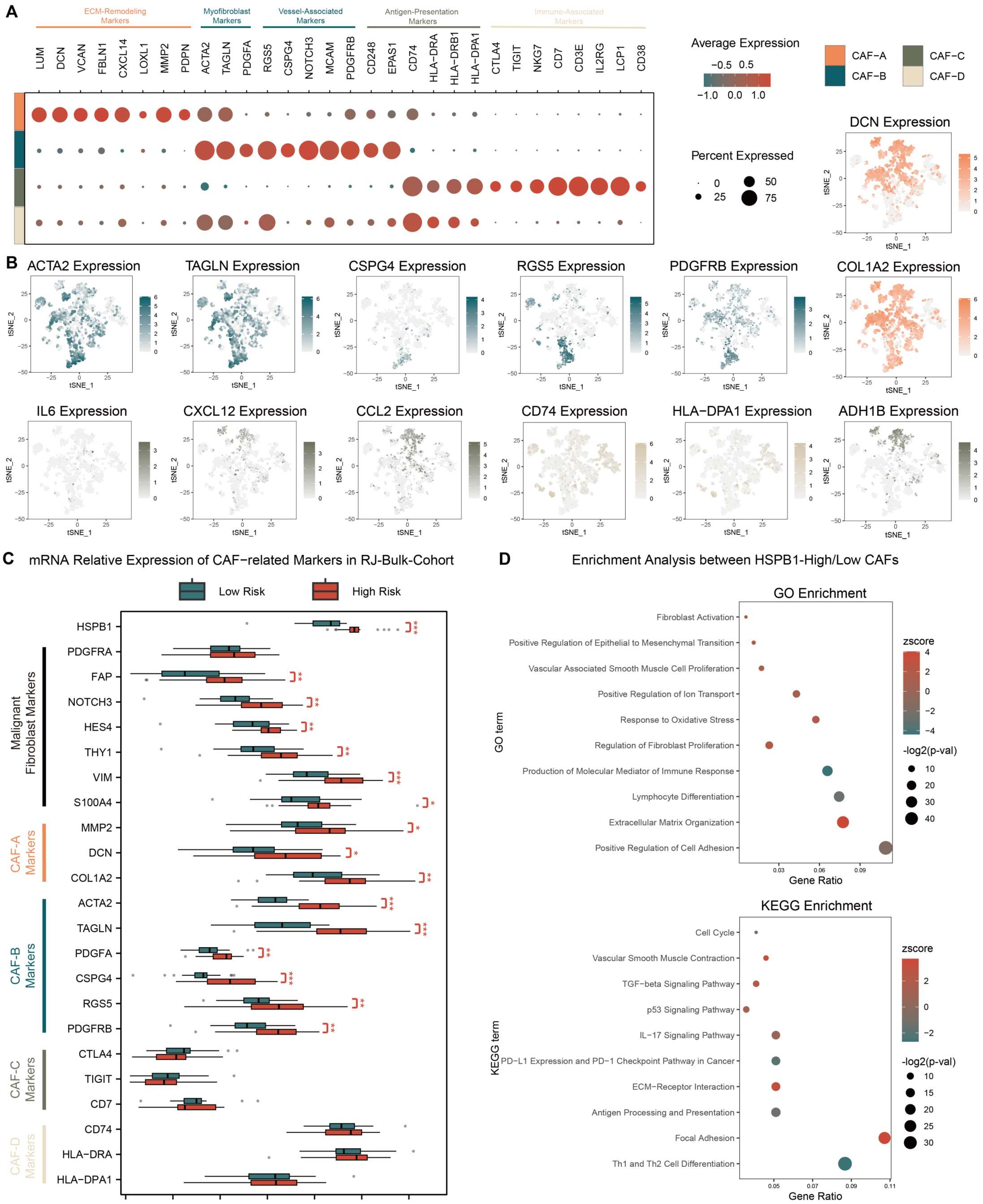


**Supplementary Figure 18. Additional insights into HSPB1 expression, CAF subtypes and functions (related to Figure 7)**

(A) Dot plot showing expression of significant markers in different CAF subtypes.
(B) t-SNE plot showing expression of significant markers in different CAF subtypes.
(C) Boxplot showing expressions of *HSPB1*, malignant fibroblast markers and markers of different CAF subtypes in high- and low-risk groups in RJ-BK-Cohort.
(D) GO and KEGG analysis of the DEGs between *HSPB1*$^{high}$ and *HSPB*1$^{low}$ CAFs.
Statistical significance was calculated using Wilcoxon test (C). * P < 0.05; ** P < 0.01; *** P < 0.001.

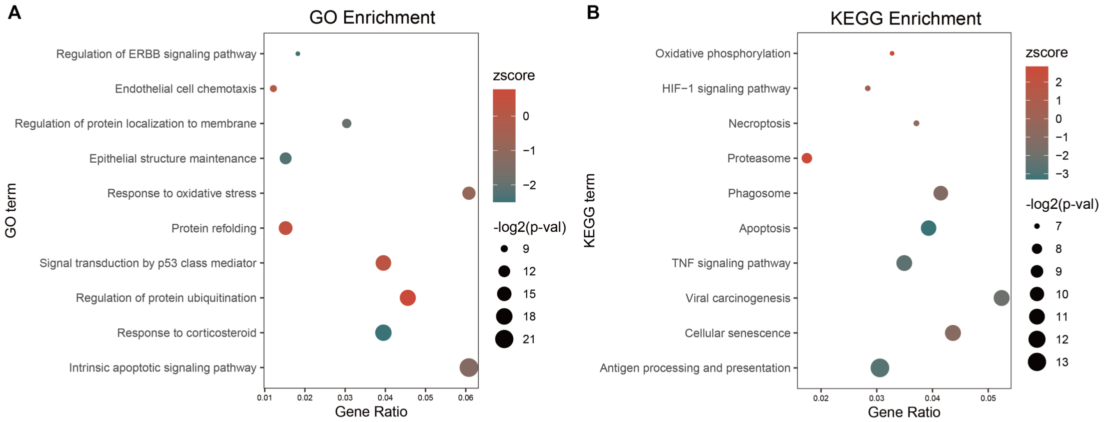


**Supplementary Figure 19. Pathway enrichment analysis between HSPB1$^{high}$ and HSPB1$^{low}$ epithelial cells**

(A-B) GO (A) and KEGG (B) analysis of the DEGs between *HSPB1*$^{high}$ and *HSPB*1$^{low}$ epithelial cells.

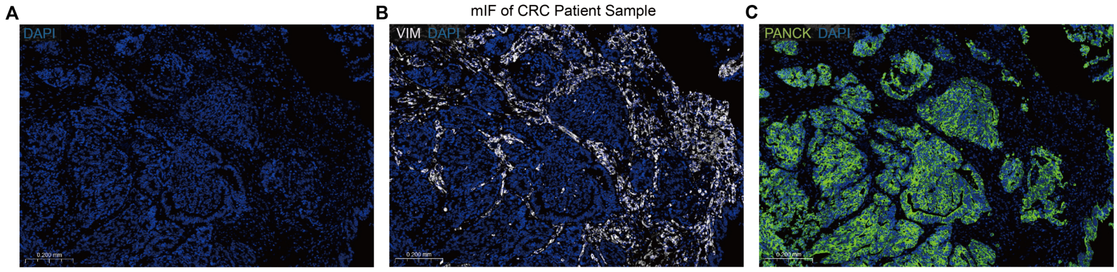


**Supplementary Figure 20. Representative mIF images of CRC patient samples (related to Figure 8)**

(A-C) The images showing DAPI staining (A) for nuclear visualization, Vimentin (VIM) staining (B) to identify stromal cells, and PANCK staining (C) for epithelial cells.

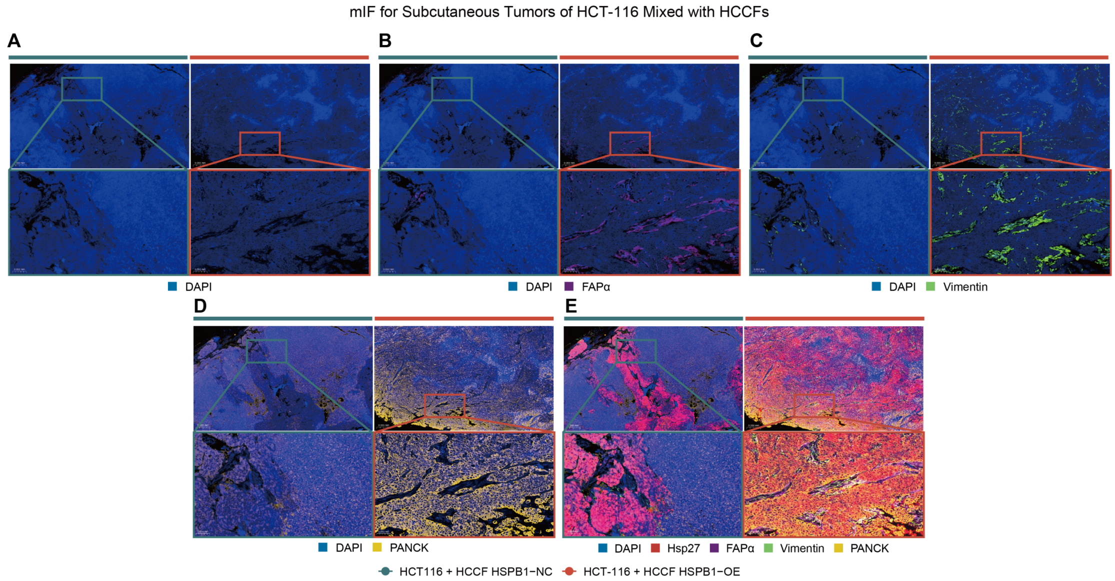


**Supplementary Figure 21. Representative mIF images of mice subcutaneous xenograft tumors (related to Figure 8)**

(A-E) The figures depict DAPI staining (A) for nuclear visualization, FAP staining (B) for CAFs, Vimentin (VIM) staining (C) to identify stromal cells, and PANCK staining (D) for epithelial cells, and a merged image showing all markers together (E)

**Table S1. Literature evidence supporting the selection of 11 recognized CAF markers**

| Gene | Review 1 (PMID) | Review 2 (PMID) | Experimental/Spatial validation (PMID) |
|---|---|---|---|
| ACTA2 | PMID: 33292666 | PMID: 27151975 | PMID: 36856115 |
| FAP | PMID: 33292666 | PMID: 27151975 | PMID: 38335304 |
| PDPN | PMID: 33292666 | PMID: 34489603 | PMID: 38335304 |
| SPARC | PMID: 24560651 | PMID: 24141189 | PMID: 17235047 |
| PDGFRB | PMID: 33292666 | PMID: 24560651 | PMID: 31287804 |
| PDGFRA | PMID: 33292666 | PMID: 24560651 | PMID: 38428409 |
| CAV1 | PMID: 33292666 | PMID: 24486645 | PMID: 21729786 |
| ZEB1 | PMID: 32466724 | PMID: 27151975 | PMID: 29490942 |
| FN1 | PMID: 35883004 | PMID: 27151975 | PMID: 38060213 |
| MMP2 | PMID: 35883004 | PMID: 38060213 | PMID: 28911071 |
| FOXF1 | PMID: 24560651 | PMID: 27151975 | PMID: 20233876 |

**Table S2. Marker genes of different cells in CRC provided by H1-VM2JXXK**

| Cell type | Markers |
|---|---|
| Fibroblast | COL1A1, MMP2 |
| Tumor cell | REG1A, REG1B, CEACAM6, TGFBI |

**Table S3. CAF-related genes for model construction**

| Gene Categories | Genes |
|---|---|
| CAFs candidate genes | COL1A1, COL3A1, COL1A2, MGP, LUM, CXCL14, ADAMDEC1, CFD, DCN, CLU, SFRP2, COL6A3, IGFBP7, SPARC, C1R, C1S, COL6A2, IGFBP5, FBLN1, POSTN, CCL11, COL6A1, PMP22, TIMP1, FXYD1, CCL13, SERPINF1, RARRES2, CCDC80, PTGDS, CALD1, A2M, MMP2, FN1, LGALS1, CTSK, TIMP3, NNMT, C11orf96, IGFBP6, APOD, THY1, COL5A2, APOE, ADH1B, AEBP1, CTGF, COL14A1, SPARCL1, BGN, C3, FSTL1, PCOLCE, VCAN, EMILIN1, MAP1B, SERPING1, COL18A1, CCL2, SPON2, SULF1, CFH, CYR61, C7, SERPINE2, FBN1, TIMP2, COL12A1, COL5A1, CTHRC1, MMP11, PDGFRA, DPT, TPM2, MATN2, LTBP4, PLAT, SFRP4, ASPN, PLAC9, F3, GREM1, EFEMP1, MXRA5, TAGLN, CEBPD, GSN, SFRP1, COL8A1, CCL8, LRP1, MXRA8, MFGE8, THBS2, CDH11, MYL9, SOD3, CXCL1, COL4A2, SCARA5, ADAMTS1, ANTXR1, S100A13, LAPTM4A, SDC2, TFPI, ABCA8, ISLR, WISP2, CLEC11A, OGN, ACTA2, NFIA, PALLD, RBP1, CYBRD1, IGF1, PTN, FHL1, RGS16, COL4A1, PPIC, FBLN2, S100A4, NBL1, CD63, PLTP, FGF7, PDPN, SORBS2, NEXN, TSHZ2, ID3, GPNMB, TNC, FBLN5, ANGPTL2, NUPR1, SLIT3, CAV1, MFAP4, CD248, MYH11, ADIRF, MCAM, RERGL, CSRP2, DSTN, NOTCH3, MYLK, PLN, FLNA, NDUFA4L2, DES, LMOD1, ACTG2, CSRP1, NR2F2, TPM1, FRZB, PDGFRB, CPE, TNS1, TSC22D1, PPP1R14A, ADAMTS4, CRIP2, SNCG, CAV2, LPP, GADD45B, BCAM, GJA4, TINAGL1, TGFB1I1, CNN1, ITGA1, PPP1R12B, ITGB1, MT2A, SYNPO2, COX7A1, EGR1, MT1X, HSPB1, CARMN, PDLIM3, C2orf40, COX4I2, MEF2C, MAP3K7CL, MT1M, MYL6 |
| CAF-related prognostic genes | BCAM, CNN1, MGP, ADAMTS4, NDUFA4L2, TPM2, TAGLN, FLNA, COX4I2, HSPB1, CRIP2, CSRP2, PDLIM3, COX7A1, APOE, CCL11, ASPN, CLU, PDGFRA, LMOD1, TPM1, SPARCL1 |

**Table S4. 58 published CRC prognostic predictive signatures**

| PMID | Author | Symbol | Coef |
|---|---|---|---|
| PMID : 31387239 | Ahluwalia | YWHAB | 1.324419 |
| PMID : 31387239 | Ahluwalia | MCM4 | 0.989541 |
| PMID : 31387239 | Ahluwalia | LRRC59 | 0.841567 |
| PMID : 31387239 | Ahluwalia | DPP7 | -0.96758 |
| PMID : 34073426 | Busuioc | BMP5 | -0.27063 |
| PMID : 34073426 | Busuioc | DACT3 | 0.11034 |
| PMID : 34073426 | Busuioc | EEF1A2 | 0.121492 |
| PMID : 34073426 | Busuioc | GCNT2 | -0.0115 |
| PMID : 34073426 | Busuioc | IGF2BP3 | 0.082514 |
| PMID : 34073426 | Busuioc | NOX4 | 0.049782 |
| PMID : 34073426 | Busuioc | SFRP1 | -0.05039 |
| PMID : 34211990 | Chen-FM | ORAI3 | 0.052 |
| PMID : 34211990 | Chen-FM | CYB5R3 | 0.005 |
| PMID : 34211990 | Chen-FM | DTX3L | -0.003 |
| PMID : 34211990 | Chen-FM | TNFAIP8 | -0.006 |
| PMID : 34211990 | Chen-FM | CASP6 | -0.019 |
| PMID : 34211990 | Chen-FM | MBTD1 | -0.023 |
| PMID : 34211990 | Chen-FM | PRELID2 | -0.023 |
| PMID : 34211990 | Chen-FM | ZBTB44 | -0.05 |
| PMID : 34211990 | Chen-FM | FAM117B | -0.059 |
| PMID : 34211990 | Chen-FM | IRF1 | -0.07 |
| PMID : 34211990 | Chen-FM | MINPP1 | -0.079 |
| PMID : 34211990 | Chen-FM | TRAF3 | -0.087 |
| PMID : 30654001 | Chen-Gene | TIMP1 | 0.3424 |
| PMID : 30654001 | Chen-Gene | GLDN | 0.2082 |
| PMID : 30654001 | Chen-Gene | CCL28 | 0.1149 |
| PMID : 30654001 | Chen-Gene | PCOLCE2 | 0.0834 |
| PMID : 30654001 | Chen-Gene | SCGB2A1 | -0.0991 |
| PMID : 30654001 | Chen-Gene | SLC4A4 | -0.1068 |
| PMID : 30654001 | Chen-Gene | AXIN2 | -0.108 |
| PMID : 30654001 | Chen-Gene | MMP1 | -0.1516 |
| PMID : 30654001 | Chen-Gene | NFE2L3 | -0.2564 |
| PMID : 29221110 | Chen-ONCO | PTPRB | -0.008 |
| PMID : 29221110 | Chen-ONCO | NHLRC3 | -0.133 |
| PMID : 29221110 | Chen-ONCO | PNPO | -0.715 |
| PMID : 29221110 | Chen-ONCO | ZDHHC21 | -0.781 |
| PMID : 29221110 | Chen-ONCO | PPIP5K2 | -1.004 |
| PMID : 29221110 | Chen-ONCO | PRR14L | -1.077 |
| PMID : 29221110 | Chen-ONCO | KYAT1 | 0.823 |
| PMID : 26989635 | ColoFinder | APC | NA |
| PMID : 26989635 | ColoFinder | KRAS | NA |
| PMID : 26989635 | ColoFinder | MLH1 | NA |

| | | | |
|---|---|---|---|
| PMID : 26989635 | ColoFinder | MSH2 | NA |
| PMID : 26989635 | ColoFinder | MSH6 | NA |
| PMID : 26989635 | ColoFinder | PTEN | NA |
| PMID : 26989635 | ColoFinder | SMAD4 | NA |
| PMID : 26989635 | ColoFinder | TGFBR2 | NA |
| PMID : 26989635 | ColoFinder | TP53 | NA |
| PMID : 22991413 | ColoGuidePro | SEMA3A | 0.1 |
| PMID : 22991413 | ColoGuidePro | WNT11 | 0.1 |
| PMID : 22991413 | ColoGuidePro | NT5E | 0.05 |
| PMID : 22991413 | ColoGuidePro | DMBT1 | 0.04 |
| PMID : 22991413 | ColoGuidePro | UGT2B17 | -0.02 |
| PMID : 22991413 | ColoGuidePro | OLFM4 | -0.08 |
| PMID : 22991413 | ColoGuidePro | CXCL9 | -0.1 |
| PMID : 25749516 | ColoLipidGene | ABCA1 | -0.02626 |
| PMID : 25749516 | ColoLipidGene | ACSL1 | 0.004694 |
| PMID : 25749516 | ColoLipidGene | AGPAT1 | 0.191265 |
| PMID : 25749516 | ColoLipidGene | SCD | 0.062064 |
| PMID : 33343640 | Dai-FIG | GPRC5B | 0.613 |
| PMID : 33343640 | Dai-FIG | FLOT1 | 0.599 |
| PMID : 33343640 | Dai-FIG | IL20RB | 0.596 |
| PMID : 33343640 | Dai-FIG | LIMK1 | 0.402 |
| PMID : 33343640 | Dai-FIG | CCL22 | -0.421 |
| PMID : 33343640 | Dai-FIG | MAPKAPK3 | -0.465 |
| PMID : 29377588 | Dai-MO | ECM1 | 0.259 |
| PMID : 29377588 | Dai-MO | KLK10 | 0.201 |
| PMID : 29377588 | Dai-MO | GZMB | 0.043 |
| PMID : 29377588 | Dai-MO | MSLN | 0.038 |
| PMID : 29377588 | Dai-MO | KRT6A | -0.015 |
| PMID : 29377588 | Dai-MO | CCL20 | -0.047 |
| PMID : 29377588 | Dai-MO | ACTR3B | -0.052 |
| PMID : 29377588 | Dai-MO | IL7 | -0.102 |
| PMID : 29377588 | Dai-MO | BLMH | -0.116 |
| PMID : 29377588 | Dai-MO | CMPK2 | -0.121 |
| PMID : 29377588 | Dai-MO | ZNF426 | -0.168 |
| PMID : 29377588 | Dai-MO | OAS1 | -0.217 |
| PMID : 29377588 | Dai-MO | PUS7 | -0.236 |
| PMID : 29377588 | Dai-MO | HES6 | -0.287 |
| PMID : 29377588 | Dai-MO | MMP9 | -0.302 |
| PMID : 30655785 | Dong | LRP2 | 0.253 |
| PMID : 30655785 | Dong | LEF1 | 0.139 |
| PMID : 30655785 | Dong | IGFBP3 | 0.084 |
| PMID : 30655785 | Dong | HEYL | 0.062 |
| PMID : 30655785 | Dong | GADD45B | 0.058 |
| PMID : 30655785 | Dong | CUBN | 0.048 |

| | | | |
|---|---|---|---|
| PMID : 30655785 | Dong | SFRP2 | 0.036 |
| PMID : 30655785 | Dong | CCNE1 | -0.19 |
| PMID : 33203797 | Fu | POU4F1 | 1.204033 |
| PMID : 33203797 | Fu | MAGEA1 | 1.127661 |
| PMID : 33203797 | Fu | NOVA1 | 1.127221 |
| PMID : 33203797 | Fu | IZUMO2 | 0.864582 |
| PMID : 33203797 | Fu | SLCO4C1 | 0.800341 |
| PMID : 30881123 | Ge | ACVR2A | 0.051533 |
| PMID : 30881123 | Ge | APC | -0.09079 |
| PMID : 30881123 | Ge | DOCK2 | -0.00693 |
| PMID : 30881123 | Ge | POLE | 0.008983 |
| PMID : 32155177 | Gharib | ACOT8 | 0.659 |
| PMID : 32155177 | Gharib | HMGCS2 | 0.201 |
| PMID : 32155177 | Gharib | ACSL5 | 0.084 |
| PMID : 32155177 | Gharib | FASN | 0.029 |
| PMID : 32155177 | Gharib | SCD | 0.119 |
| PMID : 33377637 | Goeman | APC | 0.01945 |
| PMID : 33377637 | Goeman | CSNK1A1 | -0.29152 |
| PMID : 33377637 | Goeman | DKK1 | 0.035828 |
| PMID : 33377637 | Goeman | PRICKLE1 | 0.104051 |
| PMID : 33377637 | Goeman | SFRP1 | -0.1236 |
| PMID : 33377637 | Goeman | SFRP2 | 0.279682 |
| PMID : 33377637 | Goeman | SOX17 | 0.012343 |
| PMID : 20077526 | Hao | DKK1 | -0.01056 |
| PMID : 20077526 | Hao | LYN | -0.14549 |
| PMID : 20077526 | Hao | MAP4K4 | 0.115278 |
| PMID : 20077526 | Hao | MID1 | -0.12727 |
| PMID : 20077526 | Hao | SDCBP | 0.031025 |
| PMID : 34193169 | He | RPL3L | 2.641958 |
| PMID : 34193169 | He | CAPRIN2 | 0.19416 |
| PMID : 34193169 | He | CCAR2 | -0.06165 |
| PMID : 34193169 | He | GSPT1 | -0.06582 |
| PMID : 34193169 | He | MRPS18C | -0.45463 |
| PMID : 34178621 | Huang | BATF | 0.253 |
| PMID : 34178621 | Huang | PHYHIPL | 0.147 |
| PMID : 34178621 | Huang | RBP1 | -0.172 |
| PMID : 34178621 | Huang | PNPLA4 | -0.183 |
| PMID : 32500031 | Ji | ALKBH5 | 0.57098 |
| PMID : 32500031 | Ji | YTHDC2 | -0.41552 |
| PMID : 31578316 | Kim | TERF2IP | 0.887 |
| PMID : 31578316 | Kim | ITGB1 | 0.758 |
| PMID : 31578316 | Kim | EIF4A2 | 0.543 |
| PMID : 31578316 | Kim | MAP4K4 | 0.539 |
| PMID : 31578316 | Kim | RHOC | 0.33 |

| | | | |
|---|---|---|---|
| PMID : 31578316 | Kim | HSPB1 | 0.319 |
| PMID : 31578316 | Kim | MMP12 | -0.133 |
| PMID : 31578316 | Kim | CDC25A | -0.363 |
| PMID : 31578316 | Kim | BID | -0.504 |
| PMID : 31578316 | Kim | AK2 | -0.536 |
| PMID : 31578316 | Kim | PTGES3 | -0.696 |
| PMID : 32494194 | Li-CMS | ITGB1 | 0.6432 |
| PMID : 32494194 | Li-CMS | SIX4 | 0.3413 |
| PMID : 32494194 | Li-CMS | PIK3CA | 0.2717 |
| PMID : 32494194 | Li-CMS | ITGA5 | 0.1874 |
| PMID : 32494194 | Li-CMS | NLGN3 | 0.1382 |
| PMID : 32494194 | Li-CMS | CBLN2 | 0.127 |
| PMID : 32494194 | Li-CMS | DKK1 | 0.0732 |
| PMID : 32494194 | Li-CMS | CXCL5 | -0.02879 |
| PMID : 32494194 | Li-CMS | WNT5A | -0.0768 |
| PMID : 32494194 | Li-CMS | CXCL2 | -0.1207 |
| PMID : 32494194 | Li-CMS | CXCL1 | -0.1391 |
| PMID : 32494194 | Li-CMS | EPHB3 | -0.1591 |
| PMID : 32494194 | Li-CMS | LRTM1 | -0.282 |
| PMID : 32494194 | Li-CMS | EPHB2 | -0.311 |
| PMID : 32494194 | Li-CMS | PTRH2 | -0.4696 |
| PMID : 34017356 | Li-FIG | LIF | 0.766 |
| PMID : 34017356 | Li-FIG | IL23A | 0.501 |
| PMID : 34017356 | Li-FIG | VGF | 0.384 |
| PMID : 34017356 | Li-FIG | FGF18 | 0.351 |
| PMID : 34017356 | Li-FIG | SLIT2 | 0.179 |
| PMID : 34017356 | Li-FIG | CCL28 | -0.19 |
| PMID : 32771948 | Li-IntI | UCN | 0.50362 |
| PMID : 32771948 | Li-IntI | STC1 | 0.20628 |
| PMID : 32771948 | Li-IntI | PTH1R | 0.16894 |
| PMID : 32771948 | Li-IntI | INHBB | 0.16237 |
| PMID : 32771948 | Li-IntI | SCG2 | 0.09357 |
| PMID : 32771948 | Li-IntI | ULBP2 | 0.09061 |
| PMID : 32771948 | Li-IntI | HAMP | 0.01244 |
| PMID : 32771948 | Li-IntI | GDF15 | -0.00615 |
| PMID : 32771948 | Li-IntI | PCSK1 | -0.02415 |
| PMID : 32771948 | Li-IntI | NR3C2 | -0.06016 |
| PMID : 32771948 | Li-IntI | IL1A | -0.0618 |
| PMID : 32771948 | Li-IntI | TG | -0.07016 |
| PMID : 32771948 | Li-IntI | UCN3 | -0.07878 |
| PMID : 32771948 | Li-IntI | ZC3HAV1L | -0.10207 |
| PMID : 32771948 | Li-IntI | GLP2R | -0.11758 |
| PMID : 32771948 | Li-IntI | ORM1 | -0.11964 |
| PMID : 32888940 | Li-LS | IL27 | 1.77781 |

| | | | |
|---|---|---|---|
| PMID : 32888940 | Li-LS | CD19 | 0.36491 |
| PMID : 32888940 | Li-LS | LAYN | 0.27201 |
| PMID : 32888940 | Li-LS | IL20RB | 0.07975 |
| PMID : 32888940 | Li-LS | TNFRSF19 | 0.0642 |
| PMID : 32888940 | Li-LS | GRSF1 | -0.04829 |
| PMID : 32888940 | Li-LS | CCL22 | -0.57391 |
| PMID : 34080453 | Li-TCRT | CELF4 | 1.1488 |
| PMID : 34080453 | Li-TCRT | LUZP4 | 0.7699 |
| PMID : 34080453 | Li-TCRT | PNLDC1 | 0.6121 |
| PMID : 34080453 | Li-TCRT | TERT | 0.5093 |
| PMID : 34080453 | Li-TCRT | PPARGC1A | -0.7152 |
| PMID : 34080453 | Li-TCRT | TDRD7 | -0.799 |
| PMID : 34080453 | Li-TCRT | BRCA1 | -0.8181 |
| PMID : 34080453 | Li-TCRT | ZC3H12C | -0.8421 |
| PMID : 34169901 | Liang | IGF2BP3 | 0.131416 |
| PMID : 34169901 | Liang | PABPC1L | 0.117148 |
| PMID : 34169901 | Liang | PPARGC1A | -0.20997 |
| PMID : 34169901 | Liang | PTRH1 | 0.209449 |
| PMID : 34169901 | Liang | TDRD7 | -0.23512 |
| PMID : 34026619 | Lin-FIO | ALCAM | 0.816 |
| PMID : 34026619 | Lin-FIO | CASP1 | -0.323 |
| PMID : 34026619 | Lin-FIO | CISH | -2.23 |
| PMID : 34026619 | Lin-FIO | CD22 | -2.889 |
| PMID : 34193202 | Lin-JTM | INHBB | 0.198099 |
| PMID : 34193202 | Lin-JTM | ABCD4 | 0.103628 |
| PMID : 34193202 | Lin-JTM | PLCE1 | 0.062357 |
| PMID : 34193202 | Lin-JTM | RPS25 | 0.031996 |
| PMID : 34193202 | Lin-JTM | PLA2G4D | 0.012935 |
| PMID : 34193202 | Lin-JTM | CYP2D6 | 0.004666 |
| PMID : 34193202 | Lin-JTM | NPR2 | 0.001148 |
| PMID : 34193202 | Lin-JTM | ALOX12B | -0.00171 |
| PMID : 34193202 | Lin-JTM | CS | -0.00286 |
| PMID : 34193202 | Lin-JTM | AMACR | -0.00477 |
| PMID : 34193202 | Lin-JTM | POMT1 | -0.01521 |
| PMID : 34193202 | Lin-JTM | NHP2 | -0.01683 |
| PMID : 34193202 | Lin-JTM | LIPG | -0.02119 |
| PMID : 34193202 | Lin-JTM | DDX52 | -0.03704 |
| PMID : 34193202 | Lin-JTM | METTL2B | -0.05133 |
| PMID : 34193202 | Lin-JTM | ACOX2 | -0.0567 |
| PMID : 34193202 | Lin-JTM | PSME1 | -0.08558 |
| PMID : 34193202 | Lin-JTM | OGDHL | -0.09337 |
| PMID : 33674956 | Liu-CEM | DUOX1 | 0.4796 |
| PMID : 33674956 | Liu-CEM | DRD4 | 0.3747 |
| PMID : 33674956 | Liu-CEM | ALOX12B | 0.2338 |

| | | | |
|---|---|---|---|
| PMID : 33674956 | Liu-CEM | JDP2 | 0.1205 |
| PMID : 33674956 | Liu-CEM | TFAP2C | 0.1149 |
| PMID : 33674956 | Liu-CEM | ATG13 | 0.0462 |
| PMID : 33674956 | Liu-CEM | CAV1 | 0.01 |
| PMID : 33674956 | Liu-CEM | PLIN4 | 0.0091 |
| PMID : 33674956 | Liu-CEM | FDFT1 | -0.0137 |
| PMID : 33674956 | Liu-CEM | NOS2 | -0.0206 |
| PMID : 33282950 | Luo | GSTM2 | 0.568972 |
| PMID : 33282950 | Luo | ACADL | 0.326618 |
| PMID : 33282950 | Luo | FTCD | 0.313805 |
| PMID : 33282950 | Luo | PDE1B | 0.260516 |
| PMID : 33282950 | Luo | PLCG2 | 0.251929 |
| PMID : 33282950 | Luo | GSTM5 | 0.206249 |
| PMID : 33282950 | Luo | DGKB | 0.190773 |
| PMID : 33282950 | Luo | PDE6B | 0.150215 |
| PMID : 33282950 | Luo | MAT1A | 0.130179 |
| PMID : 33282950 | Luo | CPT1C | 0.123892 |
| PMID : 33282950 | Luo | CDS1 | -0.0706 |
| PMID : 33282950 | Luo | AGMAT | -0.09553 |
| PMID : 33282950 | Luo | ALDOB | -0.11182 |
| PMID : 33282950 | Luo | MTMR7 | -0.22176 |
| PMID : 33282950 | Luo | SGPP2 | -0.3518 |
| PMID : 33282950 | Luo | GPX2 | -0.41768 |
| PMID : 33282950 | Luo | HDC | -0.63059 |
| PMID : 33732694 | Mao | PLAU | 0.3049 |
| PMID : 33732694 | Mao | BMP4 | 0.1927 |
| PMID : 33732694 | Mao | IL1R2 | -0.0968 |
| PMID : 33732694 | Mao | LGR5 | -0.1572 |
| PMID : 33732694 | Mao | CXCL3 | -0.1689 |
| PMID : 33732694 | Mao | GZMB | -0.2983 |
| PMID : 33732694 | Mao | PTGDR | -0.5107 |
| PMID : 30537927 | Martinez | CSGALNACT2 | -0.19221 |
| PMID : 30537927 | Martinez | DCBLD2 | 0.027363 |
| PMID : 30537927 | Martinez | GADD45B | 0.052055 |
| PMID : 30537927 | Martinez | LAMP5 | 0.281696 |
| PMID : 30537927 | Martinez | LCA5 | -0.12455 |
| PMID : 30537927 | Martinez | LEMD1 | 0.016504 |
| PMID : 30537927 | Martinez | NPR3 | -0.07333 |
| PMID : 30537927 | Martinez | PTPN14 | 0.301624 |
| PMID : 30537927 | Martinez | SLC2A3 | 0.089495 |
| PMID : 30537927 | Martinez | TM4SF1 | -0.11599 |
| PMID : 32953273 | Miao | ADCY5 | 0.1465 |
| PMID : 32953273 | Miao | AKR1C4 | 0.0283 |
| PMID : 32953273 | Miao | GPX3 | 0.011 |

| | | | |
|---|---|---|---|
| PMID : 32953273 | Miao | SPHK1 | 0.0075 |
| PMID : 32953273 | Miao | XDH | -0.0293 |
| PMID : 32953273 | Miao | NAT2 | -0.0631 |
| PMID : 30933267 | Mo-Car1 | CAPN2 | 5.24 |
| PMID : 30933267 | Mo-Car1 | TP63 | 3.067 |
| PMID : 30933267 | Mo-Car1 | ATG5 | 0.562 |
| PMID : 30933267 | Mo-Car1 | RPS6KB1 | -0.045 |
| PMID : 30933267 | Mo-Car1 | ATG16L2 | -0.095 |
| PMID : 30933267 | Mo-Car1 | SIRT1 | -0.178 |
| PMID : 30933267 | Mo-Car1 | PEX3 | -0.696 |
| PMID : 30933267 | Mo-Car1 | UVRAG | -0.957 |
| PMID : 30933267 | Mo-Car1 | NAF1 | -1.124 |
| PMID : 30933267 | Mo-Car2 | CAPN2 | 0.795 |
| PMID : 30933267 | Mo-Car2 | TP63 | 0.407 |
| PMID : 30933267 | Mo-Car2 | ATG5 | 0.126 |
| PMID : 30933267 | Mo-Car2 | PEX3 | -0.009 |
| PMID : 30933267 | Mo-Car2 | UVRAG | -0.018 |
| PMID : 30933267 | Mo-Car2 | ATG16L2 | -0.079 |
| PMID : 30933267 | Mo-Car2 | NAF1 | -0.096 |
| PMID : 30933267 | Mo-Car2 | SIRT1 | -0.324 |
| PMID : 30933267 | Mo-Car2 | RPS6KB1 | -0.412 |
| PMID : 34211976 | Mo-FICDB | ITGB1 | 0.770095 |
| PMID : 34211976 | Mo-FICDB | CDH6 | 0.765924 |
| PMID : 34211976 | Mo-FICDB | ACTA2 | 0.631001 |
| PMID : 34211976 | Mo-FICDB | DST | 0.499476 |
| PMID : 34211976 | Mo-FICDB | MAGEE1 | 0.494134 |
| PMID : 34211976 | Mo-FICDB | FUCA1 | 0.431895 |
| PMID : 34211976 | Mo-FICDB | GJA1 | 0.227362 |
| PMID : 34211976 | Mo-FICDB | PLOD3 | 0.222921 |
| PMID : 34211976 | Mo-FICDB | CRLF1 | 0.218153 |
| PMID : 34211976 | Mo-FICDB | NT5E | 0.215944 |
| PMID : 34211976 | Mo-FICDB | DKK1 | 0.186114 |
| PMID : 34211976 | Mo-FICDB | LAMC2 | 0.134651 |
| PMID : 34211976 | Mo-FICDB | CXCL1 | -0.16135 |
| PMID : 34211976 | Mo-FICDB | CXCL12 | -0.20579 |
| PMID : 34211976 | Mo-FICDB | MEST | -0.20607 |
| PMID : 34211976 | Mo-FICDB | IL15 | -0.29396 |
| PMID : 34211976 | Mo-FICDB | DPYSL3 | -0.30171 |
| PMID : 34211976 | Mo-FICDB | PLOD2 | -0.30637 |
| PMID : 34211976 | Mo-FICDB | LAMA2 | -0.35871 |
| PMID : 33117604 | Mo-OI | SKAP1 | 0.665 |
| PMID : 33117604 | Mo-OI | S100A4 | 0.329 |
| PMID : 33117604 | Mo-OI | FGF19 | 0.319 |
| PMID : 33117604 | Mo-OI | TACSTD2 | 0.182 |

| | | | |
|---|---|---|---|
| PMID : 33117604 | Mo-OI | LGR5 | -0.228 |
| PMID : 33117604 | Mo-OI | PSMD3 | -0.939 |
| PMID : 33117604 | Mo-OI | CCL22 | -1.304 |
| PMID : 33660944 | Shan | FLNA | -0.08217 |
| PMID : 33660944 | Shan | SPOCK1 | 0.287634 |
| PMID : 33660944 | Shan | WWTR1 | 0.204543 |
| PMID : 33660944 | Shan | SERPINE1 | 0.164739 |
| PMID : 33660944 | Shan | FSCN1 | 0.11085 |
| PMID : 33660944 | Shan | EFEMP1 | 0.085466 |
| PMID : 33660944 | Shan | NOX1 | -0.04164 |
| PMID : 33660944 | Shan | CXCL8 | -0.18518 |
| PMID : 33660944 | Shan | VIM | -0.70744 |
| PMID : 33660944 | Shan | C5AR1 | 0.376991 |
| PMID : 32953885 | Sun-BRI | ACADL | 7.063052 |
| PMID : 32953885 | Sun-BRI | AOC2 | 0.813994 |
| PMID : 32953885 | Sun-BRI | ENPP2 | 0.065392 |
| PMID : 32953885 | Sun-BRI | ADA | 0.060378 |
| PMID : 32953885 | Sun-BRI | GPD1L | -0.05543 |
| PMID : 32953885 | Sun-BRI | CPT2 | -0.10342 |
| PMID : 30132881 | Sun-JCP | SCG2 | 0.065227 |
| PMID : 30132881 | Sun-JCP | GPX3 | 0.009504 |
| PMID : 30132881 | Sun-JCP | CLCA1 | 0.000338 |
| PMID : 30132881 | Sun-JCP | NAT2 | -0.08398 |
| PMID : 30132881 | Sun-JCP | KIF15 | -0.11953 |
| PMID : 32036725 | Wang-BIO | SLC4A2 | 0.861 |
| PMID : 32036725 | Wang-BIO | PMM2 | 0.837 |
| PMID : 32036725 | Wang-BIO | RNF113A | 0.603 |
| PMID : 32036725 | Wang-BIO | ZBTB34 | 0.527 |
| PMID : 32036725 | Wang-BIO | ANKRD16 | 0.474 |
| PMID : 32036725 | Wang-BIO | JAG2 | 0.342 |
| PMID : 32036725 | Wang-BIO | GJB6 | 0.15 |
| PMID : 32036725 | Wang-BIO | HCRTR1 | 0.144 |
| PMID : 32036725 | Wang-BIO | SOWAHA | -0.292 |
| PMID : 32036725 | Wang-BIO | MIPEP | -0.336 |
| PMID : 32036725 | Wang-BIO | KIF15 | -0.449 |
| PMID : 32036725 | Wang-BIO | RPP14 | -0.463 |
| PMID : 32036725 | Wang-BIO | TUBA1C | -0.609 |
| PMID : 32036725 | Wang-BIO | RPN2 | -0.78 |
| PMID : 32036725 | Wang-BIO | CLEC16A | -1.512 |
| PMID : 33224957 | Wang-FICDB | PNLDC1 | 0.34108 |
| PMID : 33224957 | Wang-FICDB | ZC3H12C | -0.58829 |
| PMID : 33224957 | Wang-FICDB | PPARGC1A | -0.8399 |
| PMID : 33224957 | Wang-FICDB | PABPC3 | -0.99781 |
| PMID : 33224957 | Wang-FICDB | TDRD7 | -1.06396 |

| | | | |
|---|---|---|---|
| PMID : 33224957 | Wang-FICDB | LRRFIP2 | -1.30029 |
| PMID : 33224957 | Wang-FICDB | TDRD6 | -2.01089 |
| PMID : 33719345 | Wang-FIG | CCNB3 | 3.5 |
| PMID : 33719345 | Wang-FIG | SMC1B | 0.48 |
| PMID : 33719345 | Wang-FIG | LSP1P4 | 0.34 |
| PMID : 33719345 | Wang-FIG | ELL3 | 0.3 |
| PMID : 33719345 | Wang-FIG | ISY1 | 0.27 |
| PMID : 33719345 | Wang-FIG | MC1R | 0.26 |
| PMID : 33719345 | Wang-FIG | CD36 | 0.19 |
| PMID : 33719345 | Wang-FIG | PTPN3 | 0.11 |
| PMID : 33719345 | Wang-FIG | POLG | 0.11 |
| PMID : 33719345 | Wang-FIG | TPM1 | -0.039 |
| PMID : 33719345 | Wang-FIG | CDC25C | -0.081 |
| PMID : 33719345 | Wang-FIG | NEK4 | -0.46 |
| PMID : 33910362 | Wang-FO | LINC00174 | 0.6742 |
| PMID : 33910362 | Wang-FO | TAF1A-AS1 | 0.4466 |
| PMID : 33910362 | Wang-FO | ZNF667-AS1 | 0.4241 |
| PMID : 33910362 | Wang-FO | LINC01560 | 0.3945 |
| PMID : 33910362 | Wang-FO | FOXP1-IT1 | -0.484 |
| PMID : 33910362 | Wang-FO | LINC00852 | -0.9109 |
| PMID : 33787596 | Wang-Med | DNAJB1 | 0.5989 |
| PMID : 33787596 | Wang-Med | PEA15 | 0.0973 |
| PMID : 33787596 | Wang-Med | VAMP7 | -0.1977 |
| PMID : 33787596 | Wang-Med | PEX14 | -0.1584 |
| PMID : 33787596 | Wang-Med | FKBP1B | 0.2351 |
| PMID : 33787596 | Wang-Med | DLC1 | 0.1991 |
| PMID : 32908876 | Wu | CCL22 | -0.42579 |
| PMID : 32908876 | Wu | CD36 | 0.178375 |
| PMID : 32908876 | Wu | CPA3 | -0.32944 |
| PMID : 32908876 | Wu | CPT1C | 0.120658 |
| PMID : 32908876 | Wu | KCNE4 | -0.15483 |
| PMID : 32908876 | Wu | NFATC1 | 0.242964 |
| PMID : 32908876 | Wu | RASGRP2 | 0.229709 |
| PMID : 32908876 | Wu | SLC2A3 | 0.217708 |
| PMID : 33663535 | Xia | HS2ST1 | 0.5377 |
| PMID : 33663535 | Xia | HAX1 | 0.6679 |
| PMID : 33663535 | Xia | HDLBP | 0.5384 |
| PMID : 33663535 | Xia | GNPDA1 | 0.4753 |
| PMID : 33663535 | Xia | STMN1 | 0.3651 |
| PMID : 33663535 | Xia | SRD5A3 | 0.3543 |
| PMID : 33663535 | Xia | RBCK1 | 0.3459 |
| PMID : 33663535 | Xia | CDK1 | -0.3001 |
| PMID : 33663535 | Xia | GPD1 | -0.3413 |
| PMID : 33663535 | Xia | NUP62 | -0.6322 |

| | | | |
|---|---|---|---|
| PMID : 33324651 | Xu | DAPK1 | 0.128135 |
| PMID : 33324651 | Xu | CTSD | 0.088102 |
| PMID : 33324651 | Xu | CDKN2A | 0.083553 |
| PMID : 33324651 | Xu | ATG4B | 0.070716 |
| PMID : 33324651 | Xu | ULK3 | 0.067559 |
| PMID : 33324651 | Xu | ULK1 | 0.019175 |
| PMID : 33324651 | Xu | NRG1 | -0.11921 |
| PMID : 33324651 | Xu | SERPINA1 | -0.15361 |
| PMID : 33537062 | Yang | SEPHS1 | 0.05689 |
| PMID : 33537062 | Yang | PODXL | 0.032757 |
| PMID : 33537062 | Yang | RHNO1 | 0.020453 |
| PMID : 33537062 | Yang | SCD | 0.002886 |
| PMID : 33537062 | Yang | PGM2 | -0.14089 |
| PMID : 33658390 | Yue-Aging | CD36 | 0.0405 |
| PMID : 33658390 | Yue-Aging | VEGFA | 0.0201 |
| PMID : 33658390 | Yue-Aging | PCSK5 | 0.0167 |
| PMID : 33658390 | Yue-Aging | FOXM1 | 0.0117 |
| PMID : 33658390 | Yue-Aging | IGFBP3 | 0.00373 |
| PMID : 33658390 | Yue-Aging | CLU | 0.00171 |
| PMID : 33658390 | Yue-Aging | TIMP1 | 0.00144 |
| PMID : 33658390 | Yue-Aging | IGHG1 | 1.33E-05 |
| PMID : 33658390 | Yue-Aging | PIGR | -0.00015 |
| PMID : 33658390 | Yue-Aging | CXCL1 | -0.0002 |
| PMID : 33658390 | Yue-Aging | CCL28 | -0.00104 |
| PMID : 33658390 | Yue-Aging | RPN2 | -0.00195 |
| PMID : 33658390 | Yue-Aging | CCNB1 | -0.00482 |
| PMID : 33658390 | Yue-Aging | MPC1 | -0.00826 |
| PMID : 33658390 | Yue-Aging | PLK1 | -0.013 |
| PMID : 33376727 | Yue-BRI | SLC1A5 | 0.6579 |
| PMID : 33376727 | Yue-BRI | INPP4B | 0.3337 |
| PMID : 33376727 | Yue-BRI | SRC | -0.735 |
| PMID : 33376727 | Yue-BRI | CCNE1 | -0.7475 |
| PMID : 33376727 | Yue-BRI | RPS6KA1 | -0.8763 |
| PMID : 33376727 | Yue-BRI | EGFR | 0.8318 |
| PMID : 32605475 | Zhang-EBM | YTHDC2 | -0.24745 |
| PMID : 32605475 | Zhang-EBM | IGF2BP3 | 0.153018 |
| PMID : 31796117 | Zhang-JTM | PSPC1 | 1.146 |
| PMID : 31796117 | Zhang-JTM | HSP90B1 | 1.093 |
| PMID : 31796117 | Zhang-JTM | PHF20L1 | 1.065 |
| PMID : 31796117 | Zhang-JTM | DENND5B | 1.038 |
| PMID : 31796117 | Zhang-JTM | BEX4 | 0.711 |
| PMID : 31796117 | Zhang-JTM | LPGAT1 | 0.705 |
| PMID : 31796117 | Zhang-JTM | DDIT4 | 0.672 |
| PMID : 31796117 | Zhang-JTM | KLK10 | 0.446 |

| | | | |
|---|---|---|---|
| PMID : 31796117 | Zhang-JTM | HOXC6 | 0.249 |
| PMID : 31796117 | Zhang-JTM | ZFPM2 | -0.69 |
| PMID : 31796117 | Zhang-JTM | VEGFA | -0.834 |
| PMID : 31796117 | Zhang-JTM | SHOC2 | -0.904 |
| PMID : 31796117 | Zhang-JTM | FASLG | -1.243 |
| PMID : 31796117 | Zhang-JTM | MFN2 | -1.489 |
| PMID : 31796117 | Zhang-JTM | ENDOU | -4.273 |
| PMID : 32569190 | Zhang-Med | THBS1 | 0.2989 |
| PMID : 32569190 | Zhang-Med | FGF11 | 0.3787 |
| PMID : 32569190 | Zhang-Med | CRP | 0.2587 |
| PMID : 32569190 | Zhang-Med | PROK1 | 0.2304 |
| PMID : 32569190 | Zhang-Med | S100A14 | -0.1192 |
| PMID : 32569190 | Zhang-Med | CCL19 | -0.335 |
| PMID : 31552190 | Zhou2019 | GNAI3 | 1.067602 |
| PMID : 31552190 | Zhou2019 | DNAJB9 | 0.826469 |
| PMID : 31552190 | Zhou2019 | CAPN10 | 0.819951 |
| PMID : 31552190 | Zhou2019 | PPP1R15A | 0.217835 |
| PMID : 31552190 | Zhou2019 | DAPK2 | -0.0392 |
| PMID : 33102231 | Zhou2020 | FLT3 | 1.06119 |
| PMID : 33102231 | Zhou2020 | CD36 | 0.64973 |
| PMID : 33102231 | Zhou2020 | ITGA3 | 0.50566 |
| PMID : 33102231 | Zhou2020 | CR2 | 0.43809 |
| PMID : 33102231 | Zhou2020 | CD2 | 0.43412 |
| PMID : 33102231 | Zhou2020 | IL7 | 0.24174 |
| PMID : 33102231 | Zhou2020 | CD55 | 0.03472 |
| PMID : 33747908 | Zhu2021 | ENO3 | 0.3989 |
| PMID : 33747908 | Zhu2021 | SPAG4 | 0.1174 |
| PMID : 33747908 | Zhu2021 | STC2 | 0.0442 |
| PMID : 33747908 | Zhu2021 | GPC1 | 0.02693 |
| PMID : 33747908 | Zhu2021 | P4HA1 | 0.0239 |
| PMID : 31572060 | Zou | ORAI3 | 0.112 |
| PMID : 31572060 | Zou | FBP1 | 0.082 |
| PMID : 31572060 | Zou | VEGFA | 0.0733 |
| PMID : 31572060 | Zou | MVD | 0.043 |
| PMID : 31572060 | Zou | GAS6 | 0.014 |
| PMID : 31572060 | Zou | CYB5R3 | -0.003 |
| PMID : 31572060 | Zou | ZBTB44 | -0.003 |
| PMID : 31572060 | Zou | MDM2 | -0.013 |
| PMID : 31572060 | Zou | PRELID2 | -0.025 |
| PMID : 31572060 | Zou | CCNG1 | -0.026 |
| PMID : 31572060 | Zou | FAM117B | -0.032 |
| PMID : 31572060 | Zou | CASP6 | -0.045 |
| PMID : 31572060 | Zou | TRAF3 | -0.06 |
| PMID : 31572060 | Zou | RRP1B | -0.129 |

**Table S5. Contingency Table of CRPS Risk Status by Microsatellite Stability (Count of Patients)**

| | CRPS Low Risk | CRPS High Risk |
|---|---|---|
| pMMR | 242 | 258 |
| dMMR | 49 | 32 |
| Total | 291 | 290 |

p = 0.032, Statistical significance was calculated Chi-square test

**Table S6. Reagents and antibodies**

| Antibody name | Source | Identifier | Application |
|---|---|---|---|
| Ki67 | abcam | ab15580 | IHC (Human and Mouse) |
| panCK | abcam | ab7753 | IHC/IF (Human and Mouse) |
| HSP27 | CST | #50353 | WB/IHC/IF (Human) |
| Vimentin | CST | #5741 | WB/IHC/IF (Human and Mouse) |
| β-Tubulin | ABclonal | AC030 | WB (Human) |
| FAPα | abcam | ab207178 | WB/IHC/IF (Human) |
| PDGFRβ | abcam | ab69506 | WB (Human and Mouse) |
| ZEB1 | CST | #3396 | WB (Human) |
| N-Cadherin | CST | #13116 | WB (Human) |
| E-Cadherin | CST | #3195 | WB (Human) |
| Snail | CST | #3879 | WB (Human) |

**Table S7. Differentially expressed genes of CAF subtypes in RJ-SC-Cohort**

| p_val | avg_log2FC | pct.1 | pct.2 | p_val_adj | cluster | gene |
|---|---|---|---|---|---|---|
| 5.2975E-197 | 2.35629433 | 0.815 | 0.353 | 1.3277E-192 | CAF-A | RARRES2 |
| 1.7573E-189 | 2.447199888 | 0.737 | 0.182 | 4.4042E-185 | CAF-A | MMP2 |
| 5.2022E-184 | 2.689875065 | 0.73 | 0.193 | 1.3038E-179 | CAF-A | DCN |
| 8.0152E-179 | 2.903871188 | 0.709 | 0.183 | 2.0088E-174 | CAF-A | LUM |
| 1.8332E-163 | 3.314509166 | 0.721 | 0.212 | 4.5943E-159 | CAF-A | CXCL14 |
| 4.476E-163 | 1.836527126 | 0.805 | 0.403 | 1.1218E-158 | CAF-A | C1S |
| 1.011E-162 | 2.008030292 | 0.758 | 0.281 | 2.5337E-158 | CAF-A | TMEM176B |
| 9.7426E-154 | 2.011915054 | 0.69 | 0.214 | 2.4417E-149 | CAF-A | CTSK |
| 5.4384E-148 | 2.323705843 | 0.673 | 0.193 | 1.363E-143 | CAF-A | FBLN1 |
| 2.0927E-145 | 2.004375487 | 0.6 | 0.094 | 5.2447E-141 | CAF-A | PDGFRA |
| 8.1648E-145 | 1.784805394 | 0.703 | 0.22 | 2.0463E-140 | CAF-A | TMEM176A |
| 5.8539E-143 | 1.953738723 | 0.647 | 0.162 | 1.4671E-138 | CAF-A | VCAN |
| 1.3807E-136 | 1.790253564 | 0.668 | 0.168 | 3.4604E-132 | CAF-A | MFAP4 |
| 1.4702E-131 | 1.655856592 | 0.723 | 0.363 | 3.6846E-127 | CAF-A | NBL1 |
| 8.4329E-126 | 1.72853552 | 0.856 | 0.53 | 2.1135E-121 | CAF-A | COL1A2 |
| 4.1031E-125 | 1.482768344 | 0.518 | 0.069 | 1.0283E-120 | CAF-A | PDPN |
| 3.802E-123 | 1.749346701 | 0.685 | 0.25 | 9.5287E-119 | CAF-A | SERPINF1 |
| 3.7822E-121 | 2.340749073 | 0.74 | 0.341 | 9.479E-117 | CAF-A | IGFBP5 |
| 7.6327E-119 | 1.410910457 | 0.689 | 0.294 | 1.9129E-114 | CAF-A | EMILIN1 |
| 1.0705E-116 | 1.302985564 | 0.848 | 0.503 | 2.6828E-112 | CAF-A | COL6A2 |
| 1.1043E-115 | 1.666695125 | 0.851 | 0.525 | 2.7677E-111 | CAF-A | COL3A1 |
| 3.1884E-115 | 1.260676655 | 0.549 | 0.115 | 7.9907E-111 | CAF-A | OLFML3 |
| 7.0294E-114 | 2.652412835 | 0.546 | 0.108 | 1.7617E-109 | CAF-A | CFD |
| 7.3918E-109 | 1.656952131 | 0.551 | 0.141 | 1.8525E-104 | CAF-A | MFAP2 |
| 7.7697E-108 | 1.368320086 | 0.736 | 0.372 | 1.9472E-103 | CAF-A | NNMT |
| 1.281E-107 | 1.611161813 | 0.748 | 0.352 | 3.2105E-103 | CAF-A | COL6A3 |
| 2.6788E-106 | 1.275706416 | 0.663 | 0.252 | 6.7136E-102 | CAF-A | LRP1 |
| 3.6152E-106 | 1.372128902 | 0.72 | 0.352 | 9.0603E-102 | CAF-A | C1R |
| 4.633E-104 | 1.281553291 | 0.791 | 0.439 | 1.1611E-99 | CAF-A | COL6A1 |
| 1.1676E-102 | 1.429388835 | 0.87 | 0.629 | 2.92636E-98 | CAF-A | TIMP1 |
| 2.23896E-94 | 0.973007158 | 0.853 | 0.569 | 5.61128E-90 | CAF-A | CST3 |
| 2.81802E-94 | 2.271074113 | 0.499 | 0.126 | 7.06252E-90 | CAF-A | POSTN |
| 4.90025E-94 | 1.7847605 | 0.851 | 0.531 | 1.2281E-89 | CAF-A | COL1A1 |
| 3.93877E-92 | 1.753850398 | 0.706 | 0.363 | 9.87134E-88 | CAF-A | CTGF |
| 1.09571E-91 | 1.210554702 | 0.689 | 0.364 | 2.74607E-87 | CAF-A | S100A13 |
| 1.94697E-91 | 1.362609865 | 0.676 | 0.342 | 4.87951E-87 | CAF-A | PCOLCE |
| 8.60628E-90 | 0.93924075 | 0.467 | 0.093 | 2.15691E-85 | CAF-A | FENDRR |

Statistical significance was calculated using Wilcoxon test
n = 2,276 in CAF-A group
n = 305 in CAF-B group
n = 412 in CAF-C group
n = 255 in CAF-D group

| | | | | | | |
|---|---|---|---|---|---|---|
| 1.46473E-88 | 1.354860995 | 0.554 | 0.18 | 3.67091E-84 | CAF-A | PLTP |
| 3.49145E-88 | 1.038274095 | 0.793 | 0.468 | 8.75027E-84 | CAF-A | SELENOM |
| 8.41111E-88 | 1.047004466 | 0.451 | 0.091 | 2.10799E-83 | CAF-A | BMP4 |
| 1.29686E-87 | 1.074276126 | 0.729 | 0.372 | 3.25018E-83 | CAF-A | AEBP1 |
| 1.33512E-87 | 1.310695062 | 0.49 | 0.12 | 3.34609E-83 | CAF-A | MMP19 |
| 6.03079E-87 | 0.876397678 | 0.436 | 0.074 | 1.51144E-82 | CAF-A | FBLN5 |
| 7.23143E-87 | 1.083710609 | 0.568 | 0.17 | 1.81234E-82 | CAF-A | MEG3 |
| 1.61213E-86 | 1.043504441 | 0.592 | 0.235 | 4.04033E-82 | CAF-A | CDH11 |
| 1.73344E-86 | 1.066423923 | 0.39 | 0.046 | 4.34435E-82 | CAF-A | ALDH1A3 |
| 2.11658E-86 | 0.907469955 | 0.487 | 0.121 | 5.30457E-82 | CAF-A | MRC2 |
| 1.13034E-84 | 0.829931775 | 0.44 | 0.076 | 2.83286E-80 | CAF-A | PLXDC2 |
| 1.491E-84 | 0.768827146 | 0.409 | 0.061 | 3.73674E-80 | CAF-A | CLMP |
| 2.04611E-83 | 1.425596493 | 0.522 | 0.163 | 5.12796E-79 | CAF-A | ISLR |
| 3.19123E-83 | 0.729991914 | 0.357 | 0.03 | 7.99786E-79 | CAF-A | LSAMP |
| 1.57165E-82 | 1.395387676 | 0.572 | 0.226 | 3.93888E-78 | CAF-A | TCF21 |
| 3.44607E-82 | 1.098520192 | 0.635 | 0.278 | 8.63655E-78 | CAF-A | FHL2 |
| 1.1634E-81 | 1.40972779 | 0.584 | 0.235 | 2.91572E-77 | CAF-A | SULF1 |
| 3.65142E-81 | 0.978171589 | 0.603 | 0.262 | 9.15119E-77 | CAF-A | CLEC11A |
| 3.94643E-81 | 0.809434883 | 0.489 | 0.136 | 9.89055E-77 | CAF-A | PDLIM4 |
| 1.24264E-80 | 1.097317476 | 0.416 | 0.081 | 3.11431E-76 | CAF-A | TMEM158 |
| 1.45574E-80 | 1.112162321 | 0.507 | 0.157 | 3.64838E-76 | CAF-A | PROCR |
| 4.13422E-80 | 0.83430128 | 0.402 | 0.068 | 1.03612E-75 | CAF-A | COL16A1 |
| 4.62492E-80 | 1.099259301 | 0.421 | 0.078 | 1.1591E-75 | CAF-A | TMEM119 |
| 1.76213E-79 | 0.849111484 | 0.431 | 0.093 | 4.41625E-75 | CAF-A | PITX1 |
| 1.18433E-78 | 1.04607908 | 0.69 | 0.366 | 2.96816E-74 | CAF-A | TIMP2 |
| 1.82019E-77 | 1.592142575 | 0.578 | 0.246 | 4.56176E-73 | CAF-A | PLAT |
| 2.24775E-77 | 1.506072228 | 0.415 | 0.083 | 5.63332E-73 | CAF-A | COL8A1 |
| 3.66208E-76 | 1.552227857 | 0.467 | 0.133 | 9.1779E-72 | CAF-A | EFEMP1 |
| 5.49873E-76 | 1.199949645 | 0.699 | 0.396 | 1.37809E-71 | CAF-A | LTBP4 |
| 1.92241E-75 | 0.991935332 | 0.642 | 0.302 | 4.81793E-71 | CAF-A | NUPR1 |
| 8.38319E-74 | 0.924535104 | 0.639 | 0.315 | 2.10099E-69 | CAF-A | EFEMP2 |
| 2.44139E-73 | 1.456771979 | 0.656 | 0.324 | 6.11862E-69 | CAF-A | CYR61 |
| 1.57069E-72 | 2.170414982 | 0.388 | 0.071 | 3.93647E-68 | CAF-A | PTGDS |
| 1.6743E-72 | 0.813529318 | 0.852 | 0.776 | 4.19613E-68 | CAF-A | CD63 |
| 2.21002E-72 | 1.113706155 | 0.659 | 0.331 | 5.53875E-68 | CAF-A | COL5A1 |
| 7.32596E-72 | 0.743279443 | 0.455 | 0.128 | 1.83603E-67 | CAF-A | GLT8D2 |
| 8.99993E-72 | 1.738658808 | 0.391 | 0.084 | 2.25556E-67 | CAF-A | CTHRC1 |
| 1.04071E-71 | 0.683684949 | 0.97 | 0.968 | 2.60823E-67 | CAF-A | FTL |

| | | | | | | |
|---|---|---|---|---|---|---|
| 1.66143E-71 | 2.23366943 | 0.369 | 0.062 | 4.16388E-67 | CAF-A | APOD |
| 1.79031E-71 | 1.186743902 | 0.442 | 0.121 | 4.48688E-67 | CAF-A | EMID1 |
| 1.87864E-70 | 0.980121967 | 0.519 | 0.181 | 4.70824E-66 | CAF-A | MXRA5 |
| 3.44708E-70 | 0.786474499 | 0.363 | 0.059 | 8.63908E-66 | CAF-A | SPON1 |
| 4.22017E-70 | 1.053209746 | 0.615 | 0.271 | 1.05766E-65 | CAF-A | CFH |
| 2.04092E-69 | 0.801102422 | 0.46 | 0.126 | 5.11496E-65 | CAF-A | DPYSL3 |
| 2.72051E-69 | 1.800292912 | 0.488 | 0.182 | 6.81815E-65 | CAF-A | SERPINE2 |
| 1.03706E-68 | 0.70960928 | 0.351 | 0.052 | 2.59908E-64 | CAF-A | SAMD11 |
| 9.45453E-67 | 0.944595268 | 0.392 | 0.088 | 2.36949E-62 | CAF-A | LINC01082 |
| 9.56259E-67 | 0.682079375 | 0.339 | 0.048 | 2.39658E-62 | CAF-A | FIBIN |
| 1.25378E-66 | 0.750939876 | 0.517 | 0.189 | 3.14223E-62 | CAF-A | ALDH2 |
| 1.44053E-66 | 0.667840973 | 0.33 | 0.044 | 3.61026E-62 | CAF-A | CREB3L1 |
| 5.68895E-66 | 0.641955987 | 0.335 | 0.049 | 1.42576E-61 | CAF-A | SRPX2 |
| 1.06903E-65 | 1.147899403 | 0.58 | 0.258 | 2.67919E-61 | CAF-A | ANTXR1 |
| 2.40743E-65 | 0.841141759 | 0.368 | 0.074 | 6.0335E-61 | CAF-A | LAMA2 |
| 4.61393E-65 | 0.701482001 | 0.866 | 0.846 | 1.15634E-60 | CAF-A | PPIB |
| 1.9274E-64 | 0.780708846 | 0.339 | 0.055 | 4.83046E-60 | CAF-A | PODN |
| 3.7844E-64 | 0.967297542 | 0.537 | 0.23 | 9.48446E-60 | CAF-A | FBN1 |
| 8.99709E-64 | 0.8677858 | 0.986 | 0.993 | 2.25485E-59 | CAF-A | FTH1 |
| 1.29147E-63 | 0.670277755 | 0.32 | 0.043 | 3.23669E-59 | CAF-A | PCDH7 |
| 2.56901E-63 | 0.786295743 | 0.424 | 0.122 | 6.43845E-59 | CAF-A | PRKAR2B |
| 5.84067E-63 | 0.865953467 | 0.34 | 0.058 | 1.46379E-58 | CAF-A | FGF7 |
| 1.22191E-62 | 1.22589568 | 0.496 | 0.2 | 3.06234E-58 | CAF-A | IGFBP6 |
| 4.53629E-62 | 1.114611096 | 0.667 | 0.395 | 1.13688E-57 | CAF-A | SPON2 |
| 7.68715E-62 | 0.804651937 | 0.304 | 0.039 | 1.92655E-57 | CAF-A | WNT5A |
| 1.24088E-61 | 0.69815679 | 0.514 | 0.205 | 3.1099E-57 | CAF-A | TMEM98 |
| 1.6945E-61 | 0.673402363 | 0.441 | 0.138 | 4.24676E-57 | CAF-A | FGFR1 |
| 3.42011E-61 | 0.629897339 | 0.339 | 0.061 | 8.57148E-57 | CAF-A | SGCD |
| 1.05572E-60 | 0.776206688 | 0.57 | 0.293 | 2.64584E-56 | CAF-A | PRNP |
| 1.77047E-60 | 0.865811459 | 0.623 | 0.299 | 4.43715E-56 | CAF-A | LAMB1 |
| 2.90147E-60 | 0.870436534 | 0.514 | 0.21 | 7.27166E-56 | CAF-A | ECM1 |
| 3.27273E-60 | 0.693154429 | 0.467 | 0.169 | 8.20211E-56 | CAF-A | WLS |
| 4.92748E-60 | 1.544246051 | 0.429 | 0.141 | 1.23493E-55 | CAF-A | F3 |
| 1.15604E-59 | 0.86215455 | 0.66 | 0.355 | 2.89728E-55 | CAF-A | PMP22 |
| 1.18889E-59 | 0.715987176 | 0.601 | 0.299 | 2.97959E-55 | CAF-A | FCGRT |
| 1.35554E-59 | 0.806837405 | 0.642 | 0.376 | 3.39726E-55 | CAF-A | NFIC |
| 1.80041E-59 | 1.128666309 | 0.653 | 0.354 | 4.51219E-55 | CAF-A | PALLD |
| 6.70625E-59 | 0.981991622 | 0.541 | 0.253 | 1.68072E-54 | CAF-A | MMP14 |

| | | | | | | |
|---|---|---|---|---|---|---|
| 2.58719E-58 | 0.665549304 | 0.518 | 0.214 | 6.48402E-54 | CAF-A | NDN |
| 2.99708E-58 | 0.675890161 | 0.321 | 0.053 | 7.51129E-54 | CAF-A | ITGA11 |
| 3.79104E-58 | 0.806566163 | 0.676 | 0.399 | 9.50111E-54 | CAF-A | BEX3 |
| 4.68124E-58 | 0.823946414 | 0.759 | 0.471 | 1.17321E-53 | CAF-A | SERPING1 |
| 7.50462E-58 | 2.304673171 | 0.332 | 0.071 | 1.88081E-53 | CAF-A | CXCL1 |
| 1.55543E-57 | 2.363096496 | 0.362 | 0.091 | 3.89821E-53 | CAF-A | ADAMDEC1 |
| 1.96599E-57 | 1.698361122 | 0.649 | 0.433 | 4.92717E-53 | CAF-A | SOD2 |
| 2.5345E-57 | 0.782932576 | 0.91 | 0.835 | 6.35196E-53 | CAF-A | LGALS1 |
| 2.70396E-57 | 0.784461236 | 0.352 | 0.078 | 6.77666E-53 | CAF-A | HTRA3 |
| 5.61811E-57 | 0.83713257 | 0.371 | 0.095 | 1.40801E-52 | CAF-A | ITIH5 |
| 2.48376E-56 | 0.745120689 | 0.565 | 0.279 | 6.22481E-52 | CAF-A | FKBP10 |
| 3.3715E-56 | 0.784471175 | 0.39 | 0.114 | 8.44966E-52 | CAF-A | SMPDL3A |
| 3.63865E-56 | 0.715897562 | 0.479 | 0.179 | 9.11918E-52 | CAF-A | BASP1 |
| 5.66476E-56 | 1.253079461 | 0.335 | 0.074 | 1.4197E-51 | CAF-A | THBS2 |
| 8.72722E-56 | 0.777792189 | 0.496 | 0.217 | 2.18722E-51 | CAF-A | CERCAM |
| 1.44023E-55 | 0.737544485 | 0.353 | 0.083 | 3.6095E-51 | CAF-A | PTGER2 |
| 1.52819E-55 | 0.684344448 | 0.499 | 0.206 | 3.82996E-51 | CAF-A | RHOBTB3 |
| 1.60297E-55 | 0.706059576 | 0.942 | 0.914 | 4.01736E-51 | CAF-A | S100A6 |
| 1.80792E-55 | 1.154935708 | 0.627 | 0.351 | 4.53101E-51 | CAF-A | TGFBI |
| 3.43891E-55 | 0.93742507 | 0.433 | 0.148 | 8.61859E-51 | CAF-A | EDNRB |
| 3.984E-55 | 0.619311576 | 0.344 | 0.073 | 9.9847E-51 | CAF-A | GJA1 |
| 5.40788E-55 | 0.664378205 | 0.582 | 0.294 | 1.35532E-50 | CAF-A | SCARB2 |
| 7.00619E-55 | 0.747615434 | 0.395 | 0.119 | 1.75589E-50 | CAF-A | ANGPTL2 |
| 9.13008E-55 | 0.981154575 | 0.79 | 0.574 | 2.28818E-50 | CAF-A | CEBPD |
| 6.79666E-54 | 0.650680632 | 0.304 | 0.056 | 1.70338E-49 | CAF-A | MMP23B |
| 8.56881E-54 | 0.866162813 | 0.505 | 0.246 | 2.14752E-49 | CAF-A | QSOX1 |
| 9.53878E-54 | 0.719040046 | 0.31 | 0.061 | 2.39061E-49 | CAF-A | TSHZ2 |
| 6.39724E-53 | 0.702106554 | 0.707 | 0.483 | 1.60328E-48 | CAF-A | RRBP1 |
| 8.04015E-53 | 0.650146359 | 0.384 | 0.114 | 2.01502E-48 | CAF-A | SULF2 |
| 1.28973E-52 | 0.823485066 | 0.617 | 0.334 | 3.23233E-48 | CAF-A | MXRA8 |
| 1.439E-52 | 0.908974006 | 0.562 | 0.274 | 3.60643E-48 | CAF-A | HTRA1 |
| 2.10971E-52 | 0.615933666 | 0.304 | 0.057 | 5.28735E-48 | CAF-A | WNT2B |
| 6.82437E-52 | 0.737876022 | 0.467 | 0.187 | 1.71032E-47 | CAF-A | PCDH18 |
| 2.14271E-51 | 0.688309769 | 0.551 | 0.244 | 5.37005E-47 | CAF-A | CYGB |
| 2.14485E-51 | 0.925634077 | 0.471 | 0.201 | 5.37541E-47 | CAF-A | RBP1 |
| 2.21966E-51 | 1.901872006 | 0.3 | 0.061 | 5.5629E-47 | CAF-A | ADH1B |
| 4.22936E-51 | 0.974480531 | 0.504 | 0.228 | 1.05996E-46 | CAF-A | MDK |
| 5.98962E-51 | 1.625975825 | 0.337 | 0.081 | 1.50112E-46 | CAF-A | C3 |

| | | | | | | |
|---|---|---|---|---|---|---|
| 1.80245E-50 | 0.877343575 | 0.545 | 0.281 | 4.5173E-46 | CAF-A | PPIC |
| 2.00478E-50 | 0.757109509 | 0.705 | 0.454 | 5.02439E-46 | CAF-A | LGALS3BP |
| 4.884E-50 | 1.12072802 | 0.496 | 0.218 | 1.22403E-45 | CAF-A | CCDC80 |
| 5.51865E-50 | 0.695649229 | 0.347 | 0.094 | 1.38308E-45 | CAF-A | PTCH1 |
| 1.1255E-49 | 0.727048932 | 0.582 | 0.335 | 2.82074E-45 | CAF-A | TSPAN4 |
| 4.82329E-49 | 1.291048169 | 0.292 | 0.061 | 1.20881E-44 | CAF-A | AGT |
| 1.56115E-48 | 0.775559489 | 0.68 | 0.393 | 3.91255E-44 | CAF-A | FSTL1 |
| 2.94448E-48 | 0.673378241 | 0.46 | 0.194 | 7.37945E-44 | CAF-A | AC245595.1 |
| 3.94541E-48 | 0.868246385 | 0.302 | 0.065 | 9.88799E-44 | CAF-A | MATN2 |
| 1.32512E-47 | 0.778886064 | 0.725 | 0.574 | 3.32102E-43 | CAF-A | LAPTM4A |
| 1.39554E-47 | 0.695512535 | 0.241 | 0.029 | 3.49749E-43 | CAF-A | CHL1 |
| 3.203E-47 | 0.985681003 | 0.269 | 0.048 | 8.02736E-43 | CAF-A | TRPA1 |
| 3.79157E-47 | 0.762269873 | 0.649 | 0.452 | 9.50244E-43 | CAF-A | NPC2 |
| 4.93006E-47 | 0.628997817 | 0.507 | 0.238 | 1.23557E-42 | CAF-A | FAM114A1 |
| 9.45487E-47 | 0.783266691 | 0.565 | 0.296 | 2.36958E-42 | CAF-A | FHL1 |
| 9.75031E-47 | 0.852553226 | 0.529 | 0.257 | 2.44362E-42 | CAF-A | EDIL3 |
| 9.78761E-47 | 2.094394158 | 0.409 | 0.162 | 2.45297E-42 | CAF-A | APOE |
| 9.88555E-47 | 2.553053246 | 0.286 | 0.062 | 2.47752E-42 | CAF-A | CCL11 |
| 2.24349E-46 | 0.953694379 | 0.269 | 0.048 | 5.62264E-42 | CAF-A | RARRES1 |
| 7.41142E-46 | 0.968192276 | 0.251 | 0.038 | 1.85745E-41 | CAF-A | SCARA5 |
| 1.25086E-45 | 0.674037588 | 0.461 | 0.204 | 3.1349E-41 | CAF-A | RCN3 |
| 3.99919E-44 | 0.665141711 | 0.415 | 0.169 | 1.00228E-39 | CAF-A | PHLDA3 |
| 4.45463E-44 | 3.171728973 | 0.229 | 0.03 | 1.11642E-39 | CAF-A | CST1 |
| 5.42392E-44 | 1.151605453 | 0.514 | 0.272 | 1.35934E-39 | CAF-A | COL12A1 |
| 8.4465E-44 | 0.683848064 | 0.422 | 0.168 | 2.11686E-39 | CAF-A | SVIL |
| 8.88039E-44 | 0.808846887 | 0.367 | 0.126 | 2.2256E-39 | CAF-A | TGM2 |
| 1.52156E-43 | 1.077435372 | 0.353 | 0.115 | 3.81333E-39 | CAF-A | ADM |
| 2.74949E-43 | 0.681787835 | 0.641 | 0.352 | 6.89078E-39 | CAF-A | COL5A2 |
| 6.00095E-43 | 1.512416231 | 0.404 | 0.167 | 1.50396E-38 | CAF-A | CCL2 |
| 1.46669E-42 | 0.674063788 | 0.212 | 0.023 | 3.67583E-38 | CAF-A | WNT2 |
| 1.51915E-42 | 0.644287121 | 0.283 | 0.07 | 3.80729E-38 | CAF-A | FAP |
| 1.55671E-42 | 0.677339921 | 0.243 | 0.04 | 3.90142E-38 | CAF-A | ABI3BP |
| 3.04841E-42 | 0.658452409 | 0.814 | 0.754 | 7.63993E-38 | CAF-A | ANXA5 |
| 1.06909E-41 | 0.732519207 | 0.252 | 0.048 | 2.67934E-37 | CAF-A | ABCA8 |
| 2.94592E-41 | 0.677511614 | 0.432 | 0.19 | 7.38308E-37 | CAF-A | ENC1 |
| 7.25177E-41 | 0.787608032 | 0.289 | 0.076 | 1.81744E-36 | CAF-A | FBLN2 |
| 2.71507E-40 | 0.713612807 | 0.373 | 0.131 | 6.80451E-36 | CAF-A | ELN |
| 2.97758E-40 | 0.630145531 | 0.606 | 0.413 | 7.46242E-36 | CAF-A | PLD3 |

| | | | | | | |
|---|---|---|---|---|---|---|
| 3.82037E-40 | 0.626833429 | 0.222 | 0.033 | 9.5746E-36 | CAF-A | DMKN |
| 1.04082E-39 | 0.653320824 | 0.753 | 0.565 | 2.6085E-35 | CAF-A | CD81 |
| 1.38715E-39 | 1.732967034 | 0.244 | 0.049 | 3.47647E-35 | CAF-A | TFPI2 |
| 3.04247E-39 | 0.609976362 | 0.625 | 0.367 | 7.62505E-35 | CAF-A | TCF4 |
| 3.80047E-39 | 0.696183624 | 0.25 | 0.053 | 9.52475E-35 | CAF-A | COL7A1 |
| 7.71948E-39 | 0.635354916 | 0.233 | 0.043 | 1.93466E-34 | CAF-A | BMP5 |
| 1.19672E-36 | 0.932566041 | 0.341 | 0.133 | 2.99922E-32 | CAF-A | INHBA |
| 2.20855E-36 | 0.915944722 | 0.713 | 0.48 | 5.53506E-32 | CAF-A | IGFBP4 |
| 3.03901E-36 | 0.611491564 | 0.535 | 0.346 | 7.61637E-32 | CAF-A | PRDX4 |
| 3.1686E-36 | 0.864564696 | 0.63 | 0.388 | 7.94114E-32 | CAF-A | FN1 |
| 4.55915E-36 | 0.6123823 | 0.529 | 0.28 | 1.14261E-31 | CAF-A | RND3 |
| 8.83813E-36 | 0.962008596 | 0.204 | 0.034 | 2.21501E-31 | CAF-A | STMN2 |
| 1.23798E-35 | 0.662079291 | 0.525 | 0.285 | 3.10262E-31 | CAF-A | SOX4 |
| 5.80667E-35 | 0.687747039 | 0.556 | 0.33 | 1.45527E-30 | CAF-A | TCEAL4 |
| 2.68164E-34 | 0.671224861 | 0.788 | 0.603 | 6.72073E-30 | CAF-A | EGR1 |
| 2.87595E-34 | 0.702578722 | 0.226 | 0.048 | 7.2077E-30 | CAF-A | UBD |
| 1.48271E-33 | 0.930181933 | 0.243 | 0.062 | 3.71596E-29 | CAF-A | IGF1 |
| 2.0098E-33 | 0.731983011 | 0.654 | 0.491 | 5.03696E-29 | CAF-A | IL6ST |
| 1.39458E-32 | 0.612603376 | 0.651 | 0.467 | 3.49509E-28 | CAF-A | CALU |
| 3.00166E-32 | 1.005237295 | 0.24 | 0.068 | 7.52277E-28 | CAF-A | ADAM28 |
| 3.504E-32 | 0.869411325 | 0.231 | 0.061 | 8.78171E-28 | CAF-A | NSG1 |
| 3.60832E-32 | 0.646102478 | 0.251 | 0.072 | 9.04318E-28 | CAF-A | CXCL12 |
| 1.54891E-30 | 1.161070574 | 0.663 | 0.442 | 3.88188E-26 | CAF-A | TIMP3 |
| 2.5516E-30 | 0.98980825 | 0.207 | 0.045 | 6.39481E-26 | CAF-A | GREM1 |
| 3.95258E-29 | 0.789124479 | 0.779 | 0.717 | 9.90595E-25 | CAF-A | SQSTM1 |
| 2.5625E-28 | 0.789607201 | 0.467 | 0.288 | 6.42214E-24 | CAF-A | F2R |
| 2.51201E-25 | 0.600111551 | 0.535 | 0.358 | 6.29561E-21 | CAF-A | WARS |
| 5.08905E-24 | 0.758212434 | 0.405 | 0.217 | 1.27542E-19 | CAF-A | PLAU |
| 4.32173E-23 | 0.721403833 | 0.868 | 0.622 | 1.08311E-18 | CAF-A | SPARC |
| 1.78152E-22 | 0.640568354 | 0.292 | 0.144 | 4.46485E-18 | CAF-A | OTULINL |
| 1.07833E-21 | 0.619480121 | 0.649 | 0.44 | 2.70252E-17 | CAF-A | C11orf96 |
| 8.84317E-19 | 0.693114329 | 0.419 | 0.26 | 2.21627E-14 | CAF-A | TCIM |
| 1.67344E-18 | 0.630719193 | 0.388 | 0.231 | 4.19399E-14 | CAF-A | ICAM1 |
| 4.23512E-17 | 1.042657022 | 0.37 | 0.229 | 1.06141E-12 | CAF-A | SERPINE1 |
| 2.89567E-16 | 0.79656318 | 0.535 | 0.41 | 7.25712E-12 | CAF-A | GEM |
| 5.64622E-15 | 0.660534353 | 0.806 | 0.824 | 1.41505E-10 | CAF-A | DUSP1 |
| 7.38845E-15 | 0.642429611 | 0.538 | 0.426 | 1.85169E-10 | CAF-A | SOCS3 |
| 2.29342E-14 | 0.832553266 | 0.686 | 0.677 | 5.74776E-10 | CAF-A | CTSC |

| | | | | | | |
|---|---|---|---|---|---|---|
| 1.04957E-09 | 0.605909674 | 0.812 | 0.776 | 2.63042E-05 | CAF-A | MT-ND6 |
| 2.19235E-09 | 0.609548076 | 0.498 | 0.396 | 5.49446E-05 | CAF-A | IER3 |
| 3.39816E-06 | 0.635648233 | 0.678 | 0.779 | 0.085164571 | CAF-A | MT2A |
| 0 | 3.927950812 | 0.648 | 0.022 | 0 | CAF-C | GZMA |
| 0 | 3.447577144 | 0.9 | 0.039 | 0 | CAF-C | CD7 |
| 0 | 3.258113075 | 0.944 | 0.043 | 0 | CAF-C | CD3E |
| 0 | 3.08499477 | 0.91 | 0.063 | 0 | CAF-C | CD52 |
| 0 | 3.03540369 | 0.971 | 0.088 | 0 | CAF-C | CORO1A |
| 0 | 2.991976107 | 0.942 | 0.08 | 0 | CAF-C | RAC2 |
| 0 | 2.963240504 | 0.672 | 0.026 | 0 | CAF-C | NKG7 |
| 0 | 2.917253275 | 0.825 | 0.038 | 0 | CAF-C | PTPRCAP |
| 0 | 2.730628525 | 0.859 | 0.09 | 0 | CAF-C | CD69 |
| 0 | 2.730542248 | 0.752 | 0.041 | 0 | CAF-C | CD3D |
| 0 | 2.450944984 | 0.942 | 0.087 | 0 | CAF-C | PTPRC |
| 0 | 2.330749864 | 0.806 | 0.022 | 0 | CAF-C | CD247 |
| 0 | 2.308990688 | 0.917 | 0.079 | 0 | CAF-C | LCP1 |
| 0 | 2.245266484 | 0.879 | 0.069 | 0 | CAF-C | TRBC2 |
| 0 | 2.219050066 | 0.859 | 0.033 | 0 | CAF-C | CD2 |
| 0 | 2.180342306 | 0.796 | 0.045 | 0 | CAF-C | HCST |
| 0 | 2.178602821 | 0.699 | 0.017 | 0 | CAF-C | TRBC1 |
| 0 | 2.123483591 | 0.888 | 0.025 | 0 | CAF-C | LCK |
| 0 | 2.057655088 | 0.614 | 0.019 | 0 | CAF-C | KLRB1 |
| 0 | 2.050602493 | 0.663 | 0.031 | 0 | CAF-C | BATF |
| 0 | 2.028927173 | 0.896 | 0.083 | 0 | CAF-C | LAPTM5 |
| 0 | 2.016062837 | 0.881 | 0.053 | 0 | CAF-C | CYTIP |
| 0 | 1.927857484 | 0.871 | 0.047 | 0 | CAF-C | FYB1 |
| 0 | 1.926265299 | 0.762 | 0.058 | 0 | CAF-C | ITM2A |
| 0 | 1.799741885 | 0.83 | 0.042 | 0 | CAF-C | IL2RG |
| 0 | 1.761066251 | 0.886 | 0.082 | 0 | CAF-C | HCLS1 |
| 0 | 1.715749871 | 0.653 | 0.022 | 0 | CAF-C | CD3G |
| 0 | 1.659383823 | 0.825 | 0.081 | 0 | CAF-C | 1-Sep |
| 0 | 1.654085956 | 0.701 | 0.02 | 0 | CAF-C | IL2RB |
| 0 | 1.616131736 | 0.631 | 0.024 | 0 | CAF-C | CST7 |
| 0 | 1.566911254 | 0.857 | 0.049 | 0 | CAF-C | CD53 |
| 0 | 1.518433282 | 0.704 | 0.03 | 0 | CAF-C | LAT |
| 0 | 1.505268488 | 0.733 | 0.038 | 0 | CAF-C | SLA |
| 0 | 1.459436602 | 0.743 | 0.034 | 0 | CAF-C | SH2D2A |
| 0 | 1.434799648 | 0.728 | 0.024 | 0 | CAF-C | CD96 |

| | | | | | | |
|---|---|---|---|---|---|---|
| 0 | 1.402937895 | 0.752 | 0.025 | 0 | CAF-C | SKAP1 |
| 0 | 1.39883291 | 0.782 | 0.039 | 0 | CAF-C | ACAP1 |
| 0 | 1.388588382 | 0.721 | 0.029 | 0 | CAF-C | ARHGAP9 |
| 0 | 1.387127582 | 0.723 | 0.024 | 0 | CAF-C | TBC1D10C |
| 0 | 1.385080537 | 0.692 | 0.026 | 0 | CAF-C | PTPN7 |
| 0 | 1.380981314 | 0.803 | 0.06 | 0 | CAF-C | CD37 |
| 0 | 1.371993616 | 0.711 | 0.037 | 0 | CAF-C | SAMSN1 |
| 0 | 1.297000233 | 0.595 | 0.011 | 0 | CAF-C | SIRPG |
| 0 | 1.293053495 | 0.716 | 0.019 | 0 | CAF-C | ZAP70 |
| 0 | 1.283680408 | 0.723 | 0.04 | 0 | CAF-C | RHOH |
| 0 | 1.239014724 | 0.667 | 0.027 | 0 | CAF-C | SASH3 |
| 0 | 1.216107905 | 0.738 | 0.045 | 0 | CAF-C | PRKCH |
| 0 | 1.168129775 | 0.701 | 0.041 | 0 | CAF-C | IKZF1 |
| 0 | 1.148114784 | 0.65 | 0.023 | 0 | CAF-C | TRAF3IP3 |
| 0 | 1.128646505 | 0.667 | 0.024 | 0 | CAF-C | RASAL3 |
| 0 | 1.091672009 | 0.638 | 0.022 | 0 | CAF-C | PTPN22 |
| 0 | 1.080780953 | 0.675 | 0.029 | 0 | CAF-C | DEF6 |
| 0 | 0.979998275 | 0.602 | 0.018 | 0 | CAF-C | SPN |
| 0 | 0.940954943 | 0.595 | 0.02 | 0 | CAF-C | ARHGAP30 |
| 0 | 0.928822916 | 0.585 | 0.011 | 0 | CAF-C | DOK2 |
| 8.3726E-308 | 1.029030279 | 0.631 | 0.027 | 2.0983E-303 | CAF-C | MAP4K1 |
| 1.0652E-307 | 0.956419886 | 0.612 | 0.024 | 2.6697E-303 | CAF-C | BIN2 |
| 2.2851E-307 | 1.595547811 | 0.522 | 0.008 | 5.7269E-303 | CAF-C | TMIGD2 |
| 1.797E-305 | 4.047176498 | 0.76 | 0.07 | 4.5036E-301 | CAF-C | CCL5 |
| 2.5402E-303 | 1.19055168 | 0.624 | 0.027 | 6.3663E-299 | CAF-C | IKZF3 |
| 1.4682E-300 | 1.431239493 | 0.544 | 0.013 | 3.6796E-296 | CAF-C | PRF1 |
| 4.3503E-300 | 0.911449432 | 0.636 | 0.031 | 1.0903E-295 | CAF-C | ARHGAP45 |
| 1.2658E-298 | 0.972014183 | 0.578 | 0.02 | 3.1724E-294 | CAF-C | MYO1G |
| 3.9224E-298 | 1.297555044 | 0.51 | 0.008 | 9.8304E-294 | CAF-C | LINC01871 |
| 1.6812E-295 | 1.161063087 | 0.709 | 0.05 | 4.2134E-291 | CAF-C | DOCK8 |
| 1.7268E-294 | 1.221595932 | 0.667 | 0.041 | 4.3276E-290 | CAF-C | CD48 |
| 5.5762E-294 | 1.56468057 | 0.755 | 0.065 | 1.3975E-289 | CAF-C | UCP2 |
| 1.5172E-293 | 1.027655524 | 0.556 | 0.017 | 3.8023E-289 | CAF-C | ITGB7 |
| 4.9217E-286 | 1.094303301 | 0.66 | 0.043 | 1.2335E-281 | CAF-C | EMB |
| 1.4275E-285 | 2.490626816 | 0.964 | 0.178 | 3.5776E-281 | CAF-C | SRGN |
| 5.9655E-285 | 1.541351349 | 0.68 | 0.047 | 1.4951E-280 | CAF-C | TAGAP |
| 4.641E-284 | 1.319343695 | 0.646 | 0.038 | 1.1631E-279 | CAF-C | CENPM |
| 6.3781E-282 | 1.172416863 | 0.607 | 0.031 | 1.5985E-277 | CAF-C | GBP5 |

| | | | | | | |
|---|---|---|---|---|---|---|
| 1.3384E-281 | 2.036458018 | 0.699 | 0.053 | 3.3542E-277 | CAF-C | MKI67 |
| 3.0113E-281 | 2.240511703 | 0.803 | 0.092 | 7.5469E-277 | CAF-C | RGS1 |
| 1.0357E-278 | 1.030580119 | 0.648 | 0.041 | 2.5957E-274 | CAF-C | ABI3 |
| 3.3991E-278 | 1.638456852 | 0.687 | 0.051 | 8.5187E-274 | CAF-C | PCLAF |
| 3.9174E-278 | 1.074604588 | 0.665 | 0.046 | 9.8178E-274 | CAF-C | RHOF |
| 3.7689E-277 | 1.305865046 | 0.561 | 0.024 | 9.4456E-273 | CAF-C | CD6 |
| 4.2925E-277 | 1.117272042 | 0.602 | 0.031 | 1.0758E-272 | CAF-C | ASF1B |
| 6.9475E-277 | 0.881258137 | 0.488 | 0.009 | 1.7412E-272 | CAF-C | RGL4 |
| 2.0281E-275 | 1.190149521 | 0.529 | 0.017 | 5.0828E-271 | CAF-C | TIGIT |
| 7.608E-275 | 0.969553722 | 0.551 | 0.021 | 1.9067E-270 | CAF-C | SELPLG |
| 1.8598E-272 | 1.768634029 | 0.631 | 0.042 | 4.661E-268 | CAF-C | SPOCK2 |
| 3.636E-271 | 1.837051352 | 0.532 | 0.019 | 9.1125E-267 | CAF-C | TNFRSF18 |
| 7.4638E-268 | 0.8392095 | 0.456 | 0.006 | 1.8706E-263 | CAF-C | SLA2 |
| 1.0206E-267 | 0.880373449 | 0.563 | 0.025 | 2.5578E-263 | CAF-C | ITK |
| 2.0614E-267 | 1.095404634 | 0.575 | 0.028 | 5.1663E-263 | CAF-C | GIMAP7 |
| 5.7694E-267 | 0.949261784 | 0.602 | 0.035 | 1.4459E-262 | CAF-C | DENND2D |
| 7.895E-266 | 0.913236818 | 0.541 | 0.021 | 1.9786E-261 | CAF-C | SP140 |
| 7.0438E-262 | 0.71316068 | 0.524 | 0.019 | 1.7653E-257 | CAF-C | TMC8 |
| 1.143E-261 | 1.43743997 | 0.837 | 0.123 | 2.8645E-257 | CAF-C | STK17B |
| 7.8447E-261 | 1.123306347 | 0.461 | 0.009 | 1.966E-256 | CAF-C | CXCR6 |
| 8.2377E-259 | 0.821132166 | 0.502 | 0.017 | 2.0645E-254 | CAF-C | ITGAL |
| 2.0028E-258 | 1.186277077 | 0.704 | 0.068 | 5.0195E-254 | CAF-C | APBB1IP |
| 2.8316E-256 | 1.024870572 | 0.42 | 0.004 | 7.0966E-252 | CAF-C | GPR25 |
| 9.6937E-252 | 1.517544444 | 0.626 | 0.048 | 2.4294E-247 | CAF-C | ALOX5AP |
| 1.3136E-250 | 1.246079357 | 0.641 | 0.055 | 3.2922E-246 | CAF-C | CLEC2D |
| 8.6105E-249 | 2.167583856 | 0.731 | 0.086 | 2.158E-244 | CAF-C | TYMS |
| 1.9982E-248 | 2.481871523 | 0.981 | 0.255 | 5.0078E-244 | CAF-C | ARHGDIB |
| 2.7268E-248 | 1.681716185 | 0.485 | 0.016 | 6.8339E-244 | CAF-C | CTLA4 |
| 3.4037E-248 | 1.108322529 | 0.456 | 0.011 | 8.5304E-244 | CAF-C | ICOS |
| 2.2306E-247 | 0.974704282 | 0.563 | 0.033 | 5.5903E-243 | CAF-C | RUNX3 |
| 6.5605E-243 | 2.541186756 | 0.471 | 0.016 | 1.6442E-238 | CAF-C | GZMB |
| 9.2567E-243 | 1.194631356 | 0.799 | 0.108 | 2.3199E-238 | CAF-C | VAMP8 |
| 2.8533E-242 | 0.878074917 | 0.585 | 0.04 | 7.1509E-238 | CAF-C | TMC6 |
| 5.2708E-242 | 0.831242265 | 0.583 | 0.038 | 1.321E-237 | CAF-C | WAS |
| 1.9213E-241 | 1.045193302 | 0.697 | 0.077 | 4.8152E-237 | CAF-C | LPXN |
| 3.3092E-239 | 1.18993992 | 0.546 | 0.031 | 8.2936E-235 | CAF-C | SYTL3 |
| 8.1509E-239 | 0.827996002 | 0.566 | 0.035 | 2.0428E-234 | CAF-C | INPP5D |
| 5.0837E-238 | 1.064836837 | 0.476 | 0.017 | 1.2741E-233 | CAF-C | SIT1 |

| | | | | | | |
|---|---|---|---|---|---|---|
| 1.0017E-236 | 0.984493073 | 0.483 | 0.02 | 2.5104E-232 | CAF-C | LIME1 |
| 3.5529E-236 | 0.966571931 | 0.539 | 0.032 | 8.9043E-232 | CAF-C | CDT1 |
| 2.5711E-235 | 1.419645212 | 0.791 | 0.123 | 6.4437E-231 | CAF-C | LCP2 |
| 2.9951E-235 | 1.507782626 | 0.602 | 0.049 | 7.5064E-231 | CAF-C | DUSP4 |
| 1.1387E-233 | 0.784740452 | 0.502 | 0.024 | 2.8538E-229 | CAF-C | NUP210 |
| 1.4726E-232 | 0.99192832 | 0.534 | 0.033 | 3.6906E-228 | CAF-C | TCF19 |
| 2.0468E-232 | 0.759634437 | 0.561 | 0.036 | 5.1297E-228 | CAF-C | ARHGAP4 |
| 4.37E-232 | 0.849442459 | 0.417 | 0.008 | 1.0952E-227 | CAF-C | CXCR3 |
| 4.7514E-232 | 0.721226605 | 0.481 | 0.019 | 1.1908E-227 | CAF-C | PARVG |
| 3.6154E-231 | 1.051115891 | 0.636 | 0.061 | 9.061E-227 | CAF-C | OXNAD1 |
| 1.4867E-230 | 0.73319723 | 0.493 | 0.022 | 3.726E-226 | CAF-C | NCKAP1L |
| 5.2011E-230 | 1.088224746 | 0.583 | 0.044 | 1.3035E-225 | CAF-C | PTPN6 |
| 1.7058E-229 | 0.97117933 | 0.536 | 0.033 | 4.275E-225 | CAF-C | CLSPN |
| 2.2019E-228 | 0.85123995 | 0.437 | 0.013 | 5.5184E-224 | CAF-C | CD5 |
| 9.0716E-228 | 0.791192482 | 0.437 | 0.013 | 2.2735E-223 | CAF-C | GPR171 |
| 1.5043E-227 | 1.542068575 | 0.825 | 0.155 | 3.77E-223 | CAF-C | 6-Sep |
| 5.5445E-225 | 2.209782616 | 0.699 | 0.086 | 1.3896E-220 | CAF-C | DUSP2 |
| 5.7705E-225 | 1.023678012 | 0.595 | 0.05 | 1.4462E-220 | CAF-C | ZWINT |
| 2.647E-224 | 2.137262556 | 0.568 | 0.043 | 6.6339E-220 | CAF-C | LTB |
| 8.517E-224 | 0.831937544 | 0.413 | 0.01 | 2.1345E-219 | CAF-C | SH2D1A |
| 3.1114E-223 | 1.272470983 | 0.699 | 0.089 | 7.7977E-219 | CAF-C | LMNB1 |
| 3.8639E-221 | 1.727046872 | 0.772 | 0.132 | 9.6837E-217 | CAF-C | TRAC |
| 1.7158E-220 | 1.45567817 | 0.59 | 0.05 | 4.3002E-216 | CAF-C | NUSAP1 |
| 2.6337E-219 | 0.910405628 | 0.478 | 0.023 | 6.6006E-215 | CAF-C | KIFC1 |
| 7.3829E-219 | 0.933568363 | 0.597 | 0.056 | 1.8503E-214 | CAF-C | RASSF5 |
| 8.1789E-218 | 0.702765204 | 0.396 | 0.008 | 2.0498E-213 | CAF-C | GRAP2 |
| 3.2945E-217 | 0.897566286 | 0.425 | 0.014 | 8.2566E-213 | CAF-C | SCML4 |
| 4.1047E-217 | 0.868573803 | 0.539 | 0.038 | 1.0287E-212 | CAF-C | TACC3 |
| 6.432E-216 | 0.709663022 | 0.432 | 0.015 | 1.612E-211 | CAF-C | AC243960.1 |
| 9.0555E-216 | 1.902226038 | 0.893 | 0.246 | 2.2695E-211 | CAF-C | EVL |
| 4.2623E-215 | 0.934595168 | 0.393 | 0.009 | 1.0682E-210 | CAF-C | SLAMF1 |
| 5.2329E-215 | 1.393717599 | 0.672 | 0.08 | 1.3115E-210 | CAF-C | PTTG1 |
| 5.9809E-215 | 0.726707113 | 0.405 | 0.011 | 1.4989E-210 | CAF-C | TRAT1 |
| 3.4189E-214 | 0.703717984 | 0.49 | 0.028 | 8.5684E-210 | CAF-C | PSTPIP1 |
| 1.4983E-213 | 0.824754057 | 0.468 | 0.024 | 3.7551E-209 | CAF-C | CD38 |
| 1.3378E-212 | 0.600101621 | 0.391 | 0.009 | 3.3528E-208 | CAF-C | NLRC3 |
| 2.9547E-212 | 0.829203149 | 0.359 | 0.004 | 7.405E-208 | CAF-C | TRG-AS1 |
| 9.711E-212 | 1.275782767 | 0.663 | 0.083 | 2.4338E-207 | CAF-C | TNFRSF1B |

| | | | | | | |
|---|---|---|---|---|---|---|
| 1.5686E-211 | 0.957383623 | 0.483 | 0.027 | 3.9312E-207 | CAF-C | PKMYT1 |
| 2.2484E-211 | 0.708434976 | 0.444 | 0.019 | 5.635E-207 | CAF-C | MFNG |
| 1.2229E-208 | 0.828483453 | 0.592 | 0.057 | 3.0648E-204 | CAF-C | FERMT3 |
| 1.7772E-208 | 0.901412994 | 0.558 | 0.047 | 4.4541E-204 | CAF-C | GPRIN3 |
| 2.2017E-208 | 0.674985985 | 0.427 | 0.016 | 5.5178E-204 | CAF-C | IPCEF1 |
| 3.7313E-207 | 1.598290703 | 0.556 | 0.047 | 9.3514E-203 | CAF-C | CENPF |
| 1.4426E-205 | 1.038734628 | 0.546 | 0.047 | 3.6155E-201 | CAF-C | UHRF1 |
| 1.5134E-205 | 0.814673093 | 0.408 | 0.014 | 3.7929E-201 | CAF-C | GZMM |
| 1.9214E-205 | 0.651218255 | 0.381 | 0.009 | 4.8155E-201 | CAF-C | THEMIS |
| 5.0372E-205 | 1.661425628 | 0.512 | 0.036 | 1.2624E-200 | CAF-C | RRM2 |
| 1.0181E-204 | 2.574183569 | 0.871 | 0.237 | 2.5516E-200 | CAF-C | HMGB2 |
| 1.4196E-203 | 0.810581334 | 0.388 | 0.011 | 3.5579E-199 | CAF-C | CD28 |
| 8.8893E-202 | 0.755287956 | 0.473 | 0.028 | 2.2278E-197 | CAF-C | DOCK2 |
| 9.6952E-201 | 0.631107466 | 0.371 | 0.009 | 2.4298E-196 | CAF-C | GPR174 |
| 2.5223E-200 | 0.667949772 | 0.41 | 0.015 | 6.3213E-196 | CAF-C | GIMAP1 |
| 4.3781E-200 | 1.292448995 | 0.607 | 0.068 | 1.0972E-195 | CAF-C | TK1 |
| 3.7764E-198 | 0.70255662 | 0.364 | 0.008 | 9.4645E-194 | CAF-C | HAVCR2 |
| 3.7608E-197 | 0.771793568 | 0.439 | 0.022 | 9.4252E-193 | CAF-C | STAT4 |
| 6.6913E-197 | 0.919716908 | 0.614 | 0.075 | 1.677E-192 | CAF-C | ARRB2 |
| 1.8931E-194 | 0.721508119 | 0.434 | 0.023 | 4.7444E-190 | CAF-C | NCAPH |
| 5.2233E-194 | 1.528368646 | 0.451 | 0.027 | 1.3091E-189 | CAF-C | ASPM |
| 1.3026E-193 | 1.167488138 | 0.546 | 0.05 | 3.2646E-189 | CAF-C | ITGB2 |
| 2.9305E-193 | 1.470097025 | 0.546 | 0.051 | 7.3444E-189 | CAF-C | TOP2A |
| 1.953E-190 | 0.948242662 | 0.699 | 0.113 | 4.8945E-186 | CAF-C | OCIAD2 |
| 1.4133E-189 | 1.32771699 | 0.325 | 0.004 | 3.542E-185 | CAF-C | IFNG |
| 2.3018E-189 | 0.964354794 | 0.515 | 0.046 | 5.7688E-185 | CAF-C | MCM2 |
| 1.3827E-188 | 1.11797521 | 0.604 | 0.073 | 3.4652E-184 | CAF-C | TUBA4A |
| 9.9363E-188 | 0.900933852 | 0.549 | 0.056 | 2.4902E-183 | CAF-C | EZH2 |
| 3.7974E-187 | 2.37518496 | 0.92 | 0.344 | 9.517E-183 | CAF-C | COTL1 |
| 3.7832E-186 | 0.983224637 | 0.714 | 0.128 | 9.4816E-182 | CAF-C | ICAM3 |
| 1.1221E-185 | 0.703101272 | 0.485 | 0.038 | 2.8123E-181 | CAF-C | PREX1 |
| 4.1226E-185 | 0.768920034 | 0.33 | 0.006 | 1.0332E-180 | CAF-C | PDCD1 |
| 6.6359E-185 | 1.92167303 | 0.954 | 0.315 | 1.6631E-180 | CAF-C | LSP1 |
| 9.5259E-185 | 0.863667157 | 0.439 | 0.026 | 2.3874E-180 | CAF-C | CDCA8 |
| 1.6553E-184 | 0.732738449 | 0.391 | 0.017 | 4.1485E-180 | CAF-C | TNFRSF25 |
| 2.7247E-183 | 1.084884792 | 0.481 | 0.038 | 6.8286E-179 | CAF-C | BIRC5 |
| 4.5061E-183 | 0.68794468 | 0.456 | 0.031 | 1.1293E-178 | CAF-C | SUSD3 |
| 1.4833E-182 | 1.314943771 | 0.813 | 0.183 | 3.7174E-178 | CAF-C | CELF2 |

| | | | | | | |
|---|---|---|---|---|---|---|
| 1.2339E-181 | 0.877144318 | 0.573 | 0.068 | 3.0925E-177 | CAF-C | UBE2T |
| 1.2734E-181 | 0.705645845 | 0.476 | 0.036 | 3.1913E-177 | CAF-C | IL10RA |
| 1.4856E-181 | 0.71957765 | 0.604 | 0.074 | 3.7231E-177 | CAF-C | CYFIP2 |
| 2.1293E-181 | 0.628027956 | 0.468 | 0.033 | 5.3363E-177 | CAF-C | PPP1R16B |
| 8.7895E-181 | 1.276419437 | 0.549 | 0.058 | 2.2028E-176 | CAF-C | TPX2 |
| 1.3592E-180 | 0.721362887 | 0.464 | 0.034 | 3.4065E-176 | CAF-C | FANCI |
| 2.5686E-180 | 0.806109419 | 0.34 | 0.009 | 6.4373E-176 | CAF-C | MATK |
| 1.6679E-179 | 1.492865443 | 0.896 | 0.262 | 4.1802E-175 | CAF-C | GMFG |
| 1.877E-178 | 0.693246111 | 0.454 | 0.033 | 4.7042E-174 | CAF-C | GPR65 |
| 2.1904E-178 | 0.885356611 | 0.405 | 0.022 | 5.4897E-174 | CAF-C | DLGAP5 |
| 5.9805E-178 | 0.801473801 | 0.408 | 0.022 | 1.4988E-173 | CAF-C | GTSE1 |
| 1.0635E-177 | 0.649612606 | 0.408 | 0.023 | 2.6652E-173 | CAF-C | IL12RB1 |
| 4.3313E-177 | 1.018075145 | 0.303 | 0.004 | 1.0855E-172 | CAF-C | CTSW |
| 4.8143E-177 | 0.89107449 | 0.532 | 0.056 | 1.2066E-172 | CAF-C | MAD2L1 |
| 1.0669E-176 | 1.295303438 | 0.847 | 0.22 | 2.6738E-172 | CAF-C | FYN |
| 1.1972E-174 | 0.672826785 | 0.483 | 0.041 | 3.0003E-170 | CAF-C | CENPU |
| 1.42E-174 | 0.831709918 | 0.517 | 0.053 | 3.5587E-170 | CAF-C | CENPK |
| 4.6692E-171 | 0.714539903 | 0.408 | 0.025 | 1.1702E-166 | CAF-C | CDCA5 |
| 5.612E-171 | 1.036194062 | 0.466 | 0.039 | 1.4065E-166 | CAF-C | CCNA2 |
| 6.9314E-171 | 0.646102892 | 0.367 | 0.016 | 1.7371E-166 | CAF-C | CKAP2L |
| 9.4642E-171 | 0.999278467 | 0.493 | 0.047 | 2.3719E-166 | CAF-C | CDK1 |
| 1.5144E-170 | 0.888158758 | 0.507 | 0.052 | 3.7955E-166 | CAF-C | KIF20B |
| 5.2485E-170 | 0.755514364 | 0.583 | 0.08 | 1.3154E-165 | CAF-C | ATP6V0E2 |
| 5.5561E-170 | 0.7658747 | 0.43 | 0.03 | 1.3925E-165 | CAF-C | KNL1 |
| 1.9735E-169 | 1.154455214 | 0.767 | 0.178 | 4.946E-165 | CAF-C | GPSM3 |
| 3.4476E-167 | 1.453785473 | 0.801 | 0.215 | 8.6405E-163 | CAF-C | LIMD2 |
| 4.3388E-167 | 0.706644381 | 0.468 | 0.042 | 1.0874E-162 | CAF-C | TRAF1 |
| 7.9091E-167 | 1.072223701 | 0.621 | 0.101 | 1.9822E-162 | CAF-C | APOBEC3G |
| 9.6756E-167 | 1.652071769 | 0.835 | 0.28 | 2.4249E-162 | CAF-C | CKLF |
| 1.3705E-166 | 0.919187476 | 0.58 | 0.083 | 3.4347E-162 | CAF-C | FEN1 |
| 2.0416E-166 | 0.813848013 | 0.544 | 0.066 | 5.1166E-162 | CAF-C | IL16 |
| 2.8747E-166 | 0.626928359 | 0.379 | 0.02 | 7.2046E-162 | CAF-C | IL21R |
| 1.1154E-165 | 0.847224863 | 0.602 | 0.087 | 2.7955E-161 | CAF-C | AKNA |
| 1.8403E-165 | 0.957771662 | 0.58 | 0.08 | 4.6122E-161 | CAF-C | ATAD2 |
| 2.1015E-164 | 0.935365531 | 0.532 | 0.063 | 5.2667E-160 | CAF-C | CDKN3 |
| 1.3318E-163 | 1.086106265 | 0.777 | 0.196 | 3.3378E-159 | CAF-C | CSK |
| 1.6167E-163 | 0.656293192 | 0.364 | 0.018 | 4.0519E-159 | CAF-C | CDCA2 |
| 4.4078E-163 | 0.769026955 | 0.546 | 0.072 | 1.1047E-158 | CAF-C | SH3BP1 |

| | | | | | | |
|---|---|---|---|---|---|---|
| 3.2971E-162 | 1.441571074 | 0.847 | 0.278 | 8.2631E-158 | CAF-C | ABRACL |
| 5.0622E-162 | 0.877411605 | 0.663 | 0.118 | 1.2687E-157 | CAF-C | ANKRD44 |
| 5.6556E-161 | 0.788143889 | 0.558 | 0.074 | 1.4174E-156 | CAF-C | DOCK10 |
| 5.8963E-160 | 0.749652425 | 0.451 | 0.041 | 1.4777E-155 | CAF-C | PBX4 |
| 3.885E-159 | 0.673811566 | 0.546 | 0.068 | 9.7365E-155 | CAF-C | SLC38A1 |
| 5.4782E-159 | 0.892652152 | 0.466 | 0.045 | 1.3729E-154 | CAF-C | GINS2 |
| 1.3727E-158 | 0.729936904 | 0.352 | 0.017 | 3.4403E-154 | CAF-C | TROAP |
| 1.4593E-158 | 2.228266749 | 0.291 | 0.006 | 3.6572E-154 | CAF-C | TRDC |
| 1.093E-157 | 0.739715929 | 0.417 | 0.031 | 2.7393E-153 | CAF-C | GIMAP4 |
| 3.8507E-157 | 0.703234566 | 0.311 | 0.01 | 9.6505E-153 | CAF-C | CCR6 |
| 3.3579E-156 | 1.123844314 | 0.284 | 0.006 | 8.4156E-152 | CAF-C | KIR2DL4 |
| 6.1504E-155 | 1.44820701 | 0.816 | 0.248 | 1.5414E-150 | CAF-C | TMPO |
| 1.1536E-154 | 1.27972715 | 0.833 | 0.261 | 2.8911E-150 | CAF-C | SLC9A3R1 |
| 2.4433E-154 | 1.305168374 | 0.566 | 0.079 | 6.1235E-150 | CAF-C | HIST1H1D |
| 5.6849E-154 | 2.281996172 | 0.964 | 0.627 | 1.4248E-149 | CAF-C | IL32 |
| 1.2728E-153 | 0.97796099 | 0.405 | 0.031 | 3.1899E-149 | CAF-C | CCNB2 |
| 4.4672E-153 | 0.654281286 | 0.396 | 0.029 | 1.1196E-148 | CAF-C | NCAPG |
| 4.8356E-153 | 0.679226639 | 0.43 | 0.039 | 1.2119E-148 | CAF-C | BRCA1 |
| 7.116E-153 | 1.19627268 | 0.833 | 0.251 | 1.7834E-148 | CAF-C | GNG2 |
| 8.94E-153 | 0.930786399 | 0.636 | 0.12 | 2.2406E-148 | CAF-C | PHF19 |
| 2.4712E-152 | 1.450444632 | 0.284 | 0.007 | 6.1933E-148 | CAF-C | LINC02446 |
| 1.3571E-151 | 2.370820881 | 0.842 | 0.321 | 3.4011E-147 | CAF-C | STMN1 |
| 1.717E-151 | 1.048259056 | 0.59 | 0.098 | 4.3032E-147 | CAF-C | MCM5 |
| 8.5025E-151 | 1.35764833 | 0.784 | 0.23 | 2.1309E-146 | CAF-C | STK17A |
| 2.1757E-150 | 1.436421473 | 0.731 | 0.183 | 5.4527E-146 | CAF-C | SMC4 |
| 5.0907E-150 | 0.833680884 | 0.328 | 0.016 | 1.2758E-145 | CAF-C | CD70 |
| 3.6868E-148 | 0.716631829 | 0.57 | 0.09 | 9.2398E-144 | CAF-C | DCK |
| 2.0396E-147 | 0.863219058 | 0.609 | 0.112 | 5.1116E-143 | CAF-C | PSMB8-AS1 |
| 6.9786E-147 | 0.762543244 | 0.609 | 0.106 | 1.749E-142 | CAF-C | CDC42SE2 |
| 2.9497E-145 | 1.004523104 | 0.583 | 0.103 | 7.3926E-141 | CAF-C | DHFR |
| 5.6993E-145 | 1.798889133 | 0.973 | 0.759 | 1.4283E-140 | CAF-C | SH3BGRL3 |
| 6.7083E-145 | 0.866856075 | 0.694 | 0.153 | 1.6812E-140 | CAF-C | FAM49B |
| 8.214E-144 | 0.824392419 | 0.609 | 0.113 | 2.0586E-139 | CAF-C | FMNL1 |
| 2.071E-143 | 0.685292841 | 0.323 | 0.016 | 5.1904E-139 | CAF-C | CENPA |
| 3.287E-143 | 1.348564576 | 0.816 | 0.282 | 8.2379E-139 | CAF-C | OSTF1 |
| 5.8108E-143 | 1.022821849 | 0.784 | 0.212 | 1.4563E-138 | CAF-C | KIAA1551 |
| 7.7258E-143 | 0.765711491 | 0.551 | 0.089 | 1.9362E-138 | CAF-C | GMNN |
| 6.1581E-142 | 0.622914147 | 0.367 | 0.026 | 1.5433E-137 | CAF-C | CEP55 |

| | | | | | | |
|---|---|---|---|---|---|---|
| 2.4682E-141 | 0.664509251 | 0.527 | 0.078 | 6.1858E-137 | CAF-C | CENPN |
| 8.4428E-141 | 0.762025785 | 0.551 | 0.087 | 2.1159E-136 | CAF-C | CAMK4 |
| 2.993E-140 | 0.944473206 | 0.624 | 0.129 | 7.5011E-136 | CAF-C | DNAJC9 |
| 3.5324E-140 | 0.77653723 | 0.371 | 0.029 | 8.853E-136 | CAF-C | CENPE |
| 2.0443E-139 | 1.019683145 | 0.837 | 0.284 | 5.1234E-135 | CAF-C | WIPF1 |
| 2.0093E-138 | 0.716043951 | 0.646 | 0.128 | 5.0358E-134 | CAF-C | TSTD1 |
| 2.6727E-138 | 0.886778319 | 0.607 | 0.118 | 6.6984E-134 | CAF-C | SMC2 |
| 1.1766E-137 | 0.883860337 | 0.529 | 0.08 | 2.9487E-133 | CAF-C | HELLS |
| 1.8199E-137 | 1.477020007 | 1 | 0.982 | 4.5609E-133 | CAF-C | TMSB4X |
| 3.8178E-137 | 1.455411084 | 0.502 | 0.07 | 9.5681E-133 | CAF-C | UBE2C |
| 5.3312E-136 | 0.637943945 | 0.299 | 0.013 | 1.3361E-131 | CAF-C | JAML |
| 8.7204E-135 | 0.686323167 | 0.345 | 0.024 | 2.1855E-130 | CAF-C | NUF2 |
| 9.9635E-133 | 1.562395008 | 0.995 | 0.921 | 2.497E-128 | CAF-C | PFN1 |
| 2.1096E-132 | 0.791222641 | 0.267 | 0.009 | 5.2871E-128 | CAF-C | KLRC3 |
| 2.2186E-132 | 0.839737486 | 0.539 | 0.087 | 5.5603E-128 | CAF-C | SLC7A5 |
| 3.9421E-132 | 0.970445946 | 0.767 | 0.203 | 9.8798E-128 | CAF-C | HLA-DPA1 |
| 2.0359E-131 | 0.691396534 | 0.405 | 0.041 | 5.1024E-127 | CAF-C | GPR183 |
| 1.3099E-130 | 0.649753156 | 0.345 | 0.025 | 3.2829E-126 | CAF-C | AURKB |
| 9.8351E-130 | 0.770644037 | 0.561 | 0.1 | 2.4649E-125 | CAF-C | ATP2A3 |
| 1.8233E-129 | 1.238379848 | 0.823 | 0.326 | 4.5696E-125 | CAF-C | BUB3 |
| 3.293E-129 | 0.844336273 | 0.721 | 0.194 | 8.253E-125 | CAF-C | STK4 |
| 1.3114E-128 | 1.185384593 | 0.68 | 0.177 | 3.2866E-124 | CAF-C | MCM7 |
| 1.9115E-128 | 0.620928974 | 0.345 | 0.026 | 4.7905E-124 | CAF-C | KIF11 |
| 2.4388E-128 | 0.702954934 | 0.519 | 0.083 | 6.1122E-124 | CAF-C | CCDC69 |
| 5.9294E-127 | 0.718055057 | 0.347 | 0.029 | 1.486E-122 | CAF-C | IKZF2 |
| 6.0883E-127 | 0.815328839 | 0.544 | 0.1 | 1.5258E-122 | CAF-C | GALM |
| 1.38E-126 | 0.978433959 | 0.362 | 0.032 | 3.4587E-122 | CAF-C | MIR155HG |
| 1.9358E-126 | 0.784664728 | 0.498 | 0.077 | 4.8516E-122 | CAF-C | MCM4 |
| 2.6495E-126 | 1.291845428 | 0.837 | 0.314 | 6.6401E-122 | CAF-C | JPT1 |
| 2.7366E-126 | 0.765206594 | 0.624 | 0.141 | 6.8584E-122 | CAF-C | TTC39C |
| 1.1086E-125 | 1.3497409 | 0.248 | 0.007 | 2.7783E-121 | CAF-C | XCL2 |
| 2.3527E-125 | 0.986215098 | 0.786 | 0.26 | 5.8964E-121 | CAF-C | ARHGEF1 |
| 5.089E-125 | 0.664434106 | 0.544 | 0.096 | 1.2754E-120 | CAF-C | CENPW |
| 9.1706E-125 | 0.6897894 | 0.415 | 0.05 | 2.2983E-120 | CAF-C | NDC80 |
| 1.5199E-124 | 0.756157656 | 0.476 | 0.069 | 3.8092E-120 | CAF-C | CDCA7 |
| 2.9734E-124 | 0.639957097 | 0.505 | 0.081 | 7.452E-120 | CAF-C | CASP8 |
| 3.4862E-124 | 1.292981452 | 0.779 | 0.293 | 8.737E-120 | CAF-C | PPP1R18 |
| 5.0094E-124 | 1.04653486 | 0.701 | 0.164 | 1.2555E-119 | CAF-C | HLA-DRB1 |

| | | | | | | |
|---|---|---|---|---|---|---|
| 2.565E-123 | 0.729943252 | 0.43 | 0.056 | 6.4284E-119 | CAF-C | RACGAP1 |
| 6.7915E-123 | 0.885667091 | 0.313 | 0.022 | 1.7021E-118 | CAF-C | CD27 |
| 1.2987E-122 | 0.701523766 | 0.51 | 0.088 | 3.2548E-118 | CAF-C | EBP |
| 2.7368E-122 | 0.67966708 | 0.34 | 0.028 | 6.8591E-118 | CAF-C | HMMR |
| 5.4978E-122 | 0.728371568 | 0.49 | 0.08 | 1.3779E-117 | CAF-C | LIG1 |
| 9.2702E-122 | 0.629024817 | 0.485 | 0.078 | 2.3233E-117 | CAF-C | IL27RA |
| 1.325E-121 | 0.732935191 | 0.362 | 0.035 | 3.3206E-117 | CAF-C | LAG3 |
| 1.9302E-121 | 1.450669588 | 0.944 | 0.59 | 4.8375E-117 | CAF-C | ARPC1B |
| 4.1873E-121 | 1.894160796 | 0.964 | 0.713 | 1.0494E-116 | CAF-C | HMGN2 |
| 1.4266E-120 | 0.838528803 | 0.607 | 0.147 | 3.5754E-116 | CAF-C | RAB27A |
| 2.1333E-120 | 0.616720263 | 0.456 | 0.067 | 5.3464E-116 | CAF-C | DBF4 |
| 2.8224E-120 | 0.613211955 | 0.211 | 0.003 | 7.0736E-116 | CAF-C | CD40LG |
| 4.0679E-120 | 0.85709933 | 0.447 | 0.063 | 1.0195E-115 | CAF-C | PIM2 |
| 4.2847E-120 | 1.234747319 | 0.794 | 0.294 | 1.0738E-115 | CAF-C | CDK2AP2 |
| 6.3824E-120 | 0.951527685 | 0.216 | 0.004 | 1.5996E-115 | CAF-C | GZMH |
| 6.7998E-120 | 1.267226811 | 0.988 | 0.844 | 1.7042E-115 | CAF-C | RPSA |
| 8.0259E-120 | 1.152778592 | 0.752 | 0.248 | 2.0114E-115 | CAF-C | IDH2 |
| 1.2016E-119 | 1.671684245 | 0.942 | 0.634 | 3.0115E-115 | CAF-C | H2AFZ |
| 1.63E-119 | 0.685703176 | 0.578 | 0.104 | 4.085E-115 | CAF-C | HLA-DRB5 |
| 6.2293E-119 | 1.071321357 | 0.745 | 0.256 | 1.5612E-114 | CAF-C | RPA3 |
| 9.7493E-119 | 1.108481492 | 0.728 | 0.231 | 2.4434E-114 | CAF-C | ANP32E |
| 2.8214E-118 | 0.650353001 | 0.347 | 0.032 | 7.0711E-114 | CAF-C | CDCA3 |
| 4.3826E-118 | 0.711076499 | 0.592 | 0.132 | 1.0984E-113 | CAF-C | TSPAN14 |
| 2.5438E-117 | 1.494095011 | 0.867 | 0.35 | 6.3754E-113 | CAF-C | DDIT4 |
| 3.9497E-117 | 1.882313997 | 0.709 | 0.197 | 9.8986E-113 | CAF-C | CXCR4 |
| 4.1616E-116 | 1.510625655 | 0.277 | 0.017 | 1.043E-111 | CAF-C | CD8A |
| 1.2797E-115 | 0.641994831 | 0.517 | 0.094 | 3.2073E-111 | CAF-C | CNTRL |
| 1.8694E-115 | 0.904551468 | 0.687 | 0.205 | 4.6851E-111 | CAF-C | USP1 |
| 1.0438E-114 | 1.205778123 | 0.422 | 0.057 | 2.616E-110 | CAF-C | HIST1H1B |
| 1.4875E-114 | 0.756937431 | 0.318 | 0.026 | 3.728E-110 | CAF-C | PLK1 |
| 3.1762E-114 | 1.159725583 | 0.699 | 0.225 | 7.9603E-110 | CAF-C | TMEM106C |
| 7.6815E-114 | 0.624167309 | 0.478 | 0.079 | 1.9251E-109 | CAF-C | ARHGEF3 |
| 3.5109E-113 | 1.260107748 | 0.913 | 0.51 | 8.7991E-109 | CAF-C | PSMB9 |
| 7.8486E-113 | 1.194084666 | 0.927 | 0.544 | 1.967E-108 | CAF-C | TPM3 |
| 2.3772E-112 | 0.617088896 | 0.371 | 0.042 | 5.9578E-108 | CAF-C | KIF23 |
| 3.7988E-112 | 0.607311265 | 0.216 | 0.006 | 9.5205E-108 | CAF-C | CRTAM |
| 1.9838E-111 | 0.681534306 | 0.483 | 0.085 | 4.9717E-107 | CAF-C | CKAP2 |
| 1.4609E-110 | 0.650262008 | 0.432 | 0.063 | 3.6614E-106 | CAF-C | CRYBG1 |

| | | | | | | |
|---|---|---|---|---|---|---|
| 1.7529E-110 | 1.353804375 | 0.682 | 0.198 | 4.3931E-106 | CAF-C | CKS2 |
| 7.3823E-110 | 1.294619205 | 0.405 | 0.054 | 1.8501E-105 | CAF-C | HIST1H2AI |
| 3.2618E-109 | 0.972293431 | 0.684 | 0.219 | 8.1748E-105 | CAF-C | NUDT1 |
| 3.6174E-109 | 1.017437394 | 0.995 | 0.953 | 9.066E-105 | CAF-C | RPS19 |
| 8.5989E-109 | 1.290453701 | 0.879 | 0.479 | 2.1551E-104 | CAF-C | CALM3 |
| 2.9534E-108 | 1.26298275 | 0.968 | 0.483 | 7.4018E-104 | CAF-C | CRIP1 |
| 5.175E-108 | 1.175296757 | 0.774 | 0.305 | 1.297E-103 | CAF-C | ITGAE |
| 5.7999E-108 | 1.4692832 | 0.204 | 0.005 | 1.4536E-103 | CAF-C | XCL1 |
| 8.1232E-107 | 1.173676108 | 0.896 | 0.498 | 2.0358E-102 | CAF-C | ANP32B |
| 1.0536E-106 | 1.106104084 | 0.223 | 0.009 | 2.6404E-102 | CAF-C | IL2RA |
| 3.2392E-106 | 1.003796138 | 0.624 | 0.176 | 8.1181E-102 | CAF-C | MCM3 |
| 7.0858E-106 | 0.647596107 | 0.515 | 0.106 | 1.7758E-101 | CAF-C | CD58 |
| 8.5364E-106 | 1.001084364 | 0.235 | 0.011 | 2.1394E-101 | CAF-C | CD8B |
| 9.3713E-106 | 0.834837854 | 0.643 | 0.191 | 2.3486E-101 | CAF-C | CCDC167 |
| 5.5112E-105 | 1.156598835 | 0.927 | 0.558 | 1.3812E-100 | CAF-C | HMGN1 |
| 6.5044E-105 | 1.101114368 | 0.638 | 0.193 | 1.6301E-100 | CAF-C | CKS1B |
| 9.9501E-105 | 0.732314348 | 0.286 | 0.023 | 2.4937E-100 | CAF-C | LAT2 |
| 1.9113E-103 | 0.881841458 | 0.359 | 0.043 | 4.7901E-99 | CAF-C | CDC20 |
| 7.2144E-103 | 0.83537335 | 0.252 | 0.016 | 1.80808E-98 | CAF-C | KLRC2 |
| 7.7742E-103 | 0.643362584 | 0.417 | 0.065 | 1.94837E-98 | CAF-C | MGAT4A |
| 1.679E-102 | 0.809439771 | 0.609 | 0.157 | 4.20782E-98 | CAF-C | MIS18BP1 |
| 3.5653E-102 | 1.351609004 | 0.898 | 0.529 | 8.93539E-98 | CAF-C | H2AFV |
| 1.1361E-101 | 0.614745928 | 0.5 | 0.105 | 2.84721E-97 | CAF-C | RGS19 |
| 1.8263E-101 | 0.625448101 | 0.468 | 0.092 | 4.57702E-97 | CAF-C | CDCA4 |
| 2.3843E-101 | 1.487560925 | 0.968 | 0.682 | 5.97548E-97 | CAF-C | BTG1 |
| 4.0101E-101 | 0.874553116 | 0.398 | 0.062 | 1.005E-96 | CAF-C | AQP3 |
| 7.4564E-100 | 1.002437893 | 0.733 | 0.282 | 1.86872E-95 | CAF-C | DCXR |
| 1.9895E-99 | 1.133523027 | 0.944 | 0.667 | 4.98608E-95 | CAF-C | RPS29 |
| 2.6502E-99 | 1.007320471 | 0.748 | 0.297 | 6.64195E-95 | CAF-C | DNMT1 |
| 3.1071E-99 | 0.79525426 | 0.301 | 0.028 | 7.78702E-95 | CAF-C | HIST1H3B |
| 3.5938E-99 | 0.850059146 | 0.998 | 0.987 | 9.00667E-95 | CAF-C | RPLP1 |
| 3.6557E-99 | 0.943876402 | 0.959 | 0.54 | 9.16203E-95 | CAF-C | CD74 |
| 3.9922E-99 | 0.820570877 | 0.587 | 0.159 | 1.00053E-94 | CAF-C | RRM1 |
| 7.6586E-99 | 0.82746522 | 0.985 | 0.948 | 1.91941E-94 | CAF-C | RPS3 |
| 3.33388E-98 | 0.759897679 | 0.614 | 0.187 | 8.35538E-94 | CAF-C | DTYMK |
| 7.59065E-98 | 0.818763675 | 0.743 | 0.275 | 1.90237E-93 | CAF-C | FNBP1 |
| 9.19667E-98 | 0.622628074 | 0.481 | 0.096 | 2.30487E-93 | CAF-C | NCAPD2 |
| 9.50984E-98 | 0.7389725 | 0.592 | 0.154 | 2.38336E-93 | CAF-C | PIK3IP1 |

| | | | | | | |
|---|---|---|---|---|---|---|
| 1.66638E-97 | 2.690142758 | 0.82 | 0.407 | 4.17628E-93 | CAF-C | HIST1H4C |
| 6.01983E-97 | 0.715270767 | 0.386 | 0.057 | 1.50869E-92 | CAF-C | HPGD |
| 6.49714E-97 | 0.69098216 | 0.585 | 0.155 | 1.62831E-92 | CAF-C | ARL6IP6 |
| 2.50878E-96 | 1.071755568 | 0.84 | 0.414 | 6.28749E-92 | CAF-C | PAXX |
| 4.31217E-96 | 0.914785207 | 0.201 | 0.008 | 1.08072E-91 | CAF-C | FOXP3 |
| 7.10718E-96 | 1.153213534 | 0.934 | 0.682 | 1.7812E-91 | CAF-C | RAN |
| 1.10574E-95 | 0.683900356 | 0.49 | 0.102 | 2.7712E-91 | CAF-C | SEMA4D |
| 1.76955E-95 | 0.985324795 | 0.891 | 0.524 | 4.43486E-91 | CAF-C | PSMB8 |
| 2.31858E-95 | 1.048787064 | 0.633 | 0.202 | 5.81083E-91 | CAF-C | PCNA |
| 4.20893E-95 | 0.74007941 | 0.328 | 0.038 | 1.05484E-90 | CAF-C | HIST1H2AJ |
| 7.89757E-95 | 0.89272239 | 0.767 | 0.328 | 1.97929E-90 | CAF-C | RHOG |
| 7.91656E-95 | 1.107667266 | 0.883 | 0.524 | 1.98405E-90 | CAF-C | PPP1CA |
| 1.69069E-94 | 1.219915361 | 1 | 0.99 | 4.23721E-90 | CAF-C | ACTB |
| 2.87972E-94 | 0.914097924 | 0.643 | 0.188 | 7.21715E-90 | CAF-C | ITGA4 |
| 4.20848E-94 | 1.287174389 | 0.748 | 0.299 | 1.05473E-89 | CAF-C | H2AFX |
| 6.60934E-93 | 0.608024246 | 0.536 | 0.122 | 1.65643E-88 | CAF-C | FAM129A |
| 7.28167E-92 | 0.676459166 | 0.4 | 0.063 | 1.82493E-87 | CAF-C | HLA-DQA1 |
| 1.17442E-91 | 0.976342716 | 0.995 | 0.911 | 2.94333E-87 | CAF-C | CFL1 |
| 1.23945E-91 | 0.676257234 | 0.82 | 0.312 | 3.10631E-87 | CAF-C | EZR |
| 1.88688E-91 | 0.631705041 | 0.422 | 0.078 | 4.72889E-87 | CAF-C | SERPINB9 |
| 2.21544E-91 | 0.992412827 | 0.879 | 0.538 | 5.55232E-87 | CAF-C | SNRPB |
| 1.55113E-90 | 1.014753556 | 0.983 | 0.86 | 3.88745E-86 | CAF-C | RPS21 |
| 1.08237E-89 | 0.600130275 | 0.459 | 0.097 | 2.71263E-85 | CAF-C | CMTM7 |
| 1.48045E-89 | 0.66319969 | 0.551 | 0.142 | 3.7103E-85 | CAF-C | DIAPH1 |
| 2.44328E-89 | 0.718198883 | 0.488 | 0.116 | 6.12335E-85 | CAF-C | MCM6 |
| 5.54667E-89 | 0.893227127 | 0.721 | 0.262 | 1.39011E-84 | CAF-C | HLA-DPB1 |
| 1.29441E-88 | 0.845163426 | 0.726 | 0.294 | 3.24406E-84 | CAF-C | DDX39A |
| 1.72229E-88 | 0.791416746 | 0.995 | 0.94 | 4.31639E-84 | CAF-C | RPS15A |
| 4.4507E-88 | 0.963348541 | 0.915 | 0.581 | 1.11543E-83 | CAF-C | HNRNPF |
| 7.82866E-88 | 1.166846468 | 0.954 | 0.712 | 1.96202E-83 | CAF-C | ARPC2 |
| 2.43492E-87 | 0.62687297 | 0.636 | 0.211 | 6.1024E-83 | CAF-C | SS18L2 |
| 3.37814E-87 | 0.845888151 | 0.981 | 0.873 | 8.4663E-83 | CAF-C | RPL23A |
| 3.7707E-87 | 0.679990261 | 0.255 | 0.022 | 9.45013E-83 | CAF-C | HIST1H3C |
| 4.62467E-87 | 0.877655879 | 0.529 | 0.141 | 1.15904E-82 | CAF-C | CDC25B |
| 5.3485E-87 | 3.298253986 | 0.226 | 0.016 | 1.34044E-82 | CAF-C | GNLY |
| 7.67788E-87 | 0.816697556 | 0.665 | 0.223 | 1.92423E-82 | CAF-C | LBR |
| 1.11692E-86 | 1.037378388 | 0.886 | 0.537 | 2.79923E-82 | CAF-C | DEK |
| 2.53002E-86 | 1.049943228 | 0.745 | 0.288 | 6.34074E-82 | CAF-C | ID2 |

| | | | | | | |
|---|---|---|---|---|---|---|
| 3.523E-86 | 0.967567763 | 0.854 | 0.419 | 8.82935E-82 | CAF-C | RNF213 |
| 1.10877E-85 | 0.959832112 | 0.74 | 0.327 | 2.77879E-81 | CAF-C | SLBP |
| 4.21478E-85 | 0.614972264 | 0.575 | 0.171 | 1.05631E-80 | CAF-C | MZT1 |
| 4.69063E-85 | 1.022457522 | 0.985 | 0.706 | 1.17557E-80 | CAF-C | HLA-E |
| 1.22882E-84 | 0.938204594 | 0.655 | 0.23 | 3.07967E-80 | CAF-C | C12orf75 |
| 1.72193E-84 | 0.835652842 | 0.772 | 0.343 | 4.31551E-80 | CAF-C | TAP1 |
| 2.87892E-84 | 0.761190609 | 0.735 | 0.314 | 7.21515E-80 | CAF-C | NUDT21 |
| 8.08964E-84 | 1.040114906 | 0.956 | 0.628 | 2.02743E-79 | CAF-C | SARAF |
| 2.59128E-83 | 0.945424749 | 0.883 | 0.483 | 6.49426E-79 | CAF-C | FXYD5 |
| 3.26931E-83 | 0.884957443 | 0.667 | 0.231 | 8.19354E-79 | CAF-C | SOCS1 |
| 4.31095E-83 | 0.608185263 | 0.206 | 0.013 | 1.08041E-78 | CAF-C | TNF |
| 8.55432E-83 | 0.636631314 | 0.553 | 0.153 | 2.14388E-78 | CAF-C | MBP |
| 8.56652E-83 | 0.622186092 | 0.544 | 0.158 | 2.14694E-78 | CAF-C | ZDHHC12 |
| 8.96373E-83 | 1.281210969 | 0.903 | 0.646 | 2.24649E-78 | CAF-C | RPS10 |
| 1.05661E-82 | 1.092337198 | 0.983 | 0.827 | 2.64807E-78 | CAF-C | HMGB1 |
| 4.42822E-82 | 0.810137121 | 0.604 | 0.2 | 1.1098E-77 | CAF-C | KIF22 |
| 1.2379E-81 | 0.972720426 | 0.638 | 0.226 | 3.10241E-77 | CAF-C | CREM |
| 9.0414E-81 | 0.846336876 | 0.534 | 0.15 | 2.26596E-76 | CAF-C | CAPG |
| 9.83555E-81 | 0.891601297 | 0.99 | 0.926 | 2.46499E-76 | CAF-C | RPS27 |
| 1.57501E-80 | 1.038913948 | 0.888 | 0.557 | 3.94729E-76 | CAF-C | ACTR3 |
| 3.18555E-80 | 0.855355482 | 0.772 | 0.383 | 7.98363E-76 | CAF-C | LSM4 |
| 4.64437E-80 | 0.685335377 | 0.558 | 0.166 | 1.16397E-75 | CAF-C | PPP2R5C |
| 4.66541E-80 | 1.548981852 | 0.871 | 0.45 | 1.16924E-75 | CAF-C | TNFAIP3 |
| 5.59607E-80 | 0.949288131 | 0.772 | 0.389 | 1.40249E-75 | CAF-C | CACYBP |
| 1.55355E-79 | 0.878394382 | 0.716 | 0.313 | 3.89352E-75 | CAF-C | CENPX |
| 5.5829E-79 | 0.846698072 | 0.879 | 0.528 | 1.39919E-74 | CAF-C | RALY |
| 5.86268E-79 | 0.849701489 | 0.966 | 0.776 | 1.4693E-74 | CAF-C | ATP5MG |
| 1.12817E-78 | 1.178794608 | 0.995 | 0.947 | 2.82741E-74 | CAF-C | GAPDH |
| 1.34017E-78 | 0.657500118 | 0.646 | 0.232 | 3.35873E-74 | CAF-C | TRABD |
| 1.56606E-78 | 0.777576235 | 0.801 | 0.378 | 3.92487E-74 | CAF-C | MZT2A |
| 4.95275E-78 | 1.0229387 | 0.922 | 0.652 | 1.24126E-73 | CAF-C | SUB1 |
| 5.57843E-78 | 0.753488044 | 0.291 | 0.04 | 1.39807E-73 | CAF-C | RBKS |
| 1.93812E-77 | 0.654563321 | 0.641 | 0.234 | 4.85732E-73 | CAF-C | HPRT1 |
| 2.77577E-77 | 1.644882757 | 0.925 | 0.713 | 6.95664E-73 | CAF-C | TUBB |
| 4.964E-77 | 0.713898343 | 0.726 | 0.314 | 1.24408E-72 | CAF-C | CMC2 |
| 5.43599E-77 | 0.64192846 | 0.675 | 0.228 | 1.36237E-72 | CAF-C | SYNE2 |
| 2.02505E-76 | 0.745739942 | 0.733 | 0.336 | 5.07517E-72 | CAF-C | ARPC5L |
| 2.31959E-76 | 0.714610924 | 1 | 0.981 | 5.81336E-72 | CAF-C | B2M |

| | | | | | | |
|---|---|---|---|---|---|---|
| 3.88557E-76 | 0.77611904 | 0.869 | 0.517 | 9.73801E-72 | CAF-C | UBE2D2 |
| 5.68959E-76 | 1.057988352 | 0.92 | 0.666 | 1.42593E-71 | CAF-C | SLC25A5 |
| 1.69094E-75 | 0.939167509 | 0.917 | 0.699 | 4.23783E-71 | CAF-C | COX8A |
| 2.15729E-75 | 0.749877513 | 0.789 | 0.377 | 5.4066E-71 | CAF-C | 9-Sep |
| 3.47237E-75 | 0.848085859 | 0.833 | 0.452 | 8.70247E-71 | CAF-C | PSMB10 |
| 3.77116E-75 | 1.297631491 | 0.825 | 0.531 | 9.45127E-71 | CAF-C | DUT |
| 8.5258E-75 | 3.155152078 | 0.318 | 0.052 | 2.13674E-70 | CAF-C | CCL4L2 |
| 1.41001E-74 | 0.745659626 | 0.731 | 0.315 | 3.53376E-70 | CAF-C | LEPROTL1 |
| 1.48693E-74 | 0.628860897 | 0.524 | 0.142 | 3.72653E-70 | CAF-C | BIRC3 |
| 4.06383E-74 | 0.671678708 | 0.357 | 0.065 | 1.01848E-69 | CAF-C | HIST1H2AG |
| 1.24544E-73 | 0.840090125 | 0.714 | 0.328 | 3.12133E-69 | CAF-C | RNF167 |
| 3.00486E-73 | 1.032447312 | 0.99 | 0.947 | 7.53078E-69 | CAF-C | ACTG1 |
| 6.00788E-73 | 0.846711462 | 0.82 | 0.451 | 1.50569E-68 | CAF-C | PA2G4 |
| 6.29939E-73 | 0.951533296 | 0.837 | 0.541 | 1.57875E-68 | CAF-C | SNRPG |
| 8.36436E-73 | 0.665351661 | 0.733 | 0.312 | 2.09628E-68 | CAF-C | EML4 |
| 1.60847E-72 | 0.731122901 | 0.536 | 0.148 | 4.03115E-68 | CAF-C | RGCC |
| 1.72238E-72 | 0.844540847 | 0.333 | 0.059 | 4.31662E-68 | CAF-C | CCNB1 |
| 2.26157E-72 | 0.811925674 | 0.934 | 0.698 | 5.66795E-68 | CAF-C | ARPC3 |
| 5.36285E-72 | 0.761752477 | 0.794 | 0.417 | 1.34404E-67 | CAF-C | PRR13 |
| 8.37326E-72 | 0.776327824 | 0.998 | 0.968 | 2.09851E-67 | CAF-C | PTMA |
| 9.72668E-72 | 0.648793029 | 0.653 | 0.26 | 2.4377E-67 | CAF-C | RNASEH2B |
| 1.55842E-71 | 0.885398759 | 0.922 | 0.615 | 3.90572E-67 | CAF-C | SRSF7 |
| 2.45739E-71 | 0.611959365 | 0.51 | 0.153 | 6.15872E-67 | CAF-C | APOBEC3C |
| 3.19951E-71 | 0.751127377 | 0.782 | 0.412 | 8.01861E-67 | CAF-C | SNRPF |
| 1.1137E-70 | 0.689733296 | 0.643 | 0.254 | 2.79116E-66 | CAF-C | SKA2 |
| 1.36603E-70 | 0.87033994 | 0.985 | 0.843 | 3.42354E-66 | CAF-C | HSPA8 |
| 2.58564E-70 | 0.633006587 | 0.694 | 0.299 | 6.48012E-66 | CAF-C | FAM96A |
| 4.16235E-70 | 1.075354951 | 0.534 | 0.177 | 1.04317E-65 | CAF-C | KPNA2 |
| 4.34265E-70 | 0.785011035 | 0.947 | 0.743 | 1.08835E-65 | CAF-C | HINT1 |
| 4.48772E-70 | 0.757791132 | 0.976 | 0.785 | 1.12471E-65 | CAF-C | OAZ1 |
| 1.05057E-69 | 0.9617117 | 0.883 | 0.589 | 2.63294E-65 | CAF-C | PCBP2 |
| 3.1481E-69 | 0.71156935 | 0.76 | 0.359 | 7.88977E-65 | CAF-C | NAP1L4 |
| 4.45605E-69 | 1.250091455 | 0.294 | 0.048 | 1.11678E-64 | CAF-C | TNFRSF4 |
| 4.52207E-69 | 0.839038604 | 0.925 | 0.61 | 1.13332E-64 | CAF-C | PSME2 |
| 4.72763E-69 | 0.71737605 | 0.648 | 0.263 | 1.18484E-64 | CAF-C | GYG1 |
| 4.77468E-69 | 0.684363271 | 0.784 | 0.415 | 1.19663E-64 | CAF-C | PPM1G |
| 5.08497E-69 | 1.463567141 | 0.539 | 0.171 | 1.2744E-64 | CAF-C | IL7R |
| 9.09722E-69 | 0.739749488 | 0.862 | 0.514 | 2.27995E-64 | CAF-C | TRAPPC1 |

| | | | | | | |
|---|---|---|---|---|---|---|
| 1.56033E-68 | 0.808760448 | 0.648 | 0.254 | 3.91049E-64 | CAF-C | PIM1 |
| 4.51202E-68 | 1.181070178 | 0.291 | 0.047 | 1.1308E-63 | CAF-C | FCER1G |
| 1.17995E-67 | 0.762712172 | 0.779 | 0.431 | 2.95719E-63 | CAF-C | SNRPD1 |
| 2.17882E-67 | 0.701476479 | 0.806 | 0.443 | 5.46057E-63 | CAF-C | RNPS1 |
| 4.0884E-67 | 0.760008474 | 0.947 | 0.731 | 1.02463E-62 | CAF-C | TMA7 |
| 4.66597E-67 | 0.839916208 | 0.867 | 0.584 | 1.16939E-62 | CAF-C | COX5A |
| 1.06805E-66 | 0.678707641 | 0.777 | 0.411 | 2.67676E-62 | CAF-C | UBE2N |
| 1.19791E-66 | 0.618019607 | 0.65 | 0.262 | 3.0022E-62 | CAF-C | BIN1 |
| 1.87462E-66 | 0.686977564 | 0.762 | 0.374 | 4.69817E-62 | CAF-C | ARF6 |
| 2.63982E-66 | 0.724370945 | 0.973 | 0.808 | 6.61593E-62 | CAF-C | HNRNPA1 |
| 7.17915E-66 | 0.769405987 | 0.684 | 0.304 | 1.79924E-61 | CAF-C | SMC1A |
| 7.64336E-66 | 0.872645714 | 0.813 | 0.481 | 1.91558E-61 | CAF-C | RANBP1 |
| 6.8364E-65 | 0.816188139 | 0.981 | 0.86 | 1.71334E-60 | CAF-C | H3F3A |
| 9.5152E-65 | 0.615944975 | 0.993 | 0.969 | 2.3847E-60 | CAF-C | RPL28 |
| 1.29178E-64 | 0.693664735 | 0.903 | 0.528 | 3.23747E-60 | CAF-C | MBNL1 |
| 2.67696E-64 | 0.614839214 | 0.617 | 0.244 | 6.709E-60 | CAF-C | SAE1 |
| 3.12021E-64 | 0.781233197 | 0.966 | 0.813 | 7.81987E-60 | CAF-C | ATP5F1E |
| 7.22747E-63 | 1.163551441 | 0.845 | 0.51 | 1.81135E-58 | CAF-C | RARRES3 |
| 9.00391E-63 | 2.857988484 | 0.425 | 0.118 | 2.25656E-58 | CAF-C | CCL4 |
| 1.90261E-62 | 0.89207923 | 0.786 | 0.419 | 4.76831E-58 | CAF-C | RAD21 |
| 4.64271E-62 | 0.663171283 | 0.672 | 0.295 | 1.16356E-57 | CAF-C | CCND3 |
| 5.94282E-62 | 0.612408916 | 0.718 | 0.319 | 1.48939E-57 | CAF-C | WNK1 |
| 6.53277E-62 | 0.611238232 | 0.614 | 0.244 | 1.63724E-57 | CAF-C | VOPP1 |
| 1.57198E-61 | 0.745583003 | 0.903 | 0.587 | 3.9397E-57 | CAF-C | SRSF2 |
| 1.90195E-60 | 0.668166936 | 0.796 | 0.41 | 4.76667E-56 | CAF-C | RGS10 |
| 2.13153E-60 | 0.663000916 | 0.728 | 0.377 | 5.34204E-56 | CAF-C | LSM5 |
| 2.34848E-60 | 0.609844601 | 0.689 | 0.31 | 5.88576E-56 | CAF-C | NOP58 |
| 2.50029E-60 | 0.731295333 | 0.956 | 0.803 | 6.26623E-56 | CAF-C | CHCHD2 |
| 7.85693E-60 | 0.770635871 | 0.91 | 0.608 | 1.9691E-55 | CAF-C | PGK1 |
| 1.04228E-59 | 0.61734048 | 0.444 | 0.128 | 2.61217E-55 | CAF-C | PDE4B |
| 1.53221E-59 | 0.679901271 | 0.614 | 0.24 | 3.84002E-55 | CAF-C | LDLRAD4 |
| 2.45428E-59 | 0.6109976 | 0.617 | 0.266 | 6.15092E-55 | CAF-C | PMVK |
| 3.69355E-59 | 0.695868105 | 0.51 | 0.164 | 9.25678E-55 | CAF-C | PMAIP1 |
| 3.76557E-59 | 0.703425846 | 0.709 | 0.306 | 9.43726E-55 | CAF-C | ISG20 |
| 6.45605E-59 | 0.773433353 | 0.733 | 0.354 | 1.61802E-54 | CAF-C | CLEC2B |
| 6.7458E-59 | 0.633851294 | 0.701 | 0.336 | 1.69063E-54 | CAF-C | SLC25A39 |
| 7.05182E-59 | 0.732318653 | 0.26 | 0.044 | 1.76733E-54 | CAF-C | KLRD1 |
| 9.55894E-59 | 0.994207062 | 0.903 | 0.662 | 2.39566E-54 | CAF-C | ENO1 |

| | | | | | | |
|---|---|---|---|---|---|---|
| 1.0945E-58 | 0.624155587 | 0.864 | 0.512 | 2.74303E-54 | CAF-C | SRSF10 |
| 1.2193E-58 | 0.750499659 | 0.201 | 0.023 | 3.05581E-54 | CAF-C | CCL3L1 |
| 1.47708E-58 | 0.605845931 | 0.769 | 0.406 | 3.70186E-54 | CAF-C | PAK2 |
| 9.10713E-58 | 0.608275866 | 0.871 | 0.542 | 2.28243E-53 | CAF-C | CIB1 |
| 2.28999E-57 | 0.60343673 | 0.847 | 0.515 | 5.73916E-53 | CAF-C | CBX3 |
| 5.28545E-57 | 0.66199534 | 0.743 | 0.405 | 1.32464E-52 | CAF-C | LSM2 |
| 4.15653E-56 | 0.607197193 | 0.845 | 0.463 | 1.04171E-51 | CAF-C | GYPC |
| 4.98294E-56 | 0.767953436 | 0.983 | 0.92 | 1.24883E-51 | CAF-C | RPL41 |
| 1.2558E-55 | 0.62825738 | 0.985 | 0.957 | 3.14728E-51 | CAF-C | RPS4X |
| 1.41698E-55 | 1.074512587 | 0.221 | 0.031 | 3.55123E-51 | CAF-C | CCL3 |
| 3.67722E-55 | 0.705315855 | 0.82 | 0.482 | 9.21584E-51 | CAF-C | LRRFIP1 |
| 6.00513E-55 | 0.634558087 | 0.886 | 0.587 | 1.50501E-50 | CAF-C | DBI |
| 9.07434E-55 | 0.621000533 | 0.735 | 0.361 | 2.27421E-50 | CAF-C | SMCHD1 |
| 1.0187E-54 | 0.604568721 | 0.893 | 0.545 | 2.55308E-50 | CAF-C | MSN |
| 1.15034E-54 | 0.616510272 | 0.993 | 0.851 | 2.88299E-50 | CAF-C | HNRNPA2B1 |
| 1.83815E-54 | 0.611167097 | 0.471 | 0.15 | 4.60676E-50 | CAF-C | PTGER4 |
| 2.97645E-54 | 0.696597667 | 0.854 | 0.564 | 7.45958E-50 | CAF-C | PCBP1 |
| 3.75978E-54 | 0.725172472 | 0.993 | 0.909 | 9.42275E-50 | CAF-C | HLA-C |
| 5.98576E-54 | 0.724721016 | 0.617 | 0.26 | 1.50015E-49 | CAF-C | REL |
| 3.7216E-53 | 1.445436562 | 0.879 | 0.623 | 9.32707E-49 | CAF-C | S100A4 |
| 1.39373E-52 | 0.624264246 | 0.621 | 0.288 | 3.49297E-48 | CAF-C | MAD2L2 |
| 2.14123E-52 | 0.607466493 | 0.762 | 0.427 | 5.36635E-48 | CAF-C | NAA38 |
| 2.6918E-52 | 0.76413346 | 0.917 | 0.639 | 6.74618E-48 | CAF-C | PGAM1 |
| 5.09745E-52 | 0.834928972 | 0.881 | 0.584 | 1.27752E-47 | CAF-C | CNN2 |
| 1.45083E-51 | 1.289178068 | 0.959 | 0.775 | 3.63606E-47 | CAF-C | TUBA1B |
| 2.39385E-51 | 0.681585081 | 0.927 | 0.691 | 5.99947E-47 | CAF-C | PSME1 |
| 3.95219E-51 | 0.671285899 | 0.592 | 0.254 | 9.90498E-47 | CAF-C | RORA |
| 4.2503E-51 | 0.637329439 | 0.852 | 0.547 | 1.06521E-46 | CAF-C | GSTK1 |
| 4.42766E-51 | 0.625592989 | 0.59 | 0.26 | 1.10966E-46 | CAF-C | SH3KBP1 |
| 6.05839E-51 | 0.635907689 | 0.944 | 0.776 | 1.51835E-46 | CAF-C | CLIC1 |
| 2.2263E-49 | 0.737166105 | 0.801 | 0.522 | 5.57954E-45 | CAF-C | SIVA1 |
| 2.9603E-49 | 0.709552839 | 0.978 | 0.823 | 7.41909E-45 | CAF-C | RPL17 |
| 4.15141E-49 | 0.682577516 | 0.862 | 0.562 | 1.04043E-44 | CAF-C | WDR1 |
| 5.61156E-49 | 0.637019175 | 0.886 | 0.587 | 1.40637E-44 | CAF-C | CAP1 |
| 9.83711E-49 | 1.244354348 | 0.296 | 0.07 | 2.46538E-44 | CAF-C | TYROBP |
| 1.00271E-47 | 0.680274647 | 0.733 | 0.374 | 2.513E-43 | CAF-C | ETS1 |
| 1.78128E-47 | 0.793478497 | 0.956 | 0.781 | 4.46424E-43 | CAF-C | TPI1 |
| 2.76563E-47 | 0.605442158 | 0.847 | 0.513 | 6.93122E-43 | CAF-C | ARL6IP5 |

| | | | | | | |
|---|---|---|---|---|---|---|
| 5.69976E-47 | 0.645840619 | 0.91 | 0.665 | 1.42847E-42 | CAF-C | LDHB |
| 3.49283E-46 | 0.660253342 | 0.631 | 0.293 | 8.75372E-42 | CAF-C | ADGRE5 |
| 5.25088E-44 | 0.604100874 | 0.653 | 0.32 | 1.31598E-39 | CAF-C | CCNH |
| 1.14928E-43 | 0.660414476 | 0.743 | 0.437 | 2.88034E-39 | CAF-C | DNPH1 |
| 3.98527E-43 | 0.645683837 | 0.553 | 0.244 | 9.98789E-39 | CAF-C | SMAP2 |
| 7.75741E-43 | 0.652851241 | 0.816 | 0.561 | 1.94416E-38 | CAF-C | SELENOH |
| 5.39552E-42 | 0.708785813 | 0.551 | 0.252 | 1.35223E-37 | CAF-C | RNF19A |
| 6.99885E-41 | 0.711416441 | 0.939 | 0.744 | 1.75405E-36 | CAF-C | LDHA |
| 1.34793E-40 | 0.699882955 | 0.619 | 0.307 | 3.37819E-36 | CAF-C | PRDM1 |
| 7.64534E-38 | 0.795175377 | 0.551 | 0.265 | 1.91607E-33 | CAF-C | HIST1H1C |
| 9.8135E-36 | 0.601441719 | 0.633 | 0.351 | 2.45946E-31 | CAF-C | RBPJ |
| 1.11656E-35 | 0.804421699 | 0.917 | 0.664 | 2.79833E-31 | CAF-C | KLF6 |
| 1.2734E-33 | 0.863936341 | 0.927 | 0.703 | 3.1914E-29 | CAF-C | ZFP36L2 |
| 4.47987E-32 | 0.609389881 | 0.898 | 0.684 | 1.12274E-27 | CAF-C | HSPE1 |
| 1.12735E-31 | 1.076937474 | 0.752 | 0.495 | 2.82538E-27 | CAF-C | ARL6IP1 |
| 5.7205E-31 | 0.629220675 | 0.502 | 0.257 | 1.43367E-26 | CAF-C | STARD3NL |
| 7.86921E-30 | 0.626060738 | 0.973 | 0.894 | 1.97218E-25 | CAF-C | MT-ATP8 |
| 5.82888E-22 | 0.886483601 | 0.403 | 0.206 | 1.46083E-17 | CAF-C | HOPX |
| 4.94153E-20 | 0.81332063 | 0.638 | 0.438 | 1.23845E-15 | CAF-C | HIST1H1E |
| 5.19505E-14 | 0.650297904 | 0.313 | 0.161 | 1.30198E-09 | CAF-C | AREG |
| 0 | 2.257815714 | 0.862 | 0.064 | 0 | CAF-B | TINAGL1 |
| 0 | 2.088281297 | 0.711 | 0.029 | 0 | CAF-B | GJA4 |
| 0 | 1.693399882 | 0.793 | 0.052 | 0 | CAF-B | EFHD1 |
| 1.8347E-295 | 1.26231975 | 0.531 | 0.01 | 4.5982E-291 | CAF-B | OR51E1 |
| 8.3215E-289 | 1.722057969 | 0.652 | 0.031 | 2.0855E-284 | CAF-B | ANGPT2 |
| 1.9925E-286 | 1.556054863 | 0.751 | 0.048 | 4.9937E-282 | CAF-B | ESAM |
| 6.5529E-272 | 1.408375368 | 0.633 | 0.033 | 1.6423E-267 | CAF-B | GJC1 |
| 8.4529E-261 | 2.674365688 | 0.938 | 0.159 | 2.1185E-256 | CAF-B | NOTCH3 |
| 5.1562E-257 | 1.162515041 | 0.515 | 0.016 | 1.2922E-252 | CAF-B | ENPEP |
| 7.916E-251 | 1.288627495 | 0.659 | 0.046 | 1.9839E-246 | CAF-B | CDH6 |
| 3.731E-249 | 0.953343633 | 0.492 | 0.014 | 9.3506E-245 | CAF-B | FHL5 |
| 5.3851E-248 | 1.770701384 | 0.63 | 0.037 | 1.3496E-243 | CAF-B | COX4I2 |
| 1.1023E-233 | 2.666352708 | 0.931 | 0.177 | 2.7625E-229 | CAF-B | MCAM |
| 1.108E-233 | 1.830396636 | 0.836 | 0.112 | 2.7768E-229 | CAF-B | HEYL |
| 5.599E-233 | 1.035997963 | 0.495 | 0.018 | 1.4032E-228 | CAF-B | ITGA7 |
| 2.097E-230 | 1.019346729 | 0.502 | 0.02 | 5.2555E-226 | CAF-B | ADAP2 |
| 5.6419E-227 | 1.173647895 | 0.639 | 0.05 | 1.414E-222 | CAF-B | EBF1 |
| 9.8824E-227 | 1.638173692 | 0.659 | 0.057 | 2.4767E-222 | CAF-B | CSPG4 |

| | | | | | | |
|---|---|---|---|---|---|---|
| 9.1691E-224 | 2.741053449 | 0.856 | 0.133 | 2.298E-219 | CAF-B | NDUFA4L2 |
| 1.0026E-216 | 1.439927796 | 0.534 | 0.028 | 2.5126E-212 | CAF-B | HIGD1B |
| 6.7727E-215 | 1.288081131 | 0.584 | 0.039 | 1.6974E-210 | CAF-B | ADGRF5 |
| 1.0807E-207 | 2.214131801 | 0.731 | 0.094 | 2.7084E-203 | CAF-B | CPE |
| 2.6518E-207 | 0.970406928 | 0.413 | 0.012 | 6.646E-203 | CAF-B | GPR4 |
| 5.8178E-201 | 1.216542629 | 0.42 | 0.014 | 1.4581E-196 | CAF-B | ACAN |
| 3.9099E-200 | 1.4105688 | 0.557 | 0.043 | 9.7991E-196 | CAF-B | MAP3K7CL |
| 7.9685E-197 | 0.877583668 | 0.41 | 0.013 | 1.9971E-192 | CAF-B | SEMA5B |
| 2.159E-194 | 1.147535464 | 0.534 | 0.039 | 5.411E-190 | CAF-B | PLCE1 |
| 1.3119E-191 | 1.230105873 | 0.603 | 0.058 | 3.2879E-187 | CAF-B | FOXS1 |
| 3.4851E-191 | 0.765008775 | 0.41 | 0.015 | 8.7343E-187 | CAF-B | LGI4 |
| 1.0455E-190 | 1.18000636 | 0.452 | 0.023 | 2.6202E-186 | CAF-B | EGFLAM |
| 2.6745E-186 | 1.646536772 | 0.692 | 0.092 | 6.7029E-182 | CAF-B | PTP4A3 |
| 3.9257E-186 | 1.50585415 | 0.748 | 0.11 | 9.8387E-182 | CAF-B | MEF2C |
| 3.8934E-184 | 2.396478868 | 0.879 | 0.196 | 9.7576E-180 | CAF-B | CSRP2 |
| 1.1892E-181 | 0.86295257 | 0.472 | 0.03 | 2.9804E-177 | CAF-B | HEY2 |
| 3.4767E-180 | 1.043631895 | 0.626 | 0.07 | 8.7132E-176 | CAF-B | PDGFA |
| 5.7454E-174 | 1.352275589 | 0.639 | 0.078 | 1.4399E-169 | CAF-B | CCDC3 |
| 1.4088E-173 | 2.906116029 | 0.895 | 0.212 | 3.5308E-169 | CAF-B | RGS5 |
| 1.4164E-171 | 1.336503065 | 0.443 | 0.028 | 3.5499E-167 | CAF-B | SERPINI1 |
| 1.6272E-168 | 1.682219043 | 0.711 | 0.114 | 4.078E-164 | CAF-B | SLIT3 |
| 2.3607E-164 | 0.649064525 | 0.43 | 0.025 | 5.9163E-160 | CAF-B | PCDH1 |
| 2.0358E-163 | 0.796776666 | 0.443 | 0.03 | 5.102E-159 | CAF-B | LINC02202 |
| 7.8562E-162 | 0.64880206 | 0.295 | 0.005 | 1.9689E-157 | CAF-B | AVPR1A |
| 6.5653E-161 | 0.864947867 | 0.446 | 0.032 | 1.6454E-156 | CAF-B | LZTS1 |
| 3.5679E-157 | 1.465179229 | 0.744 | 0.149 | 8.942E-153 | CAF-B | 4-Sep |
| 1.3881E-155 | 1.650536875 | 0.61 | 0.086 | 3.4789E-151 | CAF-B | BCAM |
| 8.2008E-155 | 1.332440775 | 0.679 | 0.111 | 2.0553E-150 | CAF-B | KCNE4 |
| 3.7019E-154 | 1.167181999 | 0.531 | 0.058 | 9.2776E-150 | CAF-B | FAM162B |
| 6.2206E-151 | 1.666671632 | 0.826 | 0.208 | 1.559E-146 | CAF-B | JAG1 |
| 1.5652E-148 | 0.753310774 | 0.436 | 0.034 | 3.9228E-144 | CAF-B | ANO1 |
| 1.2338E-145 | 1.060764214 | 0.561 | 0.076 | 3.0922E-141 | CAF-B | PTGIR |
| 3.805E-145 | 0.623482953 | 0.348 | 0.017 | 9.536E-141 | CAF-B | IL17B |
| 5.6046E-143 | 0.903836184 | 0.554 | 0.069 | 1.4046E-138 | CAF-B | ARHGAP29 |
| 1.0164E-142 | 1.633309713 | 0.823 | 0.205 | 2.5473E-138 | CAF-B | MAP1B |
| 3.1644E-142 | 1.179355793 | 0.639 | 0.102 | 7.9306E-138 | CAF-B | NFASC |
| 1.3755E-141 | 1.406740494 | 0.662 | 0.127 | 3.4474E-137 | CAF-B | C1QTNF1 |
| 2.4567E-140 | 1.185655591 | 0.6 | 0.093 | 6.1569E-136 | CAF-B | INPP4B |

| | | | | | | |
|---|---|---|---|---|---|---|
| 4.8507E-139 | 1.178852261 | 0.61 | 0.096 | 1.2157E-134 | CAF-B | CLMN |
| 1.2591E-136 | 1.361768257 | 0.633 | 0.107 | 3.1556E-132 | CAF-B | SMOC2 |
| 2.7576E-135 | 2.8402661 | 0.826 | 0.243 | 6.9111E-131 | CAF-B | ADIRF |
| 1.5545E-133 | 0.923607901 | 0.58 | 0.09 | 3.8959E-129 | CAF-B | RASGRP2 |
| 2.3927E-131 | 0.725237289 | 0.348 | 0.022 | 5.9966E-127 | CAF-B | NRIP2 |
| 3.469E-128 | 1.007258227 | 0.574 | 0.089 | 8.694E-124 | CAF-B | FAM13C |
| 3.5316E-127 | 0.729861521 | 0.387 | 0.032 | 8.8509E-123 | CAF-B | NRARP |
| 2.7033E-124 | 1.02378971 | 0.459 | 0.054 | 6.7751E-120 | CAF-B | KCNJ8 |
| 8.8094E-123 | 0.816251589 | 0.498 | 0.069 | 2.2078E-118 | CAF-B | NEURL1B |
| 4.9795E-122 | 1.260893866 | 0.708 | 0.161 | 1.248E-117 | CAF-B | RCAN2 |
| 5.6907E-122 | 0.911942696 | 0.357 | 0.028 | 1.4262E-117 | CAF-B | SLC7A2 |
| 1.2179E-119 | 1.506650156 | 0.669 | 0.145 | 3.0524E-115 | CAF-B | OLFML2B |
| 3.6527E-118 | 2.050376507 | 0.967 | 0.517 | 9.1544E-114 | CAF-B | SOD3 |
| 4.1211E-118 | 1.359149365 | 0.833 | 0.3 | 1.0328E-113 | CAF-B | PTK2 |
| 1.2141E-117 | 1.340939196 | 0.757 | 0.207 | 3.0427E-113 | CAF-B | TBX2 |
| 1.0675E-115 | 1.918350704 | 0.908 | 0.427 | 2.6755E-111 | CAF-B | LHFPL6 |
| 4.0169E-115 | 1.250261649 | 0.721 | 0.198 | 1.0067E-110 | CAF-B | ARHGEF17 |
| 5.6416E-115 | 1.064609183 | 0.58 | 0.102 | 1.4139E-110 | CAF-B | TPPP3 |
| 6.363E-114 | 1.673827836 | 0.892 | 0.382 | 1.5947E-109 | CAF-B | NR2F2 |
| 1.0268E-113 | 1.804077652 | 0.898 | 0.421 | 2.5732E-109 | CAF-B | PDGFRB |
| 5.1936E-113 | 1.415630846 | 0.889 | 0.379 | 1.3016E-108 | CAF-B | EPS8 |
| 7.8954E-113 | 0.698946661 | 0.4 | 0.041 | 1.9787E-108 | CAF-B | GUCY1A2 |
| 9.9001E-112 | 1.812463205 | 0.977 | 0.561 | 2.4812E-107 | CAF-B | COL18A1 |
| 2.2575E-111 | 1.140940126 | 0.544 | 0.095 | 5.6578E-107 | CAF-B | CCDC102B |
| 2.9207E-111 | 0.742435386 | 0.374 | 0.036 | 7.3198E-107 | CAF-B | REM1 |
| 7.9082E-110 | 0.95131693 | 0.39 | 0.041 | 1.9819E-105 | CAF-B | ABCC9 |
| 1.3346E-108 | 1.026517407 | 0.502 | 0.081 | 3.3448E-104 | CAF-B | MOCS1 |
| 1.8311E-108 | 0.885648147 | 0.351 | 0.032 | 4.5891E-104 | CAF-B | GPRC5C |
| 2.2785E-108 | 1.715392396 | 0.659 | 0.14 | 5.7104E-104 | CAF-B | MYH11 |
| 4.0172E-108 | 1.45282821 | 0.554 | 0.106 | 1.0068E-103 | CAF-B | ADAMTS4 |
| 4.1723E-108 | 1.447138254 | 0.997 | 0.821 | 1.0457E-103 | CAF-B | IGFBP7 |
| 1.4312E-107 | 1.050464168 | 0.521 | 0.092 | 3.5868E-103 | CAF-B | S1PR3 |
| 6.3287E-106 | 1.347601072 | 0.656 | 0.152 | 1.5861E-101 | CAF-B | RGS16 |
| 2.2016E-105 | 1.414014981 | 0.849 | 0.324 | 5.5176E-101 | CAF-B | EPAS1 |
| 2.2585E-105 | 1.354972102 | 0.807 | 0.284 | 5.6603E-101 | CAF-B | GUCY1B1 |
| 3.1396E-105 | 1.027568936 | 0.554 | 0.105 | 7.8686E-101 | CAF-B | PDE3A |
| 2.286E-104 | 1.508012461 | 0.843 | 0.325 | 5.7291E-100 | CAF-B | HES4 |
| 2.906E-102 | 1.138451345 | 0.521 | 0.095 | 7.28311E-98 | CAF-B | COL5A3 |

| | | | | | | |
|---|---|---|---|---|---|---|
| 2.2257E-100 | 1.644398129 | 0.767 | 0.246 | 5.57806E-96 | CAF-B | ID4 |
| 2.746E-100 | 1.577079384 | 0.974 | 0.568 | 6.88208E-96 | CAF-B | MFGE8 |
| 7.0774E-100 | 1.707124218 | 0.892 | 0.459 | 1.77375E-95 | CAF-B | CRIP2 |
| 1.2937E-99 | 0.874040585 | 0.518 | 0.095 | 3.24219E-95 | CAF-B | SORT1 |
| 3.63378E-98 | 1.17401276 | 0.705 | 0.212 | 9.10697E-94 | CAF-B | DBNDD2 |
| 4.84426E-97 | 0.823748021 | 0.466 | 0.074 | 1.21407E-92 | CAF-B | SYNM |
| 6.54197E-97 | 1.055689952 | 0.597 | 0.142 | 1.63955E-92 | CAF-B | PGF |
| 7.80698E-97 | 1.358709944 | 0.715 | 0.229 | 1.95658E-92 | CAF-B | ISYNA1 |
| 1.51864E-95 | 1.056709784 | 0.626 | 0.163 | 3.80601E-91 | CAF-B | HIP1 |
| 3.68401E-95 | 1.84421034 | 0.984 | 0.726 | 9.23285E-91 | CAF-B | MYL9 |
| 1.01261E-93 | 1.776408296 | 0.954 | 0.634 | 2.53781E-89 | CAF-B | COL4A2 |
| 1.36022E-93 | 1.543526757 | 0.669 | 0.208 | 3.40898E-89 | CAF-B | PLXDC1 |
| 1.41385E-93 | 1.431194574 | 0.77 | 0.281 | 3.54339E-89 | CAF-B | MYO1B |
| 1.95085E-91 | 1.359822592 | 0.872 | 0.423 | 4.88923E-87 | CAF-B | EHD2 |
| 1.63849E-90 | 0.840763403 | 0.456 | 0.079 | 4.10639E-86 | CAF-B | RNF152 |
| 2.28437E-90 | 2.056725856 | 0.951 | 0.586 | 5.72508E-86 | CAF-B | COL4A1 |
| 6.56247E-90 | 0.60432183 | 0.354 | 0.042 | 1.64469E-85 | CAF-B | FRMD3 |
| 2.13438E-89 | 1.553547275 | 0.82 | 0.285 | 5.34919E-85 | CAF-B | ADAMTS1 |
| 2.26486E-89 | 1.429032545 | 0.879 | 0.437 | 5.6762E-85 | CAF-B | ITGA1 |
| 3.59718E-89 | 0.867270611 | 0.295 | 0.028 | 9.01526E-85 | CAF-B | LPL |
| 7.94792E-89 | 0.863565762 | 0.577 | 0.141 | 1.99191E-84 | CAF-B | COBLL1 |
| 1.26742E-88 | 0.818269236 | 0.652 | 0.18 | 3.17642E-84 | CAF-B | KIAA0040 |
| 3.02126E-88 | 0.894111011 | 0.639 | 0.178 | 7.57188E-84 | CAF-B | RASL12 |
| 3.2063E-88 | 1.105796758 | 0.4 | 0.059 | 8.03564E-84 | CAF-B | PLN |
| 1.08383E-87 | 1.004827212 | 0.557 | 0.129 | 2.71628E-83 | CAF-B | AOC3 |
| 3.11993E-87 | 0.756729254 | 0.466 | 0.082 | 7.81916E-83 | CAF-B | CPM |
| 3.17331E-87 | 0.855641674 | 0.466 | 0.089 | 7.95294E-83 | CAF-B | NTN4 |
| 4.50351E-87 | 1.376752425 | 0.692 | 0.227 | 1.12867E-82 | CAF-B | UACA |
| 7.37083E-84 | 0.655793242 | 0.384 | 0.058 | 1.84728E-79 | CAF-B | KLHL23 |
| 2.93172E-83 | 0.67888193 | 0.426 | 0.074 | 7.34747E-79 | CAF-B | ECM2 |
| 3.22122E-83 | 0.853185784 | 0.528 | 0.117 | 8.07303E-79 | CAF-B | NRP1 |
| 7.26965E-83 | 2.001066684 | 0.911 | 0.507 | 1.82192E-78 | CAF-B | CRIP1 |
| 1.17121E-82 | 1.431580845 | 0.879 | 0.38 | 2.93528E-78 | CAF-B | PPP1R14A |
| 7.34899E-82 | 1.177461629 | 0.702 | 0.239 | 1.8418E-77 | CAF-B | EDNRA |
| 1.53306E-81 | 2.351165117 | 0.951 | 0.658 | 3.84216E-77 | CAF-B | ACTA2 |
| 9.92522E-81 | 1.883569531 | 0.81 | 0.388 | 2.48746E-76 | CAF-B | PRSS23 |
| 2.02971E-80 | 0.697288376 | 0.521 | 0.115 | 5.08685E-76 | CAF-B | DAAM2 |
| 8.56207E-80 | 0.961804296 | 0.787 | 0.307 | 2.14583E-75 | CAF-B | TNS1 |

| | | | | | | |
|---|---|---|---|---|---|---|
| 2.50586E-78 | 1.482859034 | 0.807 | 0.328 | 6.28018E-74 | CAF-B | FRZB |
| 8.29064E-78 | 0.732682609 | 0.479 | 0.105 | 2.0778E-73 | CAF-B | ADCY3 |
| 1.36056E-77 | 0.923408164 | 0.702 | 0.217 | 3.40983E-73 | CAF-B | LMOD1 |
| 1.36042E-76 | 1.070865714 | 0.77 | 0.311 | 3.40948E-72 | CAF-B | CAV2 |
| 1.70635E-76 | 0.623367111 | 0.262 | 0.025 | 4.27645E-72 | CAF-B | SNCG |
| 1.48511E-75 | 0.845648068 | 0.348 | 0.051 | 3.72197E-71 | CAF-B | APOLD1 |
| 1.62261E-75 | 0.886930275 | 0.593 | 0.178 | 4.06658E-71 | CAF-B | B3GNT2 |
| 3.56177E-75 | 0.901674372 | 0.603 | 0.173 | 8.92652E-71 | CAF-B | NDRG2 |
| 1.20472E-74 | 1.15055682 | 0.866 | 0.388 | 3.01928E-70 | CAF-B | CAV1 |
| 2.05971E-74 | 0.801261965 | 0.452 | 0.092 | 5.16204E-70 | CAF-B | SGCA |
| 4.79625E-74 | 0.644402595 | 0.37 | 0.062 | 1.20204E-69 | CAF-B | NR2F2-AS1 |
| 5.20852E-74 | 1.053939595 | 0.469 | 0.107 | 1.30536E-69 | CAF-B | OLFML2A |
| 3.19311E-72 | 0.889312148 | 0.997 | 0.807 | 8.00258E-68 | CAF-B | CALD1 |
| 1.3037E-71 | 0.974674378 | 0.751 | 0.328 | 3.26734E-67 | CAF-B | SORBS3 |
| 2.48332E-71 | 1.223404067 | 0.882 | 0.533 | 6.2237E-67 | CAF-B | STOM |
| 1.52582E-69 | 0.618185057 | 0.351 | 0.055 | 3.82401E-65 | CAF-B | KCNMB1 |
| 1.70855E-69 | 0.610462913 | 0.439 | 0.097 | 4.28197E-65 | CAF-B | PLEKHH3 |
| 3.81847E-69 | 1.209610721 | 0.475 | 0.109 | 9.56985E-65 | CAF-B | IGFBP2 |
| 2.77967E-68 | 0.934180213 | 0.695 | 0.245 | 6.9664E-64 | CAF-B | SYNPO2 |
| 4.90935E-68 | 1.255831817 | 0.882 | 0.445 | 1.23038E-63 | CAF-B | MYLK |
| 8.03618E-68 | 0.879768837 | 0.62 | 0.207 | 2.01403E-63 | CAF-B | LURAP1L |
| 1.44089E-67 | 0.715762213 | 0.521 | 0.134 | 3.61115E-63 | CAF-B | LAMA5 |
| 2.38397E-66 | 0.626457637 | 0.292 | 0.041 | 5.97472E-62 | CAF-B | GPAT2 |
| 4.87495E-65 | 2.045103659 | 0.892 | 0.49 | 1.22176E-60 | CAF-B | SPARCL1 |
| 9.62111E-65 | 1.133292093 | 0.275 | 0.036 | 2.41124E-60 | CAF-B | STEAP4 |
| 3.45329E-63 | 0.600355769 | 0.443 | 0.105 | 8.65463E-59 | CAF-B | FRMD4A |
| 1.03651E-62 | 0.710132877 | 0.492 | 0.135 | 2.5977E-58 | CAF-B | PLXND1 |
| 5.3576E-62 | 1.602096034 | 0.587 | 0.185 | 1.34272E-57 | CAF-B | DEPP1 |
| 9.21068E-62 | 0.742571769 | 0.469 | 0.121 | 2.30838E-57 | CAF-B | SPECC1 |
| 2.44416E-61 | 0.647927114 | 0.577 | 0.181 | 6.12555E-57 | CAF-B | SYTL2 |
| 3.30111E-60 | 1.059125442 | 0.961 | 0.541 | 8.27325E-56 | CAF-B | BGN |
| 1.34124E-59 | 0.840257072 | 0.574 | 0.192 | 3.36141E-55 | CAF-B | ARHGAP15 |
| 6.49679E-59 | 0.825392944 | 0.672 | 0.273 | 1.62822E-54 | CAF-B | LRRC32 |
| 1.39149E-58 | 0.787127422 | 0.669 | 0.252 | 3.48736E-54 | CAF-B | MRVI1 |
| 2.12536E-58 | 1.493786168 | 0.305 | 0.051 | 5.32657E-54 | CAF-B | CD36 |
| 1.11623E-57 | 1.373320377 | 0.83 | 0.494 | 2.79749E-53 | CAF-B | CYTOR |
| 2.96656E-57 | 1.840936774 | 0.882 | 0.603 | 7.4348E-53 | CAF-B | TAGLN |
| 9.67197E-56 | 0.918368467 | 0.63 | 0.256 | 2.42399E-51 | CAF-B | MAPRE2 |

| | | | | | | |
|---|---|---|---|---|---|---|
| 3.61047E-55 | 0.988637034 | 0.793 | 0.414 | 9.04857E-51 | CAF-B | PDLIM5 |
| 6.2206E-55 | 1.23692613 | 0.911 | 0.578 | 1.55901E-50 | CAF-B | ID3 |
| 8.33615E-55 | 1.202610208 | 0.843 | 0.513 | 2.08921E-50 | CAF-B | CAVIN3 |
| 8.73804E-55 | 0.782193524 | 0.626 | 0.228 | 2.18993E-50 | CAF-B | RASL11A |
| 2.80194E-54 | 0.976211014 | 0.839 | 0.455 | 7.02223E-50 | CAF-B | KLF9 |
| 1.00484E-53 | 0.88704663 | 0.734 | 0.388 | 2.51833E-49 | CAF-B | UBA2 |
| 1.00616E-53 | 0.963035591 | 0.298 | 0.055 | 2.52164E-49 | CAF-B | SORBS2 |
| 5.59205E-53 | 0.655539794 | 0.479 | 0.143 | 1.40148E-48 | CAF-B | AFAP1L2 |
| 6.72102E-53 | 0.965127636 | 0.662 | 0.285 | 1.68442E-48 | CAF-B | MTHFD2 |
| 6.93868E-53 | 0.908085082 | 0.58 | 0.226 | 1.73897E-48 | CAF-B | ZFHX3 |
| 3.1667E-51 | 0.913538237 | 0.833 | 0.475 | 7.93639E-47 | CAF-B | TGFB1I1 |
| 3.16439E-50 | 0.703818034 | 0.439 | 0.124 | 7.93059E-46 | CAF-B | TESC |
| 7.77883E-50 | 0.949316425 | 0.948 | 0.792 | 1.94953E-45 | CAF-B | IFITM2 |
| 8.06793E-50 | 1.012374273 | 0.816 | 0.453 | 2.02198E-45 | CAF-B | LBH |
| 4.05261E-49 | 0.91136663 | 0.744 | 0.444 | 1.01566E-44 | CAF-B | PPP1R12A |
| 2.01487E-48 | 0.735928303 | 0.62 | 0.266 | 5.04966E-44 | CAF-B | ADGRA2 |
| 2.22132E-48 | 0.72771466 | 0.551 | 0.21 | 5.56708E-44 | CAF-B | MEF2D |
| 3.05642E-48 | 0.673258988 | 0.669 | 0.304 | 7.65999E-44 | CAF-B | NBEAL1 |
| 3.37757E-48 | 0.83893695 | 0.898 | 0.696 | 8.46486E-44 | CAF-B | 7-Sep |
| 5.80548E-48 | 0.915686285 | 0.925 | 0.688 | 1.45497E-43 | CAF-B | ITGB1 |
| 7.89555E-48 | 0.848656302 | 0.557 | 0.21 | 1.97878E-43 | CAF-B | HSPA2 |
| 1.51744E-47 | 1.202795095 | 0.37 | 0.092 | 3.80301E-43 | CAF-B | MT1M |
| 1.8949E-47 | 1.020669591 | 0.525 | 0.173 | 4.749E-43 | CAF-B | ASPN |
| 5.66927E-47 | 0.964322167 | 0.882 | 0.609 | 1.42083E-42 | CAF-B | CD59 |
| 1.62433E-46 | 0.620741447 | 0.567 | 0.221 | 4.07089E-42 | CAF-B | PDE5A |
| 1.98079E-46 | 0.875937174 | 0.708 | 0.374 | 4.96426E-42 | CAF-B | MAP3K20 |
| 7.14805E-46 | 0.94559307 | 0.761 | 0.414 | 1.79144E-41 | CAF-B | NUDT4 |
| 7.4582E-46 | 0.766601705 | 0.656 | 0.321 | 1.86917E-41 | CAF-B | ATP2B4 |
| 1.53699E-45 | 0.759206529 | 0.744 | 0.382 | 3.85201E-41 | CAF-B | LAMB2 |
| 1.73046E-45 | 0.698016418 | 0.623 | 0.277 | 4.33688E-41 | CAF-B | KIRREL1 |
| 5.12953E-45 | 0.863702752 | 0.836 | 0.494 | 1.28556E-40 | CAF-B | CAVIN1 |
| 5.87672E-45 | 0.754362847 | 0.63 | 0.299 | 1.47282E-40 | CAF-B | ARHGAP1 |
| 8.43888E-45 | 0.878837825 | 0.639 | 0.302 | 2.11495E-40 | CAF-B | PLS3 |
| 9.32408E-45 | 1.066491783 | 0.885 | 0.578 | 2.3368E-40 | CAF-B | TSC22D1 |
| 1.52775E-44 | 0.911906327 | 0.974 | 0.698 | 3.82883E-40 | CAF-B | TPM1 |
| 9.99623E-44 | 0.714051851 | 0.561 | 0.23 | 2.50525E-39 | CAF-B | ARHGEF7 |
| 1.67544E-43 | 0.715993751 | 0.685 | 0.323 | 4.19898E-39 | CAF-B | DLC1 |
| 2.10265E-43 | 1.260484875 | 0.692 | 0.352 | 5.26965E-39 | CAF-B | CD248 |

| | | | | | | |
|---|---|---|---|---|---|---|
| 7.44524E-43 | 0.615633884 | 0.761 | 0.432 | 1.86593E-38 | CAF-B | PTEN |
| 1.19846E-42 | 0.734890687 | 0.57 | 0.259 | 3.00357E-38 | CAF-B | OAZ2 |
| 1.30009E-42 | 0.800701984 | 0.99 | 0.952 | 3.25829E-38 | CAF-B | VIM |
| 1.40152E-42 | 0.722925379 | 0.816 | 0.508 | 3.5125E-38 | CAF-B | TACC1 |
| 2.36976E-42 | 0.735354879 | 0.597 | 0.26 | 5.93908E-38 | CAF-B | ARHGAP10 |
| 7.86181E-42 | 0.65903367 | 0.659 | 0.306 | 1.97033E-37 | CAF-B | PRKG1 |
| 1.37685E-41 | 0.830640979 | 0.551 | 0.225 | 3.45067E-37 | CAF-B | CRIM1 |
| 2.06286E-41 | 0.79046097 | 0.466 | 0.156 | 5.16995E-37 | CAF-B | CNN1 |
| 3.02779E-41 | 0.656773588 | 0.567 | 0.251 | 7.58826E-37 | CAF-B | ARHGAP17 |
| 4.01353E-41 | 0.755952445 | 0.797 | 0.413 | 1.00587E-36 | CAF-B | COL14A1 |
| 5.91138E-41 | 0.696165198 | 0.61 | 0.279 | 1.48151E-36 | CAF-B | CRYAB |
| 1.20343E-40 | 0.77142032 | 0.889 | 0.652 | 3.01603E-36 | CAF-B | ACTN4 |
| 1.57116E-40 | 1.052314565 | 0.728 | 0.437 | 3.93763E-36 | CAF-B | MIR4435-2HG |
| 2.32406E-40 | 0.848404954 | 0.862 | 0.549 | 5.82457E-36 | CAF-B | PHLDA1 |
| 3.02485E-40 | 0.698420337 | 0.728 | 0.432 | 7.58087E-36 | CAF-B | RSU1 |
| 5.08263E-40 | 0.713370681 | 0.728 | 0.393 | 1.27381E-35 | CAF-B | FBLIM1 |
| 6.46375E-40 | 0.775766657 | 0.852 | 0.589 | 1.61994E-35 | CAF-B | MGST3 |
| 9.25126E-40 | 0.732396255 | 0.931 | 0.673 | 2.31855E-35 | CAF-B | LMNA |
| 1.41447E-39 | 0.631610477 | 0.548 | 0.226 | 3.54494E-35 | CAF-B | PLEKHG2 |
| 1.91196E-39 | 1.283751799 | 0.882 | 0.712 | 4.79176E-35 | CAF-B | DSTN |
| 2.11261E-39 | 0.638248145 | 0.446 | 0.158 | 5.29463E-35 | CAF-B | FADS3 |
| 3.3355E-39 | 0.70029477 | 0.774 | 0.472 | 8.35943E-35 | CAF-B | CHCHD10 |
| 1.33486E-38 | 0.921208337 | 0.911 | 0.702 | 3.34542E-34 | CAF-B | FLNA |
| 2.97332E-38 | 0.641928748 | 0.597 | 0.271 | 7.45174E-34 | CAF-B | UTRN |
| 3.62859E-38 | 0.632732554 | 0.282 | 0.067 | 9.09397E-34 | CAF-B | ACTA2-AS1 |
| 5.02047E-38 | 0.73462874 | 0.744 | 0.414 | 1.25823E-33 | CAF-B | PMEPA1 |
| 5.41288E-38 | 0.714582843 | 0.964 | 0.622 | 1.35658E-33 | CAF-B | A2M |
| 7.17366E-38 | 1.002621224 | 0.521 | 0.238 | 1.79786E-33 | CAF-B | C20orf27 |
| 8.7424E-38 | 0.985321595 | 0.672 | 0.362 | 2.19102E-33 | CAF-B | PCSK7 |
| 2.21652E-37 | 0.614720764 | 0.652 | 0.311 | 5.55505E-33 | CAF-B | ENAH |
| 2.45124E-37 | 0.824665376 | 0.41 | 0.142 | 6.14329E-33 | CAF-B | NET1 |
| 3.88306E-37 | 1.141250159 | 0.813 | 0.56 | 9.73172E-33 | CAF-B | CSRP1 |
| 6.51648E-37 | 0.741195194 | 0.521 | 0.212 | 1.63316E-32 | CAF-B | SGIP1 |
| 6.76839E-37 | 0.736799621 | 0.889 | 0.663 | 1.69629E-32 | CAF-B | TLN1 |
| 8.31784E-37 | 0.661418893 | 0.921 | 0.763 | 2.08462E-32 | CAF-B | SELENOW |
| 1.03461E-36 | 0.665981956 | 0.879 | 0.621 | 2.59293E-32 | CAF-B | CYB5R3 |
| 1.38057E-36 | 0.976921242 | 0.951 | 0.733 | 3.45999E-32 | CAF-B | TPM2 |
| 2.46201E-36 | 0.705306541 | 0.928 | 0.551 | 6.17029E-32 | CAF-B | C11orf96 |

| | | | | | | |
|---|---|---|---|---|---|---|
| 4.59161E-36 | 0.679919466 | 0.793 | 0.478 | 1.15075E-31 | CAF-B | VCL |
| 1.35189E-35 | 0.838185099 | 0.534 | 0.238 | 3.3881E-31 | CAF-B | CAMK2N1 |
| 3.39758E-35 | 0.634341383 | 0.528 | 0.22 | 8.51501E-31 | CAF-B | FBXO32 |
| 4.10871E-35 | 0.734739166 | 0.849 | 0.561 | 1.02972E-30 | CAF-B | CNN3 |
| 8.16689E-35 | 0.671340187 | 0.984 | 0.838 | 2.04679E-30 | CAF-B | IFITM3 |
| 9.91922E-35 | 0.762416801 | 0.616 | 0.301 | 2.48596E-30 | CAF-B | FKBP5 |
| 1.28918E-34 | 0.702472296 | 0.403 | 0.138 | 3.23095E-30 | CAF-B | HSPB8 |
| 3.0461E-34 | 0.611419885 | 0.482 | 0.192 | 7.63415E-30 | CAF-B | NES |
| 3.58551E-33 | 0.800463976 | 0.879 | 0.644 | 8.98601E-29 | CAF-B | CD151 |
| 6.97369E-33 | 0.618679399 | 0.941 | 0.817 | 1.74775E-28 | CAF-B | CALM2 |
| 2.83491E-32 | 0.720276235 | 1 | 0.957 | 7.10485E-28 | CAF-B | MALAT1 |
| 5.62187E-32 | 0.660038465 | 0.83 | 0.535 | 1.40895E-27 | CAF-B | LPP |
| 1.97063E-31 | 0.617729876 | 0.623 | 0.32 | 4.9388E-27 | CAF-B | MSRB3 |
| 6.72359E-30 | 0.672559765 | 0.708 | 0.379 | 1.68507E-25 | CAF-B | PDLIM3 |
| 3.11902E-29 | 0.611289359 | 0.416 | 0.172 | 7.81688E-25 | CAF-B | EOGT |
| 8.26573E-29 | 0.717192918 | 0.767 | 0.455 | 2.07156E-24 | CAF-B | CRISPLD2 |
| 9.67313E-29 | 0.680008344 | 0.682 | 0.411 | 2.42428E-24 | CAF-B | ASAH1 |
| 3.09026E-28 | 0.749309521 | 0.721 | 0.44 | 7.74481E-24 | CAF-B | SDC2 |
| 3.1005E-28 | 0.658166192 | 0.774 | 0.58 | 7.77047E-24 | CAF-B | ANXA6 |
| 1.70339E-27 | 0.608657258 | 0.977 | 0.948 | 4.26903E-23 | CAF-B | MYL6 |
| 2.26422E-27 | 0.699263006 | 0.784 | 0.488 | 5.6746E-23 | CAF-B | TFPI |
| 2.49859E-27 | 0.710479073 | 0.328 | 0.112 | 6.26196E-23 | CAF-B | RASD1 |
| 5.59788E-26 | 0.60813825 | 0.892 | 0.583 | 1.40294E-21 | CAF-B | FILIP1L |
| 7.52683E-26 | 0.739808997 | 0.623 | 0.341 | 1.88638E-21 | CAF-B | PHLDA2 |
| 1.00231E-25 | 0.624904304 | 0.584 | 0.327 | 2.51199E-21 | CAF-B | PEAK1 |
| 1.02122E-25 | 0.654244923 | 0.757 | 0.519 | 2.55939E-21 | CAF-B | ANKRD10 |
| 4.76136E-25 | 0.637498389 | 0.682 | 0.438 | 1.19329E-20 | CAF-B | ROCK1 |
| 1.60156E-23 | 0.829992639 | 0.551 | 0.296 | 4.01384E-19 | CAF-B | MYC |
| 2.09866E-23 | 0.652518111 | 0.38 | 0.163 | 5.25966E-19 | CAF-B | BCL6 |
| 2.76683E-23 | 0.637586803 | 0.393 | 0.163 | 6.93423E-19 | CAF-B | HSPB6 |
| 3.95336E-23 | 0.620465184 | 0.603 | 0.341 | 9.90791E-19 | CAF-B | VASN |
| 1.0413E-21 | 0.690029894 | 0.449 | 0.223 | 2.60971E-17 | CAF-B | TIPARP |
| 1.27976E-21 | 1.341475014 | 0.689 | 0.445 | 3.20734E-17 | CAF-B | NR4A1 |
| 1.35405E-21 | 0.756081653 | 0.302 | 0.108 | 3.39353E-17 | CAF-B | RRAD |
| 1.04621E-20 | 0.627433371 | 0.436 | 0.206 | 2.62201E-16 | CAF-B | WFDC1 |
| 3.14289E-20 | 0.767315769 | 0.561 | 0.327 | 7.87671E-16 | CAF-B | LTBP1 |
| 1.96399E-19 | 0.81746287 | 0.603 | 0.363 | 4.92214E-15 | CAF-B | MT1E |
| 5.79008E-19 | 0.635692044 | 0.636 | 0.419 | 1.45111E-14 | CAF-B | NID1 |

| | | | | | | |
|---|---|---|---|---|---|---|
| 7.82282E-19 | 0.875695017 | 0.456 | 0.231 | 1.96056E-14 | CAF-B | ACTG2 |
| 2.00342E-18 | 0.638142941 | 0.603 | 0.366 | 5.02098E-14 | CAF-B | C1QTNF5 |
| 3.88282E-18 | 0.742847195 | 0.662 | 0.435 | 9.73113E-14 | CAF-B | CDKN1A |
| 5.48485E-15 | 0.640459889 | 0.81 | 0.607 | 1.37461E-10 | CAF-B | GADD45B |
| 6.88138E-15 | 0.66230871 | 0.774 | 0.543 | 1.72461E-10 | CAF-B | ATF3 |
| 3.00384E-14 | 0.654487401 | 0.938 | 0.821 | 7.52824E-10 | CAF-B | JUNB |
| 4.68307E-14 | 0.759096133 | 0.695 | 0.525 | 1.17367E-09 | CAF-B | THY1 |
| 1.6317E-13 | 0.858816637 | 0.764 | 0.529 | 4.08935E-09 | CAF-B | THBS1 |
| 2.4984E-13 | 0.675149469 | 0.492 | 0.302 | 6.26149E-09 | CAF-B | AL133415.1 |
| 3.14659E-09 | 0.671370726 | 0.793 | 0.641 | 7.88599E-05 | CAF-B | S100A4 |
| 6.20779E-06 | 0.683994966 | 0.39 | 0.261 | 0.155579685 | CAF-B | PDK4 |
| 0 | 3.217071804 | 0.655 | 0.023 | 0 | CAF-D | PLVAP |
| 7.3885E-278 | 2.961007715 | 0.471 | 0.006 | 1.8517E-273 | CAF-D | CLCA4 |
| 2.7143E-223 | 2.436965445 | 0.412 | 0.008 | 6.8027E-219 | CAF-D | CLDN5 |
| 9.2559E-218 | 3.136895716 | 0.514 | 0.025 | 2.3197E-213 | CAF-D | SLC26A3 |
| 1.1497E-179 | 2.368706526 | 0.439 | 0.022 | 2.8814E-175 | CAF-D | GUCA2A |
| 4.2435E-176 | 2.631592692 | 0.424 | 0.02 | 1.0635E-171 | CAF-D | CEACAM7 |
| 3.0954E-155 | 2.695681685 | 0.514 | 0.05 | 7.7576E-151 | CAF-D | PECAM1 |
| 2.4504E-150 | 2.270646513 | 0.451 | 0.035 | 6.1411E-146 | CAF-D | RNASE1 |
| 4.0667E-142 | 2.463777079 | 0.498 | 0.052 | 1.0192E-137 | CAF-D | FLT1 |
| 1.4555E-137 | 3.333615706 | 0.553 | 0.074 | 3.6478E-133 | CAF-D | KRT20 |
| 1.1775E-127 | 2.406096611 | 0.365 | 0.025 | 2.951E-123 | CAF-D | CEACAM1 |
| 9.9445E-119 | 1.756302373 | 0.286 | 0.013 | 2.4923E-114 | CAF-D | VWF |
| 3.9587E-117 | 1.497805383 | 0.231 | 0.006 | 9.9214E-113 | CAF-D | CDH5 |
| 8.3195E-112 | 2.285227761 | 0.388 | 0.037 | 2.085E-107 | CAF-D | MUC13 |
| 2.1363E-110 | 3.629027406 | 0.635 | 0.144 | 5.3539E-106 | CAF-D | PIGR |
| 1.6042E-109 | 2.711901179 | 0.502 | 0.077 | 4.0204E-105 | CAF-D | FXYD3 |
| 2.4451E-109 | 2.081896755 | 0.314 | 0.021 | 6.1279E-105 | CAF-D | CA1 |
| 1.2821E-107 | 2.888368166 | 0.549 | 0.099 | 3.2133E-103 | CAF-D | CEACAM5 |
| 1.2091E-103 | 3.410105063 | 0.773 | 0.274 | 3.0302E-99 | CAF-D | PHGR1 |
| 1.2261E-103 | 2.500795222 | 0.392 | 0.044 | 3.0729E-99 | CAF-D | LAMA3 |
| 1.9814E-102 | 2.336311312 | 0.396 | 0.046 | 4.9659E-98 | CAF-D | ENPP2 |
| 1.5877E-100 | 1.471300595 | 0.251 | 0.012 | 3.97902E-96 | CAF-D | ADGRL4 |
| 1.11582E-94 | 3.478898222 | 0.729 | 0.258 | 2.79646E-90 | CAF-D | FABP1 |
| 1.24067E-93 | 2.088155251 | 0.392 | 0.049 | 3.10936E-89 | CAF-D | TSPAN1 |
| 6.53332E-93 | 1.482025859 | 0.588 | 0.12 | 1.63738E-88 | CAF-D | CCL4 |
| 1.26027E-92 | 2.211197624 | 0.922 | 0.565 | 3.1585E-88 | CAF-D | CD74 |
| 2.51292E-92 | 1.902040031 | 0.286 | 0.022 | 6.29787E-88 | CAF-D | DSC2 |

| | | | | | | |
|---|---|---|---|---|---|---|
| 2.13278E-91 | 2.293888747 | 0.455 | 0.075 | 5.34516E-87 | CAF-D | CEACAM6 |
| 6.20215E-85 | 2.037834525 | 0.329 | 0.037 | 1.55438E-80 | CAF-D | PLAC8 |
| 1.67942E-80 | 2.321337612 | 0.584 | 0.154 | 4.20897E-76 | CAF-D | EPCAM |
| 1.93416E-80 | 3.28112006 | 0.647 | 0.222 | 4.84739E-76 | CAF-D | CKB |
| 3.35691E-78 | 0.932335887 | 0.796 | 0.232 | 8.41309E-74 | CAF-D | RGS5 |
| 2.82508E-77 | 1.829584665 | 0.784 | 0.317 | 7.08022E-73 | CAF-D | IGFBP3 |
| 3.14816E-77 | 1.746309444 | 0.271 | 0.025 | 7.88992E-73 | CAF-D | PLS1 |
| 6.91897E-77 | 2.071046172 | 0.875 | 0.524 | 1.73403E-72 | CAF-D | SELENOP |
| 9.28978E-77 | 1.24319212 | 0.204 | 0.011 | 2.3282E-72 | CAF-D | PODXL |
| 1.30937E-74 | 1.81153445 | 0.29 | 0.032 | 3.28153E-70 | CAF-D | MYH14 |
| 2.09814E-74 | 1.465417184 | 0.361 | 0.05 | 5.25837E-70 | CAF-D | CD36 |
| 4.95115E-74 | 2.034170673 | 0.408 | 0.073 | 1.24086E-69 | CAF-D | TSPAN8 |
| 2.30388E-73 | 1.295619267 | 0.212 | 0.014 | 5.77398E-69 | CAF-D | CYYR1 |
| 3.82202E-72 | 2.252207005 | 0.529 | 0.138 | 9.57876E-68 | CAF-D | CLDN4 |
| 8.05486E-70 | 1.184661416 | 0.204 | 0.014 | 2.01871E-65 | CAF-D | CD93 |
| 3.69069E-69 | 1.813351538 | 0.345 | 0.053 | 9.2496E-65 | CAF-D | ITGA6 |
| 2.54418E-64 | 2.199572319 | 0.541 | 0.159 | 6.37623E-60 | CAF-D | KRT19 |
| 6.09403E-63 | 3.166580325 | 0.671 | 0.305 | 1.52729E-58 | CAF-D | MUC12 |
| 6.34333E-58 | 1.892757941 | 0.349 | 0.067 | 1.58977E-53 | CAF-D | CA2 |
| 4.13673E-57 | 2.067426703 | 0.541 | 0.176 | 1.03675E-52 | CAF-D | HLA-DRA |
| 2.20841E-56 | 2.595565129 | 0.702 | 0.374 | 5.53472E-52 | CAF-D | KRT8 |
| 2.79654E-53 | 1.493443446 | 0.227 | 0.028 | 7.0087E-49 | CAF-D | GPA33 |
| 3.11425E-51 | 1.841049073 | 0.216 | 0.026 | 7.80493E-47 | CAF-D | C1QA |
| 3.71283E-49 | 2.016568974 | 0.514 | 0.181 | 9.30509E-45 | CAF-D | LGALS4 |
| 1.55139E-46 | 1.353749578 | 0.631 | 0.248 | 3.8881E-42 | CAF-D | SRGN |
| 7.30482E-46 | 1.69226936 | 0.412 | 0.117 | 1.83073E-41 | CAF-D | CLDN3 |
| 9.3595E-45 | 1.353561251 | 0.306 | 0.063 | 2.34568E-40 | CAF-D | C15orf48 |
| 8.75196E-44 | 2.258449015 | 0.478 | 0.187 | 2.19342E-39 | CAF-D | CD320 |
| 2.00205E-43 | 1.643253917 | 0.318 | 0.076 | 5.01755E-39 | CAF-D | RAMP2 |
| 2.45207E-43 | 2.174212167 | 0.525 | 0.207 | 6.14538E-39 | CAF-D | HLA-DRB1 |
| 3.29109E-42 | 2.287439639 | 0.486 | 0.197 | 8.24814E-38 | CAF-D | LMO7 |
| 9.33419E-42 | 1.61582157 | 0.8 | 0.626 | 2.33934E-37 | CAF-D | IFI27 |
| 1.34038E-41 | 1.317602091 | 0.216 | 0.033 | 3.35926E-37 | CAF-D | AIF1 |
| 2.44399E-41 | 1.776324674 | 0.216 | 0.033 | 6.12512E-37 | CAF-D | C1QC |
| 5.93349E-40 | 1.819066238 | 0.302 | 0.072 | 1.48705E-35 | CAF-D | GPRC5A |
| 8.87341E-39 | 1.869380964 | 0.416 | 0.142 | 2.22385E-34 | CAF-D | HLA-DRB5 |
| 1.9417E-38 | 1.448578574 | 0.322 | 0.08 | 4.86628E-34 | CAF-D | TYROBP |
| 2.34699E-36 | 1.490529212 | 0.647 | 0.374 | 5.88202E-32 | CAF-D | FABP5 |

| | | | | | | |
|---|---|---|---|---|---|---|
| 3.00143E-36 | 1.446462877 | 0.525 | 0.23 | 7.52219E-32 | CAF-D | TFF3 |
| 3.71117E-34 | 1.495983782 | 0.227 | 0.046 | 9.30093E-30 | CAF-D | CDH1 |
| 8.47414E-33 | 1.273292431 | 0.216 | 0.043 | 2.12379E-28 | CAF-D | CD34 |
| 4.15311E-32 | 1.574472398 | 0.278 | 0.073 | 1.04085E-27 | CAF-D | DSP |
| 4.8876E-31 | 1.400706406 | 0.22 | 0.047 | 1.22493E-26 | CAF-D | CDH17 |
| 5.68698E-31 | 1.317628054 | 0.42 | 0.164 | 1.42527E-26 | CAF-D | CLEC3B |
| 1.58943E-30 | 1.283173877 | 0.247 | 0.061 | 3.98343E-26 | CAF-D | EGFL7 |
| 2.13856E-30 | 1.823321097 | 0.4 | 0.166 | 5.35966E-26 | CAF-D | CAVIN2 |
| 9.77386E-30 | 0.907335595 | 0.788 | 0.606 | 2.44952E-25 | CAF-D | COL4A1 |
| 6.67788E-29 | 1.221387151 | 0.796 | 0.721 | 1.67361E-24 | CAF-D | AHNAK |
| 9.28811E-29 | 1.252336055 | 0.239 | 0.06 | 2.32779E-24 | CAF-D | INSR |
| 2.06807E-28 | 1.518671337 | 0.286 | 0.086 | 5.183E-24 | CAF-D | CLDN7 |
| 5.71184E-28 | 1.711653689 | 0.447 | 0.208 | 1.4315E-23 | CAF-D | CD24 |
| 3.32636E-25 | 1.004837008 | 0.784 | 0.689 | 8.33653E-21 | CAF-D | KLF6 |
| 5.43071E-25 | 1.464424022 | 0.286 | 0.095 | 1.36104E-20 | CAF-D | MXD1 |
| 1.71907E-24 | 0.827050338 | 0.831 | 0.791 | 4.30833E-20 | CAF-D | S100A10 |
| 3.48592E-24 | 1.44915743 | 0.392 | 0.179 | 8.73641E-20 | CAF-D | SGK1 |
| 4.16492E-24 | 1.863301478 | 0.541 | 0.363 | 1.04381E-19 | CAF-D | EZR |
| 6.69639E-23 | 1.133522324 | 0.286 | 0.099 | 1.67825E-18 | CAF-D | ESAM |
| 2.61229E-22 | 1.45441842 | 0.702 | 0.672 | 6.54691E-18 | CAF-D | ACTN4 |
| 3.34842E-22 | 1.134054296 | 0.224 | 0.065 | 8.39182E-18 | CAF-D | CALCRL |
| 6.31658E-22 | 1.358280129 | 0.271 | 0.095 | 1.58306E-17 | CAF-D | EFNB2 |
| 1.3315E-21 | 1.338087947 | 0.282 | 0.1 | 3.33701E-17 | CAF-D | ELF3 |
| 2.10087E-21 | 1.101996637 | 0.459 | 0.23 | 5.26519E-17 | CAF-D | MCAM |
| 2.16972E-21 | 0.692807525 | 0.745 | 0.509 | 5.43774E-17 | CAF-D | SPARCL1 |
| 4.02416E-20 | 1.353449936 | 0.561 | 0.384 | 1.00854E-15 | CAF-D | KRT18 |
| 1.98709E-19 | 1.415702326 | 0.447 | 0.257 | 4.98006E-15 | CAF-D | TM4SF1 |
| 5.56867E-19 | 1.238130097 | 0.243 | 0.084 | 1.39562E-14 | CAF-D | HLA-DQB1 |
| 1.60367E-18 | 0.926126238 | 0.2 | 0.059 | 4.01912E-14 | CAF-D | HBEGF |
| 1.1587E-17 | 1.31230287 | 0.706 | 0.756 | 2.90392E-13 | CAF-D | LGALS3 |
| 1.59646E-17 | 0.96475507 | 0.31 | 0.13 | 4.00105E-13 | CAF-D | AGR2 |
| 2.88378E-17 | 1.092774995 | 0.573 | 0.415 | 7.22733E-13 | CAF-D | PRSS23 |
| 1.68894E-16 | 0.823877496 | 0.902 | 0.94 | 4.23282E-12 | CAF-D | MT-ND4 |
| 8.31594E-16 | 0.922777084 | 0.204 | 0.069 | 2.08414E-11 | CAF-D | GIMAP4 |
| 8.68724E-16 | 1.190161828 | 0.239 | 0.093 | 2.1772E-11 | CAF-D | SPINT2 |
| 1.33546E-15 | 1.521287623 | 0.435 | 0.295 | 3.34694E-11 | CAF-D | PLAUR |
| 1.78562E-15 | 1.325815967 | 0.349 | 0.184 | 4.47511E-11 | CAF-D | RGCC |
| 1.87087E-15 | 1.27278031 | 0.639 | 0.637 | 4.68877E-11 | CAF-D | SPTBN1 |

| | | | | | | |
|---|---|---|---|---|---|---|
| 2.2556E-15 | 0.769421283 | 0.843 | 0.919 | 5.65299E-11 | CAF-D | MT-ND2 |
| 2.82035E-14 | 0.932136988 | 0.212 | 0.08 | 7.06836E-10 | CAF-D | ADGRF5 |
| 6.71115E-14 | 1.15313451 | 0.231 | 0.099 | 1.68195E-09 | CAF-D | ICAM2 |
| 1.41903E-13 | 0.836164001 | 0.608 | 0.494 | 3.55637E-09 | CAF-D | TNFAIP3 |
| 1.78889E-13 | 0.93857514 | 0.329 | 0.168 | 4.48332E-09 | CAF-D | AREG |
| 3.5497E-13 | 0.992490612 | 0.412 | 0.249 | 8.89626E-09 | CAF-D | CXCR4 |
| 8.68332E-13 | 1.106427981 | 0.62 | 0.59 | 2.17621E-08 | CAF-D | TPM3 |
| 3.18326E-12 | 0.777476841 | 0.698 | 0.745 | 7.97788E-08 | CAF-D | HLA-E |
| 4.41535E-12 | 0.624769594 | 0.588 | 0.457 | 1.10657E-07 | CAF-D | IER3 |
| 6.27412E-12 | 1.639410779 | 0.427 | 0.318 | 1.57242E-07 | CAF-D | SSFA2 |
| 1.12031E-11 | 1.008340596 | 0.357 | 0.206 | 2.80772E-07 | CAF-D | IL7R |
| 1.42311E-11 | 1.050863311 | 0.62 | 0.635 | 3.56659E-07 | CAF-D | CD59 |
| 2.01556E-11 | 0.68969046 | 0.271 | 0.127 | 5.05141E-07 | CAF-D | TINAGL1 |
| 2.18401E-10 | 1.13816303 | 0.208 | 0.097 | 5.47356E-06 | CAF-D | EHD4 |
| 3.28287E-10 | 1.422784024 | 0.267 | 0.151 | 8.22753E-06 | CAF-D | SLC26A2 |
| 5.08185E-10 | 1.093527239 | 0.204 | 0.094 | 1.27361E-05 | CAF-D | ASS1 |
| 1.59402E-09 | 0.742297925 | 0.212 | 0.097 | 3.99494E-05 | CAF-D | HLA-DQA1 |
| 1.69571E-09 | 2.326593095 | 0.235 | 0.123 | 4.24978E-05 | CAF-D | IGKV4-1 |
| 2.1419E-09 | 0.726288397 | 0.667 | 0.672 | 5.36802E-05 | CAF-D | APP |
| 6.20822E-09 | 1.201836744 | 0.463 | 0.408 | 0.00015559 | CAF-D | SLC9A3R2 |
| 7.46641E-09 | 1.342866039 | 0.361 | 0.261 | 0.000187123 | CAF-D | FLNB |
| 1.05847E-08 | 0.621557429 | 0.714 | 0.795 | 0.000265274 | CAF-D | YWHAZ |
| 1.53674E-08 | 0.669795475 | 0.694 | 0.77 | 0.000385138 | CAF-D | ANXA2 |
| 3.51666E-08 | 0.856556271 | 0.553 | 0.525 | 0.000881346 | CAF-D | GNG11 |
| 3.64447E-08 | 1.237886502 | 0.365 | 0.267 | 0.000913377 | CAF-D | HLA-DPA1 |
| 9.00591E-08 | 1.141754238 | 0.231 | 0.133 | 0.002257061 | CAF-D | USP53 |
| 1.47833E-07 | 0.898850294 | 0.2 | 0.107 | 0.003704998 | CAF-D | ARHGAP29 |
| 4.83939E-07 | 1.262713305 | 0.314 | 0.228 | 0.012128481 | CAF-D | SWAP70 |
| 6.6959E-07 | 1.241473208 | 0.369 | 0.296 | 0.016781261 | CAF-D | UTRN |
| 8.21859E-07 | 0.913034946 | 0.49 | 0.423 | 0.020597421 | CAF-D | EMP1 |
| 2.27577E-06 | 0.994850018 | 0.255 | 0.165 | 0.057035362 | CAF-D | UPP1 |
| 2.89401E-06 | 0.982981824 | 0.455 | 0.383 | 0.072529574 | CAF-D | AL138963.3 |
| 3.65087E-06 | 1.173950118 | 0.322 | 0.25 | 0.091498044 | CAF-D | CRIM1 |
| 4.79699E-06 | 0.859758726 | 0.565 | 0.583 | 0.120222136 | CAF-D | PLPP1 |
| 7.21395E-06 | 1.305682182 | 0.29 | 0.217 | 0.180796092 | CAF-D | TMEM54 |
| 1.14612E-05 | 1.282597316 | 0.333 | 0.254 | 0.28723981 | CAF-D | CSRP2 |
| 1.46264E-05 | 0.995968238 | 0.376 | 0.316 | 0.366567801 | CAF-D | HLA-DPB1 |
| 4.52633E-05 | 1.191835191 | 0.212 | 0.143 | 1 | CAF-D | CES2 |

| | | | | | | |
|---|---|---|---|---|---|---|
| 6.24037E-05 | 1.050654547 | 0.467 | 0.454 | 1 | CAF-D | CD55 |
| 0.000110701 | 0.68479977 | 0.498 | 0.5 | 1 | CAF-D | CRIP2 |
| 0.000143167 | 0.802369202 | 0.447 | 0.416 | 1 | CAF-D | CD9 |
| 0.000242444 | 0.651046167 | 0.463 | 0.431 | 1 | CAF-D | CAV1 |
| 0.000390383 | 0.9352745 | 0.208 | 0.149 | 1 | CAF-D | CD68 |
| 0.000592477 | 0.757829393 | 0.204 | 0.143 | 1 | CAF-D | ADAMTS9 |
| 0.000768364 | 0.875206125 | 0.2 | 0.144 | 1 | CAF-D | ADAM15 |
| 0.000881395 | 1.071946259 | 0.467 | 0.526 | 1 | CAF-D | TGFBR2 |
| 0.000916295 | 1.086518663 | 0.325 | 0.299 | 1 | CAF-D | SEC14L1 |
| 0.001013536 | 0.946456122 | 0.388 | 0.372 | 1 | CAF-D | EPAS1 |
| 0.001270451 | 0.86340526 | 0.502 | 0.564 | 1 | CAF-D | ITM2C |
| 0.001966286 | 0.94300875 | 0.204 | 0.155 | 1 | CAF-D | ATP1B1 |
| 0.002112723 | 0.982276023 | 0.227 | 0.181 | 1 | CAF-D | SLC40A1 |
| 0.002330637 | 1.055215989 | 0.31 | 0.282 | 1 | CAF-D | SYNE2 |
| 0.003174918 | 0.905652763 | 0.396 | 0.422 | 1 | CAF-D | ETS1 |
| 0.004210941 | 0.650170898 | 0.31 | 0.271 | 1 | CAF-D | COL15A1 |
| 0.004784414 | 0.995347335 | 0.286 | 0.256 | 1 | CAF-D | JUP |
| 0.005069833 | 0.912209064 | 0.31 | 0.289 | 1 | CAF-D | CTSS |
| 0.005383111 | 0.635355963 | 0.565 | 0.691 | 1 | CAF-D | RHOC |
| 0.006739557 | 0.967707139 | 0.227 | 0.187 | 1 | CAF-D | BIRC3 |
| 0.007595419 | 0.829297155 | 0.231 | 0.194 | 1 | CAF-D | MTUS1 |
| 0.009343187 | 0.861517788 | 0.435 | 0.481 | 1 | CAF-D | PLEC |
